# Lessons Learned from Implementing Injury and Illness Surveillance in Professional Football: Introducing a New Implementation Framework

**DOI:** 10.1007/s40279-025-02276-5

**Published:** 2025-07-11

**Authors:** Montassar Tabben, Bahar Hassanmirzaei, Gurcharan Singh, Riadh Miladi, Mokhtar Chaabane, Pierre McCourt, Andreas Serner, Ben Clarsen, Zied Ellouze, Mourad Mokrani, Rodney Whiteley, Pieter D‘Hooghe, Marco Cardinale, Yorck Olaf Schumacher, Roald Bahr

**Affiliations:** 1 Aspetar, Orthopaedic and Sports Medicine Hospital, Doha, Qatar; 2 Oslo Sports Trauma Research Center, Institute of Sports Medicine, Norwegian School of Sports Sciences, Oslo, Norway; 3 Sports Medicine Unit, Asian Football Confederation, Kuala Lumpur, Malaysia; 4 Federation Internationale de Football Association, Zurich, Switzerland; 5 Ministry of Sport, Riyadh, Saudi Arabia

## Abstract

Injury and illness surveillance (IIS) systems play a crucial role in professional sports by monitoring athlete health, informing preventive strategies, and enhancing performance. Although international bodies, such as the International Olympic Committee, have established comprehensive guidelines, implementing these standards across diverse sports environments and specifically in football (soccer), remains challenging. This paper reflects on the experiences gained from implementing IIS systems in various football settings, including the Qatar Stars League and the Asian Football Confederation. It explores the successes and challenges encountered while adapting surveillance protocols to specific contexts and introduces a framework aimed at standardizing and optimizing IIS implementation. The findings underscore the importance of centralized oversight, routine data collection, and stable staffing to ensure consistent data quality. The integration of electronic data tools and real-time monitoring has further improved data accuracy and reporting. However, maintaining engagement across large geographical regions and addressing regulatory variations continue to pose challenges. We propose a step-by-step framework for IIS implementation that addresses these complexities while providing a strong foundation for expanding surveillance across other sports settings. This framework seeks to promote a more consistent approach to injury prevention and health management, with the potential for universal validation through collaboration with experts worldwide.

**Supplementary Information:**

The online version contains supplementary material available at 10.1007/s40279-025-02276-5.

## Key Points

**Table:** 

Implementing injury and illness surveillance (IIS) guidelines across diverse sports settings poses challenges, requiring adaptations to fit specific contexts. This paper introduces a framework to standardize and optimize IIS implementation, addressing these challenges.
The findings highlight the critical role of centralized oversight, routine data collection, and stable staffing in ensuring consistent data quality in IIS systems.
While the integration of electronic data tools and real-time monitoring has significantly enhanced data accuracy and reporting, maintaining engagement across large regions and navigating regulatory variations remain ongoing challenges.

## Introduction

Injury and illness surveillance (IIS) systems play a crucial role in professional sports by monitoring athlete health, thereby informing measures to prevent injuries and illnesses, and potentially improving performance [1–3]. The current general consensus statement on IIS in sport was published by the International Olympic Committee in 2020 [1], followed by a football (soccer)-specific extension in 2023 [4]. These statements provide comprehensive guidelines on injury and illness definitions and data collection protocols to standardize outcomes across settings and sports. However, despite the theoretical robustness of these guidelines, their practical implementation remains challenging across diverse sports environments [4–9].

While the consensus statements meticulously outline *what* needs to be done for effective injury surveillance, guidance on *how* to apply the guidelines in real-world settings is needed. The gap between the fundamental principles and their real-world application can lead to variability in data quality and adherence to protocols, limiting the overall effectiveness of injury prevention strategies. One of the main challenges is that the implementation of guidelines requires customization to fit the specific context and structures of sports organizations [4–7], which can differ greatly in terms of resources, expertise, and culture [10]. There is little research sharing real-world experiences in developing and implementing IIS systems in sports [8, 11, 12]. The key challenge lies in translating the fundamental guidelines into practical, context-specific protocols that can be seamlessly integrated into different sports organizations. Drawing on experiences from multiple major leagues and tournaments, this paper reflects on the successes and challenges faced during the implementation of IIS systems across diverse football contexts. It aims to offer actionable recommendations for the future development of IIS frameworks that can be adapted to a wide range of sports.

## Implementing IIS Systems in Football—Our Experience

### The Qatar Stars League (QSL) IIS Program: Implementation Evolution

The QSL IIS Program represents one of the longest-standing national epidemiological studies of injury, illness, and exposure among professional football players in the region [2, 13–20]. Launched in the 2008–2009 season, the program tracks QSL first and second-league male players, providing year-round monitoring for players over 18 years old. For the first five seasons, the QSL IIS Program used paper-based data collection initiated by a physician-researcher external to the teams. In 2013, a dedicated program (the Aspetar Injury and Illness Prevention Program [ASPREV]) took over the data collection in close collaboration with team medical staff recruited and employed within the National Sports Medicine Program (NSMP) at Aspetar, where a team physician and physiotherapist(s) are assigned to each club. From 2013–2014 through the 2017–2018 season, data collection was encouraged by Aspetar senior management but remained voluntary. Participation became mandatory for all QSL medical teams from the 2018–2019 season, integrating surveillance tasks into their routine duties. The team physician, with support from the physiotherapist(s), acts as the primary liaison with ASPREV, overseeing data collection and ensuring their submission to the ASPREV team.

In 2015–2016, the program was upgraded with a surveillance manual (Appendix 1) and electronic data collection tools, including customized Excel files (Appendixes 2a and 2b). Initially, Excel was chosen for its widespread availability, low cost, and flexibility, allowing customization to accommodate football-specific data entry using drop-down menus and Orchard Sports Injury and Illness Classification (OSIICS) codes [21]. This approach provided a simple yet effective solution in environments where more advanced infrastructure or internet access may be limited. Compared with alternatives such as REDCap and Microsoft Forms, Excel offered greater offline functionality, which was essential in early phases. REDCap, while more secure and scalable, requires a web server and specialized IT support, which were not feasible at program initiation. Microsoft Forms offers good usability but lacks advanced data structuring and longitudinal tracking features essential for ongoing surveillance. As the program expanded, considerations for transitioning to more scalable platforms such as REDCap have been revisited, especially in regions with better technological infrastructure and data security requirements. Over time, the tool has been refined to improve user experience, data accuracy, and reporting. To ensure data security and integrity, all Excel files were encrypted and stored on institutional servers with restricted access, ensuring that only authorized personnel could retrieve or modify the information. Data encryption was applied to safeguard sensitive records, preventing unauthorized access. In addition, compliance with institutional and national data protection regulations was maintained to uphold data integrity and confidentiality throughout the study. Recently, with secured funding, we developed a dedicated application specifically designed to streamline and enhance the data collection process.

We provided one-on-one training by two full time researchers for new participants to ensure proper tool usage and protocol understanding. The researchers oversee program implementation: one recruits teams and follows up on data submissions, while the other organizes training, monitors data quality, and provides monthly status reports. Team physicians submit data monthly to limit recall bias. Submitted data are reviewed, and amendment reports are provided for missing or inconsistent information. Recently, a more systematic approach to quality control has also been implemented, including monthly assessments of data completeness, accuracy, and adherence to definitions. In practice, submitted datasets undergo systematic quality control procedures. Surveillance officers review each monthly or tournament dataset for completeness, logic, and consistency. Automated checks within the Excel tool flag missing entries, illogical time-loss values, and code mismatches (e.g., injury mechanism inconsistent with diagnosis). If discrepancies or duplicate entries are detected, such as identical injuries logged on the same date, teams are contacted directly to verify and correct the data. Each dataset is then reviewed a second time before integration into the central database. In addition, a standardized amendment report is shared with the team for any required corrections.

At the end of each season, teams receive a comprehensive report comparing their injury and illness data to league benchmarks. This report (Appendix 3) has evolved on the basis of team feedback and needs. The report generation process is automated, using the PowerBI software and the data collected throughout the season. Reports are shared digitally through email with the team’s medical doctors. To meet the specific needs of each team or region, the reports are customizable, allowing for tailored recommendations and insights based on the team’s injury patterns and regional factors. This customization ensures that the reports are practical and directly applicable to each team’s unique circumstances, enhancing the overall impact of the data provided.

**Fig. 1:**
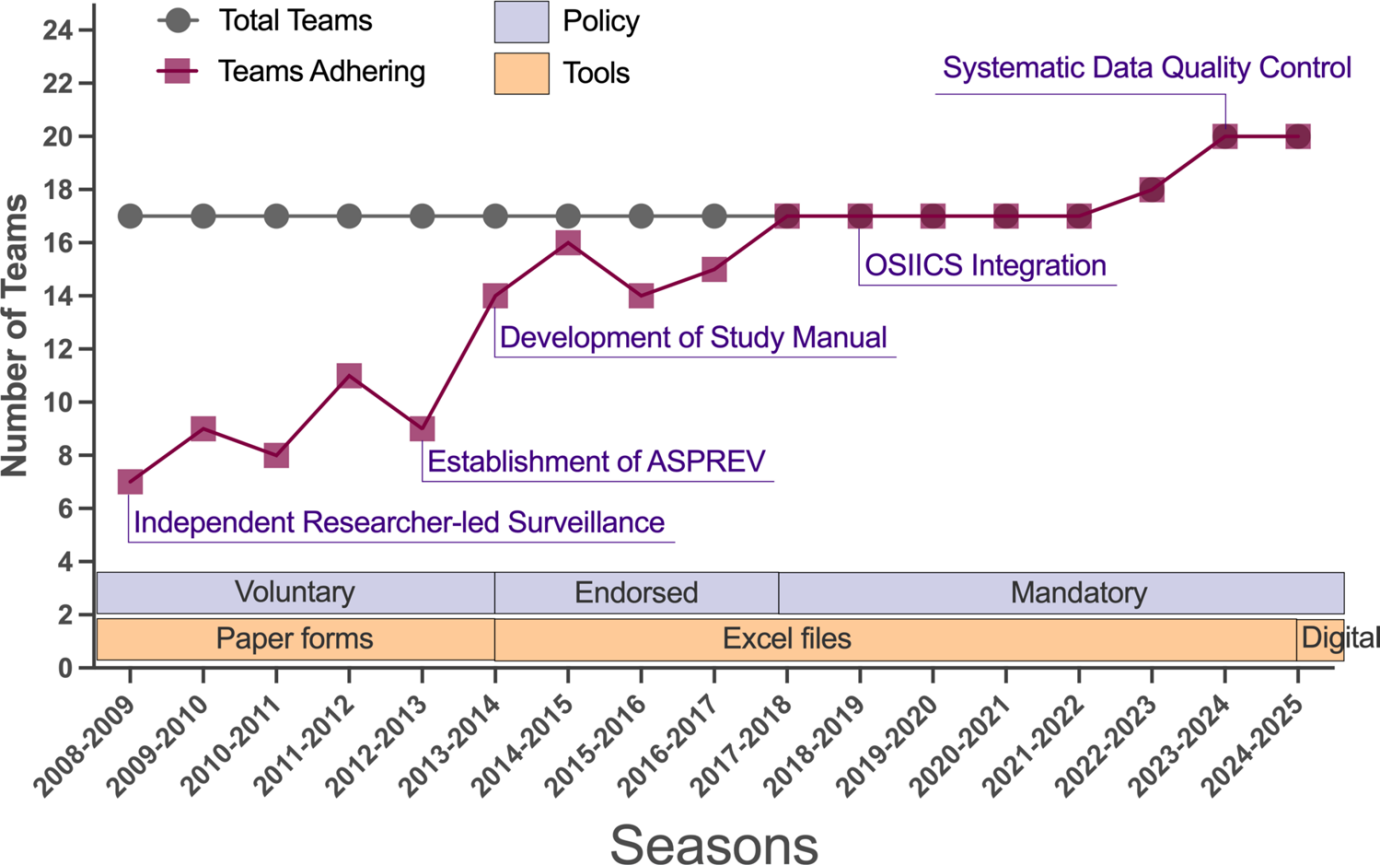
Evolution of the Qatar Stars League injury and illness surveillance implementation over 17 seasons. *OSIICS* Orchard Sports Injury and Illness Classification, *ASPREV* Aspetar Sports Injury and Illness Prevention Program

The adherence of teams to the QSL IIS Program has improved substantially since its establishment (Fig. 1). In the 2008–2009 season, only 7 out of 17 teams participated in the program. Over the following seasons, there was a steady and progressive increase in team engagement. By the 2017–2018 season, adherence had reached 100% and has been maintained since (Fig. 1). It is important to note that the development of the QSL IIS Program did not follow a traditional pilot-testing model. Instead, it emerged from an individual initiative in the early years, with data initially collected by an external physician-researcher. These early stages functioned as a de facto pilot, revealing many of the practical challenges of surveillance implementation in real-world settings. Lessons learned during this period, particularly prior to the establishment of ASPREV, guided the more formalized rollout of the surveillance system. Since then, iterative refinement has been ongoing. End-users, including team physicians and physiotherapists, regularly provided feedback through direct communication, monthly data submissions, and annual ASPREV workshops. These channels have been instrumental in identifying user needs, refining data collection tools (such as improving the Excel sheet’s usability), and optimizing reporting formats on the basis of practical utility.

### Asian Football Confederation (AFC) IIS: From National Success to Continental Challenge

Building on the success of the QSL IIS Program, we aimed to expand the program to a broader, continental scale. The AFC Elite Club IIS Program (AFC IIS) was launched officially in January 2017 [22]. The AFC Champions League, one of the premier club football tournaments in Asia, involves teams from across the continent competing in a structured, multi-stage tournament. It is organized by the Asian Football Confederation (AFC), with the tournament typically consisting of a group stage followed by knockout rounds leading to the final. A workshop at the AFC annual club manager meeting was held, which the team physicians were also invited to attend. The program targeted adult male players in AFC leagues. Participation was voluntary and teams interested in participating were requested to provide participation consent, after which they received the surveillance manual (Appendix 4), the Excel data tool (Appendix 5), and an online training session for the nominated club contact person (usually the team physician or physiotherapist).

While the core elements of the AFC IIS remained consistent with the QSL IIS, slight modifications were made to cater to the specific needs of the teams participating in the AFC leagues. For instance, de-identified Excel files were used for data collection to ensure confidentiality. Each year, the program is presented at the AFC annual meeting, inviting and encouraging new qualifying teams to join the program. Similar to the QSL program, each team benefits from a detailed annual report that provides insights into their injury and illness patterns, benchmarked against the average across the participating AFC teams.

**Fig. 2:**
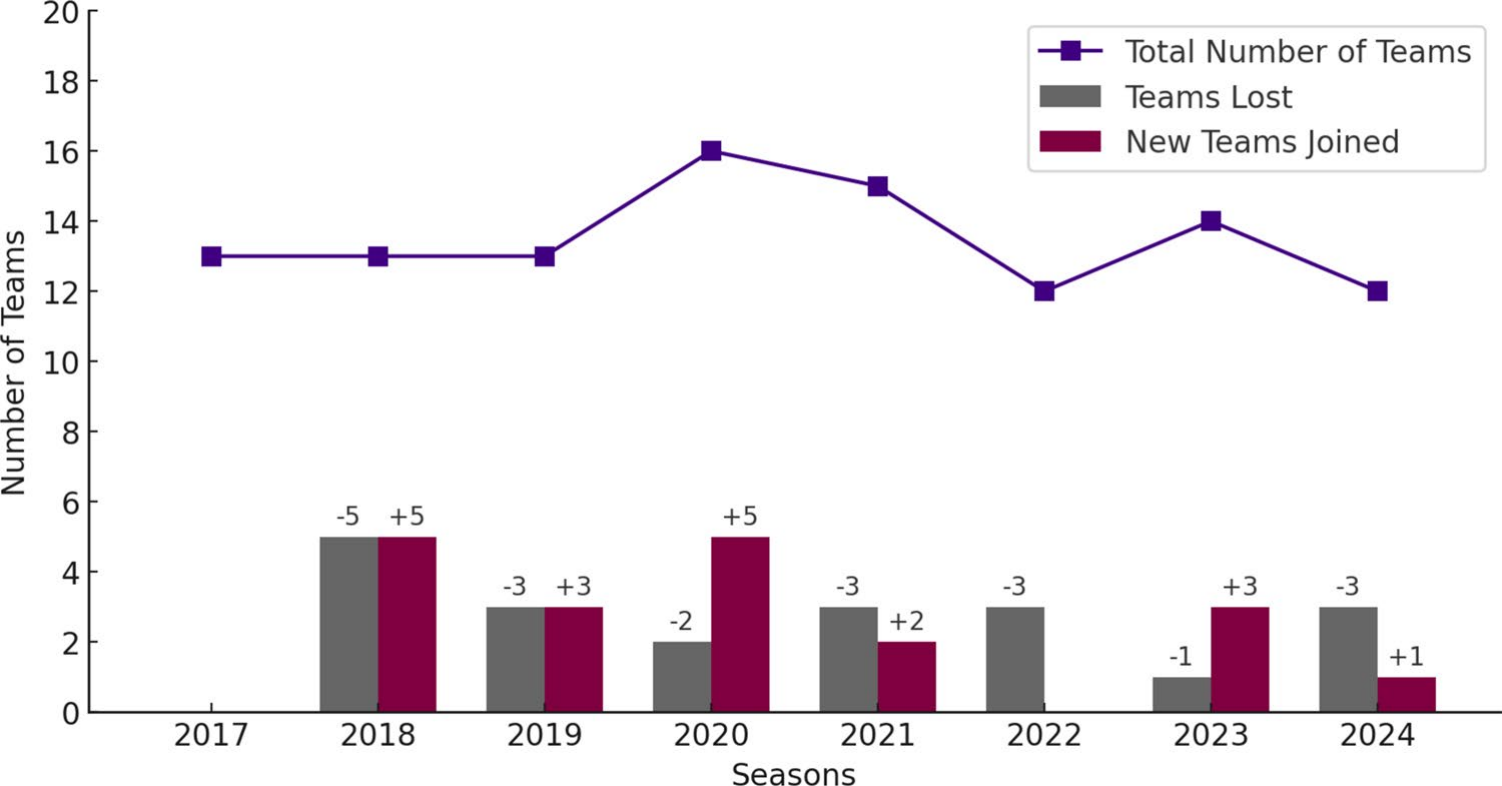
Team continuity and changes in the Asian Football Confederation (AFC) league across eight seasons

A total of 13 different countries have been represented to date (UAE, Qatar, Japan, Iran, Australia, China PR, Thailand, Hong Kong, the Philippines, Malaysia, India, Saudi Arabia, and Bangladesh), reflecting a broad and diverse participation from across Asia. From 2017 to the 2024 seasons, 23 clubs have taken part in the program, with an average participation span of four consecutive seasons per club. Notably, seven clubs demonstrated strong continuity, participating in the program for seven to eight seasons. In contrast, five clubs participated in only a single season (Fig. 2).

### From Leagues to Tournaments: National Team Surveillance

Implementing IIS in national team tournaments introduces a distinct set of challenges compared with regular league settings. Tournaments, such as the Fédération Internationale de Football Association (FIFA) World Cup or AFC Cup, are characterized by shorter timeframes, temporary team compositions, and intense scheduling demands. These factors require a different approach to the more stable, long-term structures seen in league environments. Addressing these requires tailored strategies. We began by organizing pre-tournament sessions for team physicians and physiotherapists, within 1–2 months prior to the FIFA World Cup Qatar 2022 [23] and a number of AFC tournaments. These sessions introduced the IIS protocols, with tools and procedures explained. Afterward, an official communication with tools and a comprehensive surveillance manual (Appendix 6) was sent to participating teams. A follow-up meeting with team medical staff was held after team arrival in Qatar to address any concerns and review data requirements.

Each team designated an on-site contact, i.e., team physician or physiotherapist, to submit all required forms: demographics, exposure, injury, illness, and consent. Data collection began with the first official training session and continued through the last match.

During the FIFA World Cup 2022, a surveillance officer assigned by FIFA supported teams in data reporting, ensuring submission before each match. If required, FIFA venue medical officers at each stadium were also requested to collect forms that had not been handed to the surveillance officers from the team contact person. Data collection was done using paper forms submitted in sealed envelopes to ensure confidentiality.

For AFC tournaments, data collection is an integral part of the routine medical procedures mandated by the AFC Sports Medicine unit through AFC regulations. Data collection is mandatory and consent must be signed by all players before the start of the tournament. Data were collected either on paper or editable PDF forms. Teams could submit forms via email or hand-deliver them in sealed envelopes to surveillance officers or venue medical officers. The results were then uploaded to a cloud-based server, allowing easy access, review, and follow-up on missing data.

**Fig. 3:**
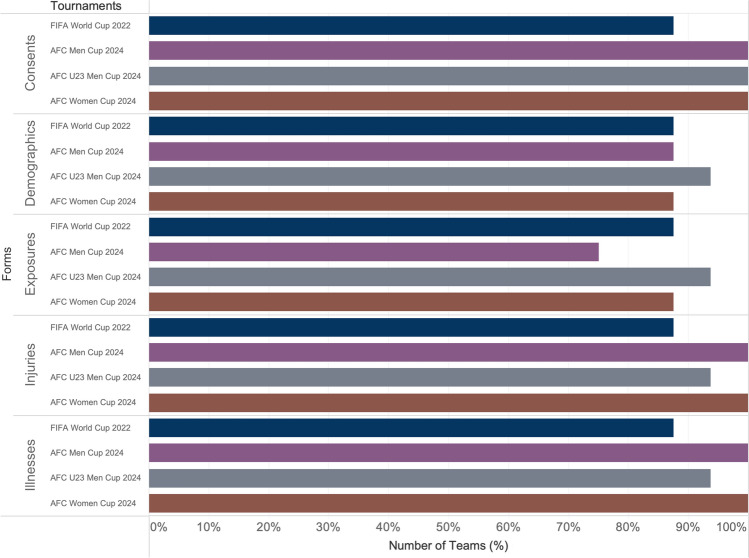
Form submission completeness across the injury and illness surveillance (IIS) tournaments. *AFC* Asian Football Confederation

IIS has been implemented across several major tournaments, including all participating teams in the FIFA World Cup 2022 (32 teams), AFC Men’s Cup 2024 (24 teams), AFC U23 Men’s Cup 2024 (16 teams), and AFC U17 Women’s Cup 2024 (8 teams). Data collection was managed by a dedicated team of surveillance officers, with five officers overseeing the FIFA World Cup, three officers assigned to the AFC Men’s Cup, and two officers each managing the AFC U23 Men’s Cup and AFC Women’s Cup. Figure 3 illustrates the percentage of teams from each tournament that successfully submitted the required forms.

While IIS implementation at both league and tournament levels follows similar core principles, their effectiveness varies due to structural and logistical differences. In league settings such as the QSL, the presence of consistent medical staff, stable reporting routines, and centralized oversight enables sustained, high-quality data collection. In contrast, tournament surveillance presents unique challenges: team compositions are temporary, staff turnover is high, and data must be collected in real time under tight timelines. These factors can compromise consistency and completeness. However, the presence of dedicated surveillance officers during major tournaments has been a key enabler in maintaining high data quality, compensating for the transitory nature of tournament teams. Furthermore, while leagues benefit from longitudinal data that support long-term injury prevention strategies, tournaments provide critical short-term insights, especially into acute injury patterns and match-related illnesses. Both settings offer valuable data, but their surveillance effectiveness depends heavily on context-specific adaptations and support structures.

## Developing a Framework for Effective Implementation of IIS Systems: Lessons, Challenges, and Strategies

Our experience in implementing IIS programs across multiple professional football settings has revealed key barriers to and facilitators of successful implementation. The insights gained from these experiences have guided the development of a step-by-step framework tailored for football organizations aiming to adopt or enhance their IIS practices (Table 1). In the following, we describe what we believe to be key the requirements for effective implementation of IIS programs: surveillance manuals, team engagement, surveillance officers, and organizational support.

**Table 1:** Injury and illness surveillance framework: a step-by-step guide to implementing injury and illness surveillance (IIS) in professional football

**1. Pre-implementation planning**
**Objective:** Establish a strong foundation for the IIS program by clearly defining goals, securing resources, and aligning stakeholders
**Define objectives**: Clearly outline the specific goals of the IIS program (e.g., reducing injury rates, improving player health, enhancing player and team performance)
**Stakeholder engagement**: Actively engage key stakeholders, including medical staff, technical staff, and team managers, by aligning the program objectives with their needs and priorities. Involve governing bodies (e.g., FIFA, AFC, UEFA, MAs, FAs) in the process to ensure that stakeholders’ aims are addressed, thereby fostering genuine commitment and enhancing buy-in. This collaborative approach helps build a shared sense of purpose and ensures that the program is mutually beneficial, increasing the likelihood of successful implementation
**Resource allocation**: Ensure that necessary resources, such as trained personnel, data collection tools, and IT infrastructure, are available and appropriately allocated
**Ethics approval**: Ensure the program complies with ethical guidelines established by relevant institutions and organizations and obtain necessary ethics approvals, ensuring the program is conducted in a manner that respects the rights of all participants
**Consent**: Ensure a plan is in place for obtaining informed consent from all participants. Secure informed consent from all participants before data collection, using either paper forms or digital methods
**2. Team structure and responsibilities**
**Objective:** Create a well-defined team structure with clear roles and responsibilities to facilitate effective data collection and communication
**Injury surveillance group:**
**Team/club**: Each team/club needs to nominate the contact person in charge (e.g., team physician) who will oversee data collection and reporting
**IIS group**: A surveillance officer(s) needs to be assigned to specific teams/clubs to facilitate communication. The number of IIS officers required should be decided on the basis of the number of teams/clubs involved and resources available
**Organization**: Establish support structures within the organizing body, and including external consultants if necessary
**3. Surveillance processes**
**Objective:** Implement a standardized and systematic process for injury and illness surveillance that can be applied consistently in the relevant setting
**Initiation**:
Conduct at least two meetings with the injury surveillance group: the first 1–2 months prior and the second within a week before the tournament/league starts
Prioritize group meetings with all group members and conduct individual meetings where required
Prioritize in-person meetings but conduct online meetings when necessary, depending on the situation and available resources
**Data collection monitoring**:
Implement continuous monitoring of data reporting from teams/clubs, with exposure data collected daily covering training sessions and matches and injury/illness data reported immediately as they occur and the final diagnosis has been reached
Allow no more than 1 week of delay in sending the monthly data
Adapt the daily monitoring process for tournaments, ensuring real-time data submission from all participating teams as required
Allow no more than 3 days of delay in registration in a tournament setting
**Data collection tools**:
Utilize standardized electronic tools, apps, or customized spreadsheets to ensure consistency across teams
Provide an IIS manual to streamline and standardize the process
Ensure familiarization of all the group members with the process and tools during the initial meetings and provide comprehensive training on using collection tools to minimize errors and ensure data completeness
**4. Quality control and data management**
**Objective:** Maintain the accuracy, completeness, and security of collected data through systematic quality control measures and secure data management practices
**Systematic data quality control**:
Implement regular assessments to ensure data accuracy and completeness (ranging from daily checks during tournaments to weekly/monthly checks in league settings)
Establish a feedback loop to promptly identify and correct any data discrepancies as they arise
**Data security and confidentiality**:
Use secure, de-identified systems to store and manage data, ensuring compliance with relevant ethical standards and data protection regulations
**5. Reporting and feedback**
**Objective:** Provide actionable insights to teams through detailed reporting and continuous feedback, fostering improvement in injury prevention strategies
**End-of-season reports**:
Generate detailed reports for each team at least every season, comparing their current data with previous seasons and league benchmarks and providing tailored recommendations for improvement
The report includes (but is not limited to) injury/illness incidences, burdens, location, type, mechanism and situation, onset, time loss, player availability, and exposure scores
**Continuous improvement**:
Establish a mechanism for gathering feedback from stakeholders to refine the surveillance process continuously
Adapt the surveillance framework based on advancements in sports medicine and evolving needs
**6. Training and education**
**Objective:** Ensure that all personnel involved in the IIS program are thoroughly trained and regularly updated on best practices
**Initial and ongoing training**:
Conduct comprehensive training sessions for all new staff involved in the surveillance program
Provide ongoing education to keep all stakeholders informed of updates and changes in the surveillance system
**Educational materials**:
Develop and distribute manuals, guidelines, scientific resources, and other materials to support the education of team members

*IIS* injury and illness surveillance, *FIFA* Fédération Internationale de Football Association, *AFC* Asian Football Confederation, *UEFA* Union of European Football Associations, *MAs* member associations, *FAs* football associations

### The Role of Surveillance Manuals in Guiding Implementation

The surveillance manuals for the QSL and AFC IIS programs were developed prior to the release of the general  International Olympic Committee (IOC) consensus statements [1] and were based on the Fuller et al. statement on football [3]. We incorporated key definitions and elements, adapting them to the specific contexts of the QSL and AFC IIS programs [3].

While this statement [3] provides clear instructions on the principles, we identified the need for adaptations to better address the nuances of injuries in our context. For example, linking gradual onset injuries to specific events such as matches or training sessions was often challenging. We refined our approach by allowing physicians to report injuries identified during matches or training, providing more comprehensive data even when the exact timeline was unclear. These considerations, among others, were later formalized in the IOC consensus statement [1], as well as in the updated, football-specific extension [4].

Unlike the consensus statements, which outline *what* should be done, our surveillance manuals provide detailed, step-by-step instructions on *how*. They include comprehensive explanations and visual aids, such as spreadsheet figures, for completing the exposure and injury/illness data. Practical tools such as spreadsheet templates with built-in formulas and data entry guidance simplify data collection and analysis, and help ensure accuracy and consistency. The manual also emphasizes continuous improvement and feedback, encouraging users to suggest changes, keeping the program relevant and effective.

### Enhancing Team Engagement

The QSL IIS Program saw a significant increase in adherence, rising from fewer than half of the teams in the 2008–2009 season to all teams by the 2017–2018 season. Several factors contributed to this improvement, including regular communication with teams about the project’s importance, the development of a comprehensive surveillance manual, the provision of annual reports to the teams, securing management support to make participation mandatory, and the implementation of electronic data collection tools. In addition, elements such as regular educational initiatives, face-to-face meetings, and ongoing direct follow-up played a crucial role. Rather than any single factor, it was the combination of these efforts that led to the observed success.

The program’s value extends beyond compliance: teams have expressed that the detailed annual reports are particularly beneficial for developing their risk management plans for injury prevention. These reports provide actionable insights into injury trends and benchmarks, enabling teams to tailor prevention strategies to their specific needs. By reducing injury incidence and severity, teams have also been able to lower the overall costs associated with injury treatment and recovery, highlighting the program’s practical benefits for both player health and financial management.

Comparing our results with UEFA’s well-established injury surveillance system [24], the UEFA Elite Club Injury Study, provides valuable context. As documented by Ekstrand et al. [24], the UEFA system also benefits from robust medical infrastructures and consistent adherence across European clubs. The QSL IIS Program benefits from a unique advantage: the medical staff across all clubs are part of one medical organization/employer and not directly paid by their club. This centralized system ensures that medical professionals are not only highly trained but also experience minimal turnover, with a majority of the medical staff having served the same clubs for over 10 years. This stability is a significant factor in the successful implementation and continuity of the IIS in Qatar. Indeed, the success of the QSL IIS Program demonstrates the importance of centralized and stable medical staffing, which facilitates the consistent application of surveillance protocols. Strong personal relationships and trust built with team medical staff over the years have also been crucial. These connections have been fostered through regular workshops and annual meetings where medical staff, including fitness coaches, gather to exchange experiences and discuss ways to improve injury and illness risk management. Such gatherings create valuable opportunities for networking, sharing solutions to common challenges, and continuously enhancing the surveillance framework. Similarly, the UEFA Elite Club Injury Study group also credits the annual meetings of all UEFA participating teams, which offer valuable opportunities for networking with peers in similar roles and for addressing common challenges. We believe that such regular meetings help to strengthen personal connections and provide a platform for sharing solutions to problems faced across teams. However, we recognize that in some settings, there is limited oversight of sport governing bodies, or these bodies operate in a decentralized manner, and adopting a centralized approach may not be feasible. In such cases, we recommend that organizations focus on fostering strong collaboration between medical teams, coaches, and sport governing bodies. This can be achieved through regular communication, data sharing, and developing tailored injury and illness surveillance protocols that fit the specific needs of each organization, even in the absence of centralized oversight.

Similarly, the AFC IIS Program’s broad participation highlights its success in engaging a diverse group of teams across Asia. However, the fact that five clubs participated for only a single season indicates challenges in maintaining long-term engagement. These drop-outs were primarily due to changes in team staff, which led to a loss of contact with the clubs. The vast geographic scale of Asia also makes it difficult to maintain consistent communication, especially through virtual meetings across different time zones.

These experiences highlight the need for robust, continuous communication channels and regular support to sustain engagement. To address these challenges, developing strategies to anticipate staff changes—such as preparing handover procedures and ensuring new staff are onboarded seamlessly—could help improve long-term participation and data consistency in the future. These challenges are reminiscent of the variability seen in the National Collegiate Athletic Association (NCAA) injury surveillance program in the USA, where adherence is often dependent on individual institutions rather than a centralized governing body [25]. The high turnover of medical staff in the AFC program, as in the NCAA system, significantly impacts data consistency and quality. This comparison underscores the importance of centralized oversight and continuous support in sustaining participation and ensuring reliable data collection over time.

The QSL’s approach, reflecting the successes of UEFA’s system, underscores the importance of centralized oversight, mandatory participation, and stable medical staffing. In contrast, we suggest that the AFC’s experience, akin to the challenges faced by the NCAA, highlights the need for tailored strategies. Some strategies which appear to have been successful include providing additional support and training for new medical staff to ensure continuity and consistency in data collection across different organizational contexts. The QSL and AFC IIS programs are key case studies in effective injury surveillance system implementation, showing that with appropriate strategies and structures, high participation and adherence can be achieved. These experiences suggest that other sports leagues and organizations should adopt a centralized approach, mandate participation, and provide ongoing training and support for medical staff, especially in high turnover environments. Such measures can ensure the long-term success of surveillance systems, leading to more effective injury prevention and management in professional sports.

### The Key Role of Surveillance Officers

The implementation of IIS across major tournaments, including the FIFA World Cup 2022 [23], AFC Men’s Cup 2024, AFC U23 Men’s Cup 2024, and AFC U17 Women’s Cup 2024, marks a significant step forward in systematic large-scale data collection and athlete health monitoring. While surveillance has been conducted at major football tournaments for years, these recent efforts have introduced new methodologies and technologies that enhance the quality and scope of data collected. For instance, the use of standardized electronic tools, real-time data submission protocols, and dedicated surveillance officers have improved data accuracy, consistency, and timeliness compared with earlier approaches.

Moreover, these tournaments have expanded the scope of surveillance to include a wider range of health outcomes, such as illness monitoring alongside injury data. The integration of comprehensive training for medical teams and the establishment of mandatory data collection protocols at the AFC tournaments have further ensured uniform data collection standards across participating teams. This systematic approach represents a significant evolution from earlier practices, which often lacked the same level of standardization, quality control, and real-time monitoring capabilities.

The participation of multiple teams in each tournament was facilitated by dedicated surveillance officers, who were crucial in managing and ensuring the successful collection of health data, securing consistency and reliability. These officers oversaw form submissions, supported medical teams, and ensured adherence to established protocols. This approach effectively mitigated common data collection challenges, such as discrepancies, incomplete submissions, and variability in reporting standards.

This strategy was compared with other large-scale sports events, such as the Olympic Games [26, 27] and the Pan American Games [11, 28], where similar roles were assigned to ensure data quality. It becomes clear that the success of these programs hinges on effective coordination and the continuous support provided to participating teams. Thus, considering a dedicated surveillance officer to address the similar challenges ensures more consistent data collection despite the diversity of the teams involved. During the Pan American Games [11, 28], for example, variability in staff experience and turnover was identified as a significant challenge, impacting the consistency and quality of the data collected.

### Mandatory Versus Non-mandatory Participation

A key distinction between the AFC tournaments and the FIFA World Cup lies in their approaches to data collection. In the AFC tournaments, data collection is an integral part of the routine medical procedures mandated by the AFC sports medicine unit as a legal requirement for participation. Data collection consent is mandatory for all players participating in the AFC tournaments, meaning that they are required to sign as part of the overall data collection process, which includes surveillance and doping control. This approach ensures that comprehensive data are collected consistently from all teams. However, when using the data for research purposes, additional steps are required. The data must be anonymized to protect players’ identities, and ethics approval must be obtained before any analysis or publication. This anonymization requirement limits the depth of analysis because it prevents linking the data to individual players’ information, such as medical history or video footage of incidents. In contrast, the FIFA World Cup operates under a research-focused model, with ethics approval obtained before the tournament. Players provide consent for their data use, potentially allowing non-anonymized data to be linked to other data such as video footage of the incident, enabling more detailed analysis. This approach, while offering deeper insights into injury patterns, necessitates more rigorous consent procedures and ethical oversight to protect player rights and privacy. The difference between these mandatory and nonmandatory approaches highlights different priorities, with the AFC focusing on comprehensive coverage and uniformity, and FIFA enabling detailed, individualized analyses.

### The Proposed Framework Solution: Addressing Inconsistencies and Promoting Adaptability

On the basis of our experience with IIS programs, we have developed a standardized framework that offers a logical, step-by-step guide for effectively implementing IIS systems (Table 1). It is intended to tackle a key challenge in injury surveillance: the inconsistent implementation of guidelines across different sports and settings. We suggest it as a standard for developing and implementing surveillance programs, emphasizing the need for both standardization and flexibility.

By adjusting data collection methods and surveillance tools to fit specific needs, the framework can enhance monitoring and prevention across diverse athletic settings. Furthermore, its principles may be relevant not only at the elite or professional level but also in youth, amateur, and developmental sports.

### Strengths and Limitations of the Framework

While the framework (Table 1) provides a robust approach, it has some limitations. The findings are primarily based on football, particularly within the QSL and AFC Elite Clubs, which may limit their external validity to other sports with different organizational structures, resources, and cultural contexts. The focus on professional football may also hinder full applicability to amateur or youth sports with limited medical staff and organizational support. While this framework reflects our direct implementation experience within centralized and well-supported football environments, we recognize that contextual differences may influence its broader applicability. Therefore, we propose this framework as a practical starting point to initiate a global collaboration. As a next step, we aim to convene a panel of international experts including those from decentralized and resource-limited settings to refine, adapt, and validate the framework across diverse sporting and governance contexts. To elevate the framework to a broader, internationally validated level, we plan to engage a diverse panel of global experts, including sport physicians, epidemiologists, researchers, and representatives from sport governing bodies with experience in injury and illness surveillance. While the methodology is currently under development, structured approaches such as a Delphi process are being considered to ensure a systematic and inclusive consensus-building effort. This approach aims to ensure that the framework remains robust and applicable to various sports and organizational contexts, while still building on the foundational principles established through our experiences. Variability in data quality may arise from differences in medical personnel training and team engagement, and ethical considerations vary across regions, complicating consistent adherence to guidelines. In addition, reliance on electronic tools, while improving efficiency, may pose challenges in settings with limited technology access. Despite these limitations, the framework may serve as a valuable tool that can be adapted to enhance IIS across sports and settings.

## Conclusions

This framework offers a practical and structured approach to implementing IIS in football, grounded in the experiences of the QSL and AFC Elite Clubs. While its current design reflects the specific contexts in which it was developed, it serves as a foundation for broader application. Future validation through international expert collaboration will be essential to refine the framework and ensure its adaptability across diverse sports, regions, and organizational models.

## Supplementary Information

Below is the link to the electronic supplementary material.

Appendix 1: QSL Surveillance Manual (DOCX 2955 KB)

Appendix 2a: QSL Exposure Excel files Prototype (XLSX 2372 KB)

Appendix 2b: QSL Injury and Illness Excel files Prototype (XLSX 308 KB)

Appendix 3: Dummy Report Example (PDF 12190 KB)

Appendix 4: AFC Surveillance Manual (PDF 1264 KB)

Appendix 5: AFC Excel File Prototype (XLSX 2444 KB)

Appendix 6: FIFA Surveillance Manual (PDF 1566 KB)

## Declarations

### Availability of Data and Material

Data are not available for confidentiality reasons. Materials are provided as appendices.
