# Supplementary material for: Lessons Learned from Implementing Injury and Illness Surveillance in Professional Football: Introducing a New Implementation Framework
Source: Sports Med. 2025 Jul 11;55(10):2375–85. doi: 10.1007/s40279-025-02276-5 (PMC12513881; doi:10.1007/s40279-025-02276-5)
Supplement: Supplementary file 1 — Appendix 1: QSL Surveillance Manual (DOCX 2955 KB) [file 40279_2025_2276_MOESM1_ESM.docx]

**Aspetar Injury & Illness Surveillance Program**

**FOOTBALL**

**National Sport Medicine Programme (NSMP)**


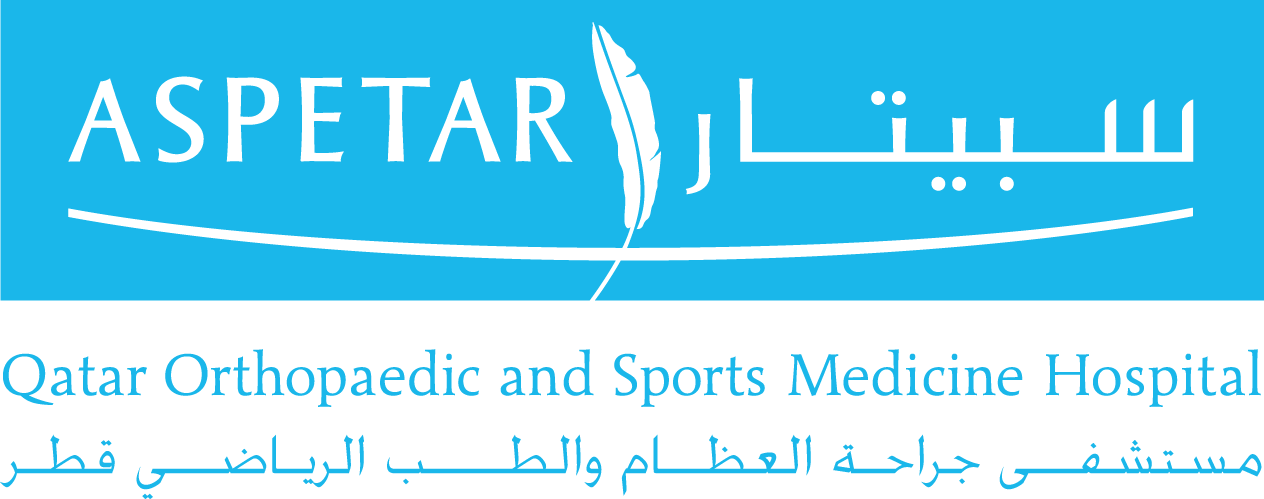


**Aspetar Sport Injury and Illness**

**Prevention Program (ASPREV)**

*June 2018 Version*

**MANUAL**

**Aspetar Surveillance Program**

**(Injuries – Illnesses)**

**2017 - 2018 Team Sports Guidelines**

**Contact persons (Program Group)**

**Dr Montassar Tabben**

29222 Aspetar

Sport City

Doha (Qatar)

Tel +974 4413 2757

Fax + 974 4413 2020

[karim.chamari@aspetar.com](mailto:karim.chamari@aspetar.com)

&

**Mrs. Rima Tabanji**

29222 Aspetar

Sport City

Doha, Qatar

Tel +974 44132570

Fax + 974 4413 2020

[rima.tabanji@aspetar.com](mailto:rima.tabanji@aspetar.com)

**Contents Page**

**1. Definitions 4**

- 1. **Training exposure**
  2. **Match exposure**
  3. **Injury**
  4. **Illness**
  5. **Rehabilitation**
  6. **Date of Injury**
  7. **Date of Return to Full participation**
  8. **Re-injury**
  9. **Exacerbation**
  10. **Onset of injury**
  11. **Injury occurring during Match or Training**
  12. **Menses in Females**

**2. Who is responsible and which players should be included? 8**

**2.1 Who should be the club’s contact person?**

**2.2 How many players from each club should be included in the**

**surveillance program?**

**2.3 What about changes in the first team squad?**

**3. How to fill in the “Exposure file” 9**

**3.1 – How to introduce Players in the list.**

**3.2.1 – Session Exposure**

**3.2.2 – Players’ Exposure**

**3.2.3 What if the coach decides that a player should not participate**

**in a training session?**

**4. How to fill-in the “Injury Card’’ or ‘’Illness Card” form 15**

**4.1 Specific injury cards**

**4.2 Groin Card**

**5. Reports Sheets 20**

**Description and content**

**6. Links Between XL Files 22**

**7 How to use the “Filter option’’ in Excel 24**

**6. Sending the forms to Aspetar (Study Group) 27**

**6.1 How to send the Files?**

**6.2 When to send the forms?**

**6.3 Confidentiality**

**6.4 Questions**

**Introduction**

The prevention of injuries and illnesses relies on a **sound Risk Management Plan** for each athlete/team of athletes. The first step in developing a Risk Management Plan consists of identifying the injury/illness risks for a given sport/group of athletes.

Once the Risk has been identified, then prevention programs can be developed targeting the most important problems in terms of frequency and severity.

Therefore, ASPREV, with the approval of NSMP management, has developed the “Aspetar Injury and Illness Surveillance Program” to identify specific risks to Qatar Clubs and Federations.

To be able to draw meaningful conclusions from the results of this surveillance program, it is vital that all participating Clubs and Federations collect data in a uniform way. This program follows international definitions and methodology used by e.g. FIFA and IOC (Fuller et al, 2006, Engebretsen et al. 2010) and Aspetar/Asprev/NSMP guidelines.

It is recommended that any Medical staff involved with this program reads this booklet. These Guidelines set the basis of the Surveillance Program by setting important definitions and data collection procedures.

We hope you will enjoy reading it and we would be happy to receive your feed-back for its’ continuous improvement.

**1. Definitions**

To be able to draw meaningful conclusions from the results of this surveillance program, it is vital that all participating clubs collect data in a uniform fashion. This program follows international definitions and methodology (Fuller et al, 2006) and NSMP/Aspetar guidelines. The sport practiced by the player is named: ‘’Main Sport’’. This applies for Football, or Handball etc…

- 1. **Training exposure**

***Training exposure is defined as any team-based or individual physical activities under the control or guidance of the team’s coaching and/or fitness staff that are aimed at maintaining or improving players’ skills or physical condition/recovery.***

Examples:

1. recovery session with water training after a match is considered as a training session
2. Pre-match warm-up and post-match cool-down sessions should be recorded as ‘’Training’’ exposure. If ever an injury occurs in these pre- or post-match sessions, please record it as a ‘’Training’’ injury.
   1. **Match exposure**

***Match exposure is defined as play between teams from different clubs.***

Note: this includes match exposure for the first team players who also sometimes play for teams other than the first team, for example the club’s reserve team, or national teams.

Examples: a “Training match” between “yellow” and “green” players (of the same team) is considered as training and NOT ‘’Match activity’’.

Friendly games played against ‘’another Club’’, (usually with the presence of an official referee) are considered as ‘’Match activity’’

- 1. **Injury**

A ***‘’Time-loss’’*** *definition* of Team Sport injury is used:

***An injury is any physical damage that occurs during Training or Match and results in the player being unable to fully participate in one or more training or match-play sessions.***

- Injuries that do not cause absence from Sport activities do not count.

- Injuries that occur outside Main Sport activities (for footballers: Football and training for football performance) do not count. PLEASE DON’T RECORD THEM. If they cause absence from sport, please complete the form with “Absent for other reasons” () (see XL section further)

These are injuries having required ‘’Medical Care’’, but as long as they did occur during training/competition for the main sport of the player, they do not have to be recorded in the files. The Doctor will thus keep the ‘’injury cards’’ separately from the other injuries.

- Every injury that causes absence (even only for the last part of a training session) should be recorded. Any slight injury (for instance, skin abrasion that is been treated for few minutes on the side of the pitch with the player resuming training/play), does not count).

- 1. **Illness**

“Time-loss” illnesses are collected.

A “Time-loss” definition of Illness is:

“A physical or psychological complaint or manifestation experienced by an athlete that occurs at any time (during or out of Main Sport activities) and which causes absence from Main Sport activities”

This means that:

ANY illness or complaint that occurs at any time – whether it is during or outside main Sport activities, AND WHICH CAUSES absence from Main Sport activities: COUNTS

For Vaccination related illnesses that cause absence from training: It is important to specify which vaccine the illness may have been precipitated by (If known). Please mention this in the Diagnosis section of the card.

PLEASE NOTE:

- For the URTI (Upper Respiratory Tract Infection) in the ‘’Affected Systems’’ section, please provide the details of the affected areas: [Throat, Ears, and/or ‘’Nose-Sinuses’’] in the “**Diagnosis” section**. For any Illness affecting the ears that is NOT an URTI, please specify what it is in ‘’Other’’ and also provide more details in the ‘Diagnosis’’ section.
- For LRTI (Lower Respiratory Tract Infection), please provide the details of the affected areas: [Trachea, Bronchi, Lungs] in the Diagnosis section.
  1. **Rehabilitation**

**The “Rehabilitation period”, is the full period comprised between the first day of activity resumption on the field/clinics (after the full stop due to injury/illness), to the last session of rehabilitation/training before ‘’Return to full participation’’**

A player is classed as injured or ill for as long as he cannot participate in any type of training. A player is considered fully rehabilitated when the team doctor/physiotherapist declares him fit for full participation to training AND matches.

**Note**: if any part of a training session is modified for a player due to an existing injury this is considered part of the rehabilitation programme for that player and should not be considered as training.

**1.6    Date of Injury/Illness**

**The date of injury/illness is set when the athlete stops his/her Sport (exp: Football) activity** (training OR matches) **because of this injury/illness** (NOT WHEN THE SYMPTOMS STARTED and INDEPENDENTLY OF THE DATE OF CONSULTATION with the medical staff).

- 1. **Date of Return to Full participation**

**This is the date corresponding to the first day of “full/unrestricted” participation to the Sport activity (training or matches).**

The choice of the coach to use the player or not for any game has no influence on this date.

**Note:** the information of this date has to be entered in the XL file in order to fully complete the injury/illness Card (whenever no date of “Return to full participation” is entered, the column with “Special attention” will stay “Red” as a reminder to enter this date to close the Card).

Note that this date cannot be the same as the day of injury/illness (otherwise there will not be at least one day of time-loss).

- 1. **Re-injury**

***‘’Re-injury’’ is defined as an injury of the same type and at the same site which occurs after a player’s ‘’Return to full participation’’ from the previous injury.***

Please record as a ‘’re-injury’’ all injuries in the same anatomical location **within 1 year**.

**Note:** When recording the re-injury period you have options ranging from within ‘’one week’’ to within ‘’12 months’’. *A ‘’re-injury’’ has to be recorded as a NEW injury case/Card with the previous ‘’original injury’’ file closed with a clear ‘’Return to full participation’’ date.*

- 1. **Exacerbation**

**An Exacerbation occurs when time-loss from training or matches occurs to a player who has not been yet in ‘’full participation’’ (Still in Rehabilitation)**.

The injury shall be classified as an Exacerbation where the ‘’original injury’’ worsens to the point where the player has to stop his rehabilitation/training activity for at least one session.

*Note: Obviously, in contrast, if the player has been completely symptom-free and fully fit to participate from a previous injury (full training + Matches), the injury shall be recorded as a ‘’Re-injury’’ and not as ‘’Exacerbation’’ (see section 1.8).*

*Specific example: the player had an injury and is regularly performing his rehabilitation. The medical staff is happy with the evolution and decides to test the player in full training before clearing him for a ‘Date of return to full participation’’ (full training + competition). If an injury occurs at that moment of ‘’testing’’ it has to be classified as exacerbation, since this specific training was a testing and not a regular training occurring after Date of return to full participation’’.*

For any ‘’Exacerbation injury’’, a NEW file/Card (and date of injury) has to be set, with the previous ‘’original injury’’ file NOT yet closed by a ‘’Return to full participation date’’. The only exception lies when a player joins the club with an ‘’already existing complain’’. In this case, the Doctor does not have the ‘’original injury file’’, and thus any eventual ‘’exacerbation’’ will not be preceded by an ‘’original injury’’ file.

- 1. **- Onset of injury**

Injuries should be classified as ‘Sudden’’ or ‘’Gradual’’, according to their onset. These terms replace the previous terminology used, where injuries were classified as ‘’Traumatic’’ or ‘’Overuse’’.

If the injury is resulting from a specific incident clearly recalled by the athlete, it should be classified as ‘’Sudden Onset’’. If the injury developed progressively over time (days/weeks/months, it should be classified as ‘’Gradual Onset’’.

Thus, a Gradual onset injury is when the player **CANNOT** recall a specific moment in which she/he became injured.

- 1. **- Injury occurring during Match or Training**

Please mention if the injury occurred during ‘’Match’’ or ‘’Training’’ and also mention the corresponding timing of the injury (at which moment it occurred during the corresponding match or training session).

**N/A (Not Applicable)** applies to the cases of ‘’Gradual Onset’’ injuries, where it is not possible to state if the injury occurred during Match or Training.

- 1. **– Menses in females**

For any absence because of painful menses, the Doctor will have to open a new file of Illness, simply putting ‘’Menses’’ in the diagnosis.

**2. Who is responsible and which players should be included?**

**2.1 Who should be the club’s contact person?**

The contact person will be responsible for collecting the data and delivering them to the study group. We suggest the Team Doctor (or Head Physiotherapist if there is no doctor available) as contact person.

**2.2 How many players from each club should be included in the surveillance program?**

All players in the first team squad should be included in the study. We suggest a number of around 25 players per team being managed during the season. However, the Excel files allows for managing much more players. For instance, during the preparation phase, the number of players can widely exceed 30 players. All the injury/illness and exposure of these players should be noted. The Exposure files allows entering 50 players. Therefore, any player being involved with the club for more than few days (testing days, for example), should be monitored.

**2.3 What about changes in the first team squad?**

a) All players who are in the first team squad should be included.

b) Players who join the team during the season should be included from their date of joining.

c) Players who leave the club during the season are excluded from the date of leaving the club. Players who are ‘’tested at the beginning of the season’’ shall also be included (as long as the testing period is not of only very few sessions. Players being tested for several weeks or months should be included, even if they did not obtain an Aspetar-MRN number).

d) Main ‘’Radif/Youngsters’’ players who continuously train with the first team, but sometimes go and play with the second/Reserve/U23 team, shall be included in the squad. When not with the First team, they should be noted as ‘’Absent’’ (for other reasons than injured/illness).

**Note:** if a player is injured and then leaves the club (or the end of season off-period occurs), then the contact person should fill an injury card for that injury and *estimate the time to return to play*.

**3. How to fill in the “Exposure file”**

**Important !**

***Please note that the files are ‘’Simple Excel (XL)’’ files, with many cells containing formulae that allow the file to function.***

*For instance, in the figure-1, the cursor has been placed on: Cell E-4 containing the formula shown in fx: =IF(A4="";"";A4&", "&B4&". "&C4)).* ***Please do not press DELETE when your cursor is on any Cell containing a formula.***

*Also, please do not go to fx and modify it. This would either introduce an error in the file or impede the function to work.*

Microsoft Office Excel versions prior to 2010 are not advised for use, due to functionalities issues.

**
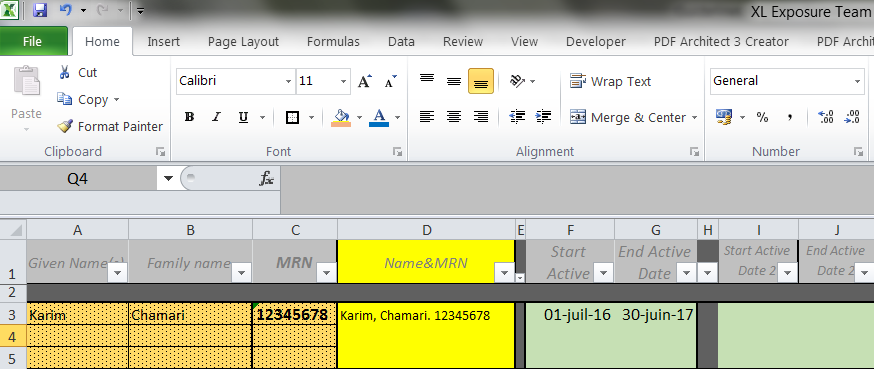
**

Figure 1: ‘’Exposure Sheet’’ file. Sheet ‘’Players-List’’.

**3.1 – How to introduce Players in the list.**

Please Enter only A, B, C information (Given name(s), Family Name, and MRN). **The D cell will automatically be generated.**

When doing so, please enter first the players who are in contract with the club. Thereafter enter the players who are in the Reserve Team. Lastly enter the players who are ‘’under testing’’ or at risk of leaving the group.

This will allow having the most active players on the top of the list. For now, it is not possible to re-organise the order of the list of the players during the season. If you start collecting the data with a list of players, the list will stay in the same order. You can only add players to that list.

You can enter up-to 50 players (allowing entering all the players that come to train with the team at any moment of the season).

MRN are to be entered for players having an MRN. For players not obtaining this number, please leave the cell empty. Please note that each player going through the screening at Aspetar, automatically obtains an MRN number (8 numbers digit).

**Activity Dates:**

These dates are set on the XL of the exposure sheet to facilitate the collection of the data.

**When you enter a player, the automatic dates of ‘’Activity’’ will be set as ‘’1^st^ July to end of June of next year’’. Please change the dates accordingly to the reality of the activity of each player**.


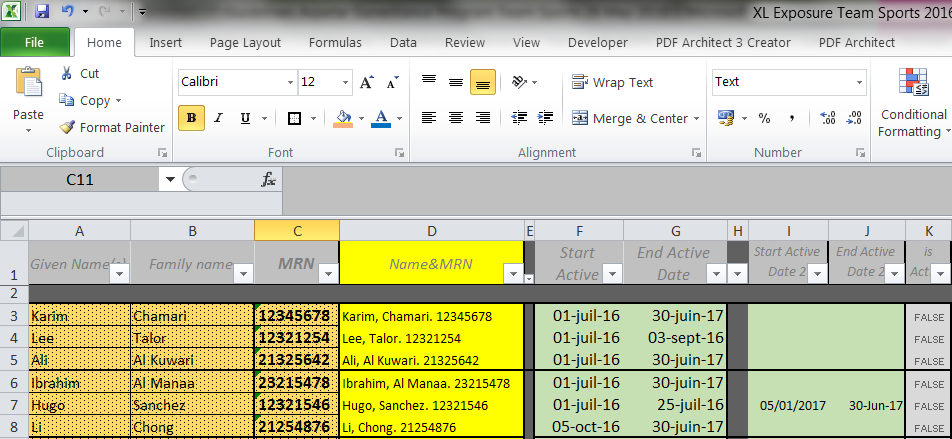


Fig 2: Example of Activity dates

For instance, on fig 2, Player in Raw8 joined the group late (5^th^ of October).

Player Raw7, trained with the team for few weeks and then left (25^th^ of July). He came to join the team again from the 5^th^ of January 2017 to the end of the season.

Player Raw4 left the club on the 3^rd^ of September.

The XL file allows to have 1 to a maximum of 2 ‘’Activity’’ periods per player. This will allow the File to automatically declare the player as ‘’Present’’ for any session within these ‘’Activity’’ period. For any period not included, the file will NOT consider the player within the team, and then will not set any exposure for him. The concerned cell will display ‘’inactive’’, not allowing to enter any exposure (for instance, see fig 3 for the player Ali Ben Abid (having left the club on the 3^rd^ of July).

**The ‘’Activity’’ periods are for important changes of status (player leaving the club or joining the Club) and NOT for absences for injuries** (even if very long – please see below).

It is practical to use these ‘’Activity Dates’’ for players. This will avoid having an ‘’inactive’’ player (player having left the club, for instance), being allocated a training or match exposure, for every session. This will force the used to go to his cell and remove the exposure. These are unnecessary actions that are boring for the user. By declaring the player ‘’inactive’’ the XL automatically sets the exposure as ‘’inactive’’ and the user does not have to care about all ‘’inactive players’’.

**Basic information about the players:**

On the right of the ‘’Players’ List’’ sheet in the ‘’Exposure XL’’ file please enter basic information about the players (Age in years; Body mass (weight) in kg; Body height in cm) and player’s main position and dominant limb. (Fig 2A, right section)


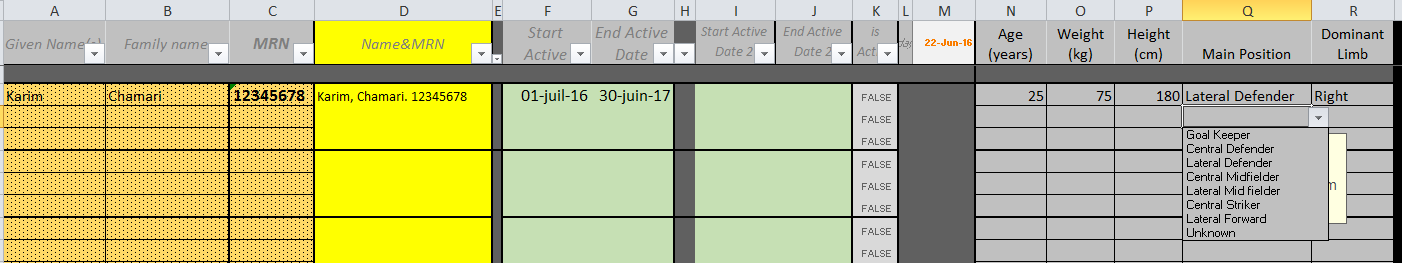


Fig 2A: Example General information entry for a player.

**Player’s Main Position:**

This corresponds to the player’ preferred position. If the coach uses the player in another position for tactical reasons, this does not have to impact on this option. Usually the player knows his ‘’preferred position’’ and normally it is the position in which he plays the majority of the matches.

For instance, in football you will be able to choose between the following options:

- Goalkeeper / Central defender / Lateral defender / Central midfielder / Lateral midfielder / Central striker / Lateral striker

**Dominant limb:**

Simply ask the player which is his dominant limb, i.e. the limb with which he predominantly shoots and passes the ball. If the player says he is ambidextrous, please ask him the following question: ‘’If you are about to shoot a decisive penalty kick in an important official game, with which limb would you shoot the ball?’’. The answer should provide the information about his dominant limb.

Players’ basic information should be collected at the beginning of the season (or as soon as the player joins the team). If for instance, body mass changes during the season, please do not enter the new body mass in this basic information section.

**3.2 Managing the Sessions’ ‘’Attendance/Exposure’’**

The ‘’Exposure Sheet’’ contains 12 sheets corresponding to 1 sheet per month. Each sheet is called by a number. ‘’1’’ for the first month of the season. **Please do not change the name of the sheet (leave it as 1)**, as the injuries/illnesses reports do rely on the reference of each sheet by its’ name (1) to calculate the report scores.

Each sheet contains as many columns as the number of days in the month.

For each day, there are 5 possibilities of sessions to be entered (1 match, 2 training sessions with the ball and 2 training sessions without the ball, see fig. 3).


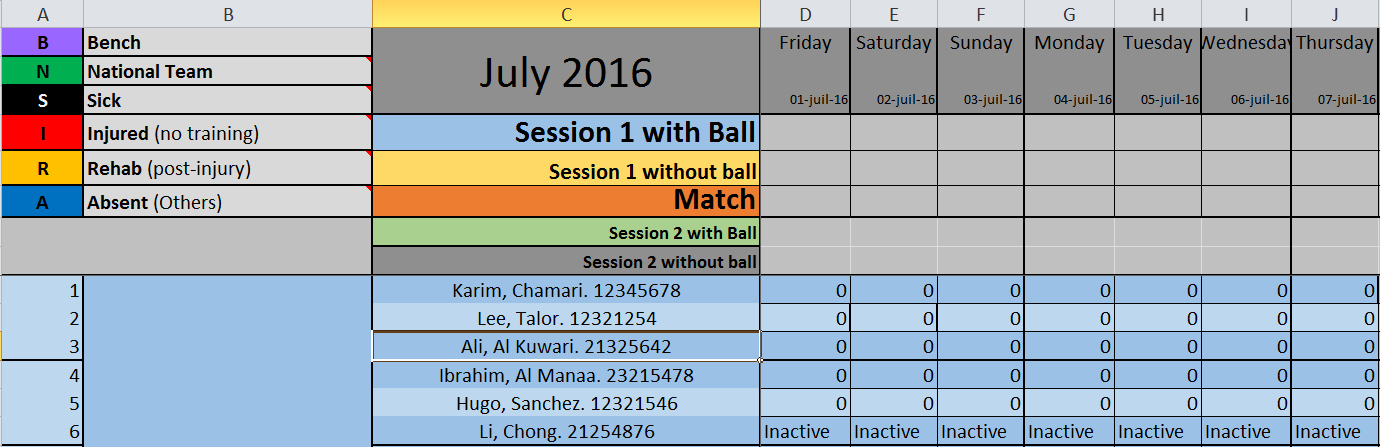


Fig 3: Monthly ‘’Exposure/attendance sheet’’.

**3.2.1 – Session Exposure**

If your team performed only one session in a day, please enter the ‘’Exposure/Duration’’ (in minutes) in the appropriate cell.

For instance, if for the 1^st^ of July the team trained for 98 min, enter ‘’98’’ in the cell D4. If the team played a game for 95 min, please enter this exposure in cell D6 (Figure 4).


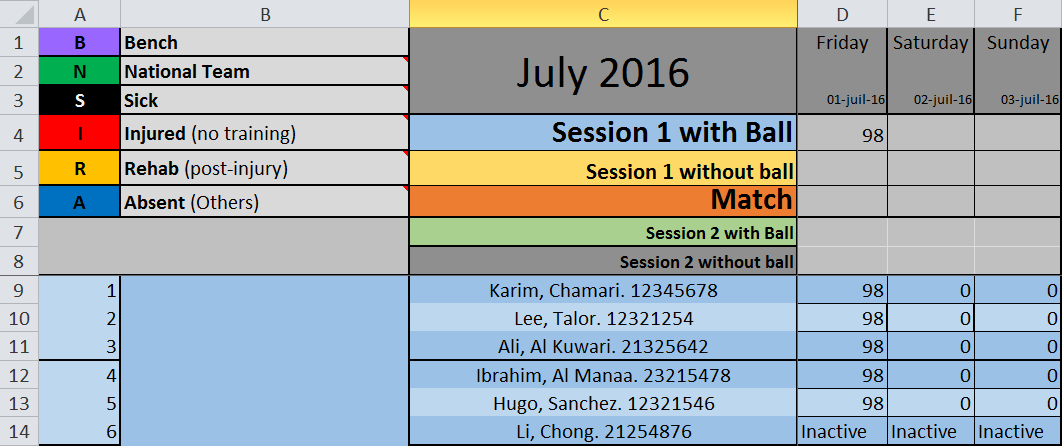


**Figure 4:** example of exposure entry

For any exposure entered, for all the ‘’active’’ players, the entered exposure will be allocated in the TABLE corresponding to the type of session chosen (Figure 4).

**There are 5 tables superimposed** in different colours.

Blue:‘’Session 1 with the ball’’,

Yellow: ‘’Session 1 without the ball’’

Orange: ”Match”

Green:‘’Session 2 with the ball’’,

Gray: ‘’Session 2 without the ball’’

If you wish to enter multiple sessions please do as follows:

Enter the exposure of all sessions and then go and manage the ‘’group/players’’ in the corresponding Table.

For instance, if in the same day, your **First team played a Match** and a **subgroup of other players had 2 sessions** (one with the ball and one without the ball). Enter the durations of each session in the lines 4, 5, and - and then Go to each table to manage individual players’ exposure. This can also apply for example for 2 Training sessions done by the first team, while some players of the squad are involved in a ‘’Reserve team’’ Game.

Please note that:

**Session WITH the ball** corresponds to a session completely dedicated to technical/tactical aspects OR to sessions performed **mainly with the ball** in which there might be some ‘’physical exercises’’**. These sessions are the ‘majority’ of Main Sport team training sessions and are mainly guided by the Coaching staff (technical).**

**Sessions WITHOUT the ball** corresponds to sessions exclusively dedicated to physical training OR recovery (absence of the ball) and are usually guided by Fitness coach(es)/Physios.

**Important**: As most of the training sessions in the Main Sport are done at least partially with the ball, **then most of the sessions you will enter during the season are ‘’Sessions WITH the ball’’.** Again, even if a part of the session contains exercises without the ball, this session has to be classified as ‘’With the Ball’’.

**3.2.2 – Players’ Exposure**

When the ‘’Session’’ duration is set, all ‘’active’’ players are automatically set as present and the duration of the session is allocated to each one of them (Figure 4).

For non-active players, the cell will display ‘’inactive’’ (example: player having left the club at Mercato (players’ transfer window), all the rest of the season cells for him will be displayed as ‘’inactive’’, see Figure 3 as example).

For each session, please adapt for those player not having performed the part or theentire session. Thus, for any ‘’Training session’’ you will have to correct manually the cell for the special cases (injuries, rehabilitation, absence…).

Specifically for matches, you will have to manage all the cases of

1. players not exposed at all
2. players having had ‘’partial exposure’’ (player substituted and their substitutes)
3. players on the bench and not utilized.

For Sports where the number of substitutions is unlimited (exp: handball), the **Match exposure** should be estimated by the staff (the technical staff can help in that regard). Please allocate each player one of the following 5 duration-zones:

1 - 0 min (player not involved at all) = Bench player, no exposure.

**2 - 25% of Competition duration** = Player having participated to >0 to 25% of time.

3 - **50% of Competition duration** = Player having participated to >25 to 50% of time.

4 - **75% of Competition duration** = Player having participated to >50 to 75% of time

5 - **100% of Competition duration** = Player having participated to >75 to 100% of time

Example: a game duration (excluding pauses at half-time but including the short time-outs) is of 1h40min (100 min). If a player is estimated having participated to 65% of the game duration, he will be allocated 75% of the time = 75 min.

HOW TO MANAGE SPECIAL CASES

**NO EXPOSURE**

**For any ‘’Absence or Non-Exposure’’ please:**

Go to the top-left of the Excel sheet and pick-up the ‘’Cause of absence’’ (fig 4A).


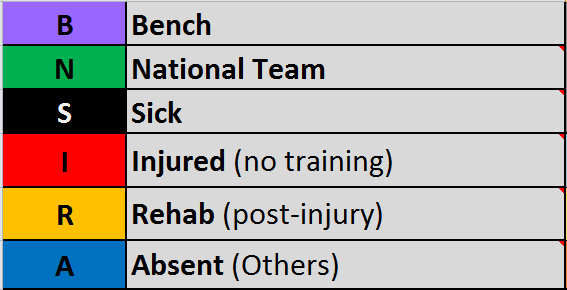


**Figure 4A**: examples of Types of absences

1. – Choose and copy the cell corresponding to the ‘’cause of absence’’
2. – Paste it on the cell corresponding to the ‘’absence session’’. (this will erase the allocated session duration, which will be replaced by a ‘’Letter/Color’’. – For instance: Black Cell with the Letter ‘’S’’ will be allocated to a sick player.

**PARTIAL EXPOSURE**

Any ‘’Partial Exposure’’ should also be changed manually.

Please first enter manually the exposure. Then colour the cell in yellow (optional) to be able to visually track the ‘’uncomplete exposures’’.

For instance the player 4 on figure 5 had only 22 min out of 98-min.


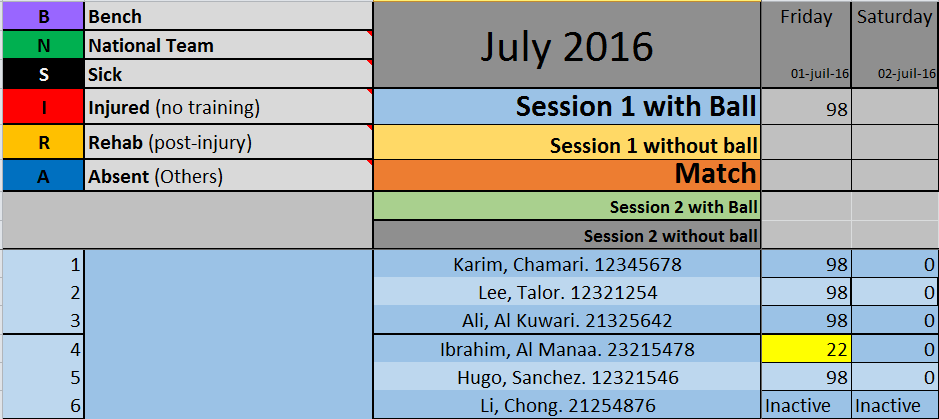


**Figure 5:** example of uncomplete exposure

How to insert a comment (optional)

*If you need to mark any comment on any cell you can put the ‘’cursor on the cell - then: Click right – and choose from menu: Insert comment’’. You will be able to add any comment you would like to have related to this particular cell. When you complete the entering of the text, the cell will display a little small triangle red mark in its upper-right corner. Later, to see your comment, just put the cursor on that cell without clicking. This will display your ‘’comment’’. By right-clicking again, you could, from the menu (i) delete your comment, or (ii) modify it.*

**3.2.3 What if the coach decides that a player should not participate in a training session?**

- If a player is recommended to rest due to an existing injury or so as not to aggravate an existing condition this should be marked as an injury on the attendance record (and obviously an injury card should be filled-in in the ‘’Injury File’’).
- If a player is recommended to rest as a safety precaution without any existing symptoms of any injury this should be marked as absence for ‘’Other reasons’’ on the attendance record (no injury card required).

**4. How to fill-in the “Injury Card’’ or ‘’Illness Card” form**

The Excel (XL) file contains 2 different spread-sheets for ‘’Injury’’ and ‘’Illness’’.

The File is set with some instructions on how to manage the information about injury/Illness. These appear when the cursor is placed on the corresponding cells (see fig 6).


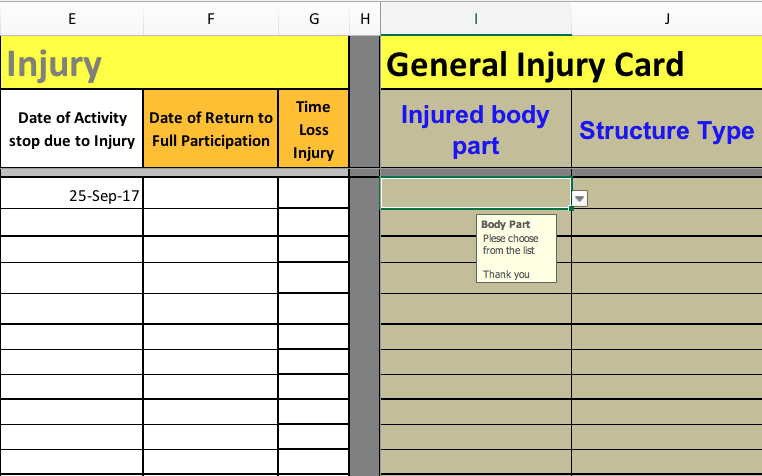


Fig 6: Pop-up ‘’instructions’’ that appear when the cursor is located on a cell.

Most of the Options are set as ‘’scrolling menus’’ from which the Doctor has to choose options (fig 6A). First step consists of choosing the injured body part.


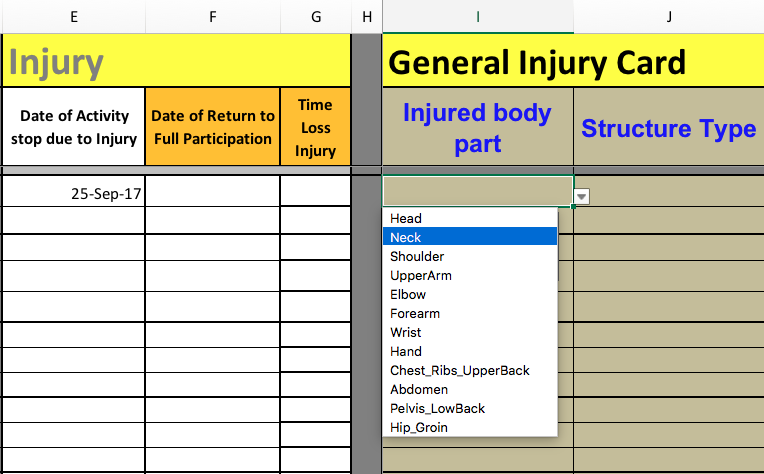


Fig 6A: Scrolling menus that appear when the cell is clicked.

Once it’s done the specific structure types of the chosen body part will appear as ‘’scrolling menus’’ from which the Doctor has to choose an option (fig 6B).


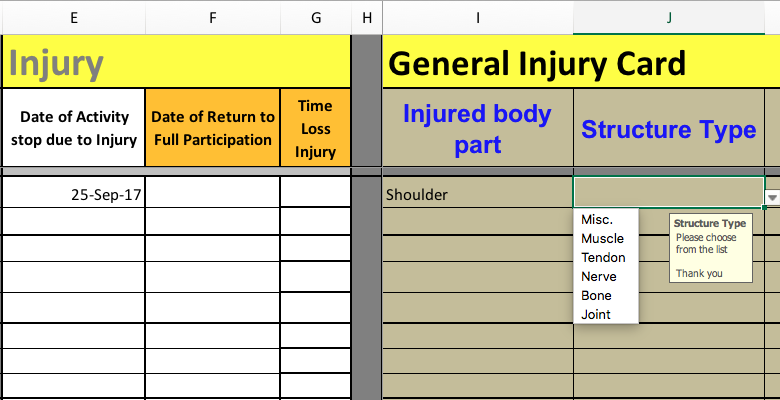


Fig 6B: Scrolling menu specific to the chosen body location: ‘’Structure Type’’, that appears when the cell is clicked.

The last two steps will allow to set the specific diagnosis, relative to the chosen body part and structure type. The proposed Diagnosis will appear as Scrolling menu to choose from (fig 6C).

.
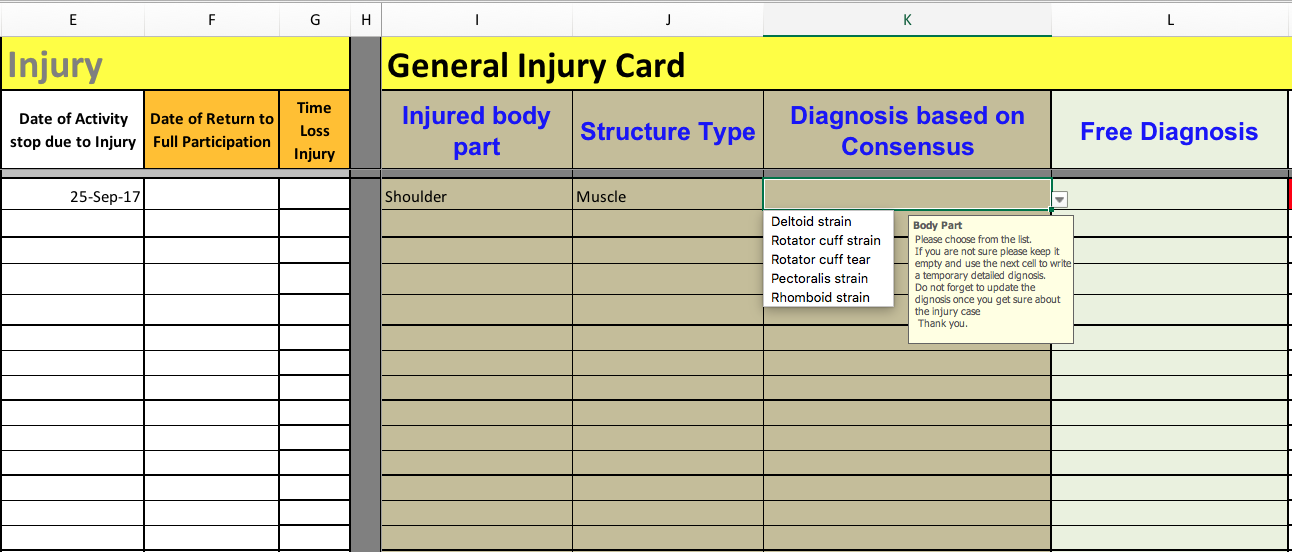


Fig 6C: Scrolling diagnosis menu relative to the chosen body location and structure type that appears when the cell is clicked.

‘’open cells’’ are set for adding text (fig 6D). when the diagnosis is not available or the Doctor is not sure yet about the diagnosis, for instance.


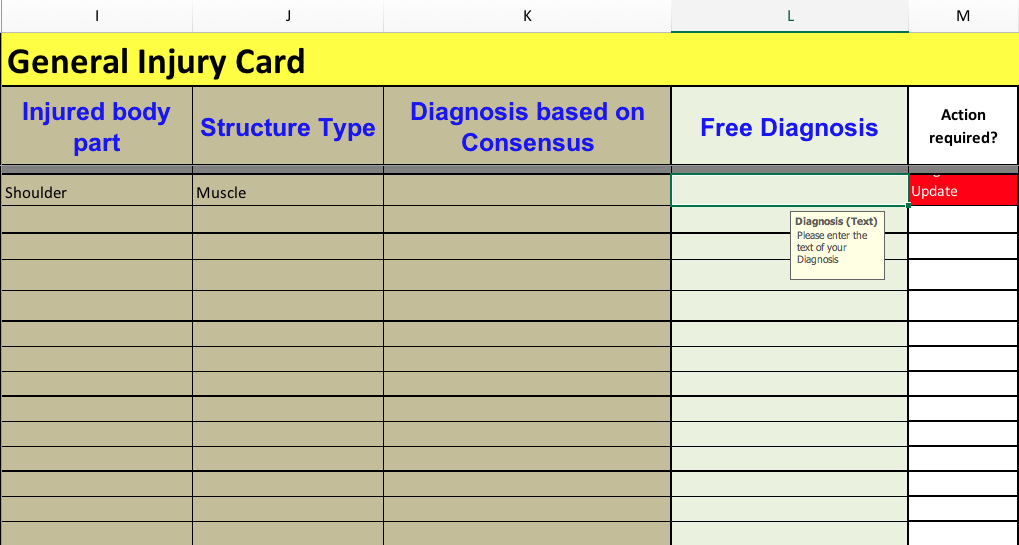


Fig 6D: Example of ‘’open text cell’’ where the user can enter a free text.

An injury/Illness card should be filled in for every TIME LOSS injury/Illness that occurs during the season (from the first to the last day of the season (season comprising the pre-season training period).

**All Fields prepared for Injury and Illness should be filled**.

In some cases, if it is not possible to choose from the menu, there is the ‘’I don’t know/Unknown/N-A’’ option to choose from.

In some scrolling menus, at the bottom, there is the option ‘’other’’. In case you tick this option, please fill in by ‘’free text’’ the next column headed: ‘’other’’.

Diagnosis: write the best diagnosis you have for the moment. If during the following days, you discover that the diagnosis was wrong, you can modify it. The important thing is that, when you send the data, you write the correct diagnosis (to your knowledge). Please utilize the UofC Sport Medicine Diagnostic Coding System (SMDCS), which has been recently adopted by Aspetar

Other comments: you could write any information which can be useful for you and for the better understanding of the injury/illness case.

**4.1 Specific injury cards**

For some of the more common injuries in football, specific injury cards were created.

1 - Anterior thigh

2 - Posterior thigh (Hamstrings)

3 - Ankle Sprain

4 - Groin

These ‘’Sections’’ in the XL file are located to the right of the ‘’General Injury Card’’.

Please fill-in any of these sections when appropriate.

**4.2 Groin Card**

The Groin injury card contains 4 slots for 4 possible ‘’Diagnosis’’. An athlete can have more than one entity, in which case multiple entities can be diagnosed. The NSMP staff should fill in first the most probable diagnosis (the “known” pain – Main entity), and then if there are more entities present, continue with the next more dominant pain, and so forth.

Please use the proposed **‘’Entities’’ (Fig. 7):**

**1 - Adductor-related groin pain:**

Adductor tenderness AND resisted adduction testing painful.

**2 - Iliopsoas- related groin pain**

Iliopsoas tenderness + more likely pain on resisted hip flexion AND / OR pain hip flexor stretching.

**3 - Inguinal-related groin pain**

Pain AND tenderness located in the inguinal canal region. No palpable inguinal hernia present. More likely if the pain is aggravated with resistance testing of the abdominal muscles OR on Valsalva/cough/sneeze.

**4 - Pubic-related groin pain**

Local tenderness of the pubic symphysis and the immediately adjacent bone. No particular resistance tests.

**5 - Hip-related groin pain**

Pain from the hip joint should always be considered as a possible cause of groin pain. Physical examination including passive range of motion and hip special tests - (Flexion-abduction-external rotation (FABER) and Flexion-adduction-internal rotation (FADIR) test should be performed in all cases. Tests are utilized for excluding hip-related groin pain when testing does not reproduce the athlete’s pain.

**6 - Other**

The main categories are orthopaedic, neurological, rheumatological, urological, gastro-intestinal, dermatological, oncological and surgical, but many other rare conditions could possibly cause pain in the groin region. This entity should be utilized when the complaints cannot easily be classified into one of the common defined entities.

**Important note:**

Please do not use one of the following terms: adductor and iliopsoas tendinitis or tendinopathy, athletic groin pain, athletic pubalgia, biomechanical groin overload, Gilmore’s groin, groin disruption, Hockey-goalie syndrome, Hockey groin, osteitis pubis, sports groin, sportsman’s groin, sports hernia, sportsman’s hernia.

**
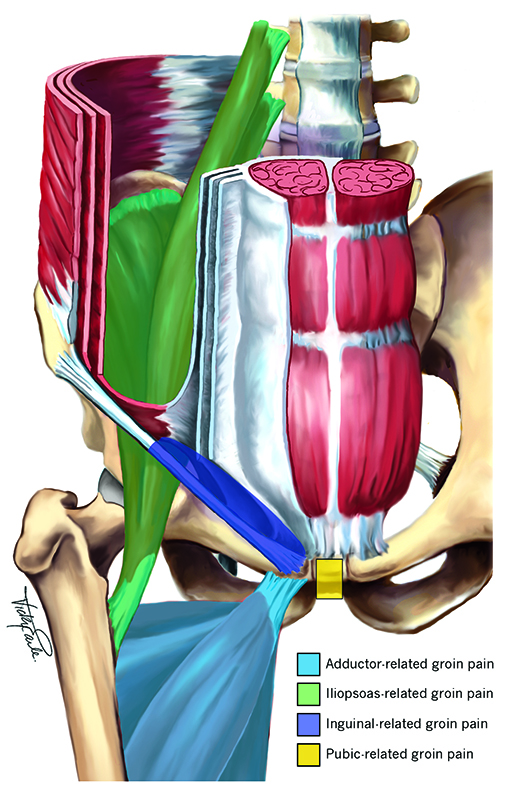
**

**Fig 7: Illustration of the Groin Entities.**

**5. Report Sheets:**

**The Reports tables allow having the REAL TIME OUTCOME of the surveillance monitoring job done by the Medical Staff.**

In each XL file there is one ‘’report sheet’’.

1 - In the Exposure file, the report (‘’Exp Summary’’ sheet) gathers the exposures of each month (with details of matches and specific training, see fig. 8). This sheet serves as basis of exposure for the injury and Illness rate calculation. The numbers appearing are the displayed in minutes (product of number of players per sessions’ duration).


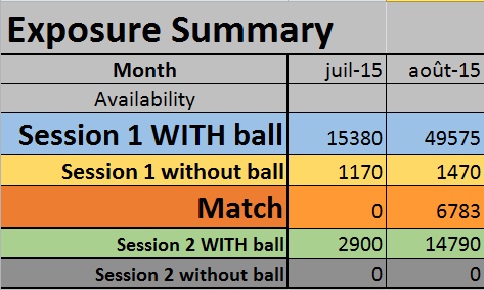


Fig. 8: Sample of Exposure Report.

2 - The Injury/Illness file contains also a report sheet named ‘’ Summary Inj-Ill’’ (see fig. 9).


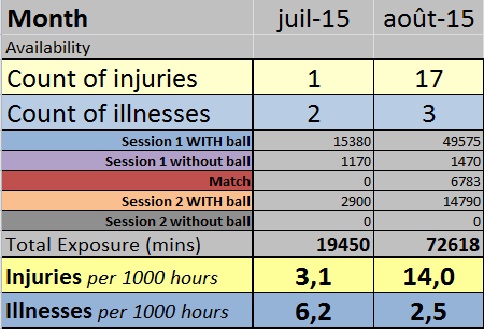


Fig. 9: Sample of Injuries/Illness Report.

For each month the number (count) of injuries and illnesses is displayed (upper part of the table). In the bottom part of the table, the injury and illness rates (n/1000 hours of exposure) are displayed.

At the bottom of the former table, there is another table summarizing the days lost for injuries or Illnesses per month. The availability (% of the players available for training/matches) is also set (see fig. 10). This data does not take into account absences for participation to national team camps/competitions.. For specific availabiliy including the national team activity, please refer to the summary sheet of the ‘’Exposure XL file’’.


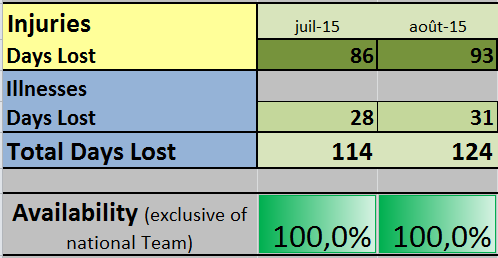


Fig. 10. Report of days lost and players’ availability.

**6. Links between XL files**

Two separate XL files have been created to allow the staff to manage 1) the Injuries/Illnesses and 2) Exposure in different files. Typically, the exposure file is used daily and could be managed by the Doctor, the Physio or the Fitness Coach. The Injuries/Illnesses file is usually managed by the Club Doctor.

When you receive the files by e-mail, the links between files are ‘’lost’’. You will have to re-set them by yourself according to the following procedure:

The XL files are linked to allow the ‘’Injury-Illness’’ file to use the data entered in the ‘’Exposure’’ file to calculate the injury and illness rates and also the players’ availability.

To allow this functionality to work, save both files on the same location on the same computer. Then, open both files.

The following procedure will have to be done for the first time when opening the files on the same computer

**1 – Step one**: open both files.

In the ‘’Exposure’’ File go to the ‘’Exp Summary’’ Sheet. Select the table from Cell B-4 to Cell M-8. (fig. 11), and then click-right: ‘’Copy’’.


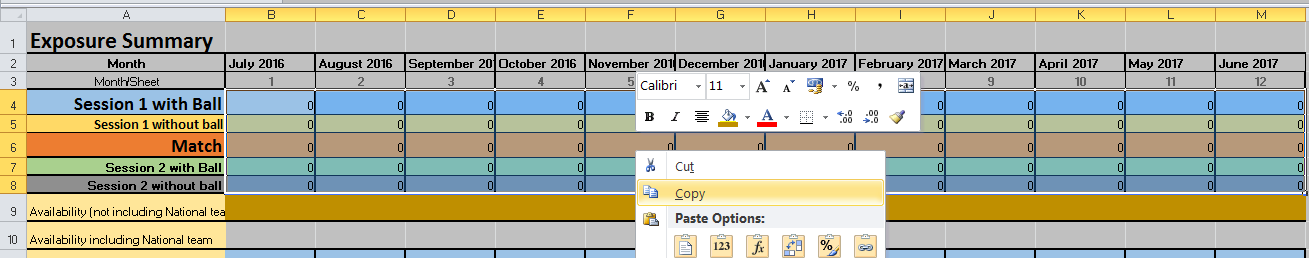


**Figure 11**: Selection of Exposure summary in ‘’Exposure File / Exp Summary sheet) to export to the ‘’Injuries-Illnesses’’ File.

**2 – Export this selection** to the ‘’Injuries-Illnesses’’ file as in figure 12.

Paste this selection in the ‘’Summary Inj-Ill’’ Sheet in Cell D4 (See figure 12).

Go to Cell D-4 as on the figure, click right and select ‘’Paste options’’ (far right: Paste link).


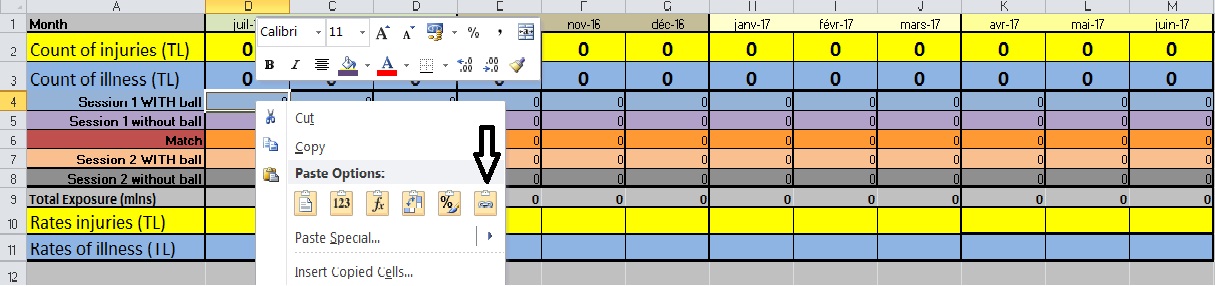


**Fig 12:** Pasting the selection in cell D-4 with special paste option ‘’Link’’.

Last step, export in the same way, from the same sheet of ‘’Exposure’’ file to the same sheet of ‘’Injuries-Illness’’ File the selection of ‘’Players’ Availability’’ in Row B9 to M9 (see figure 13).


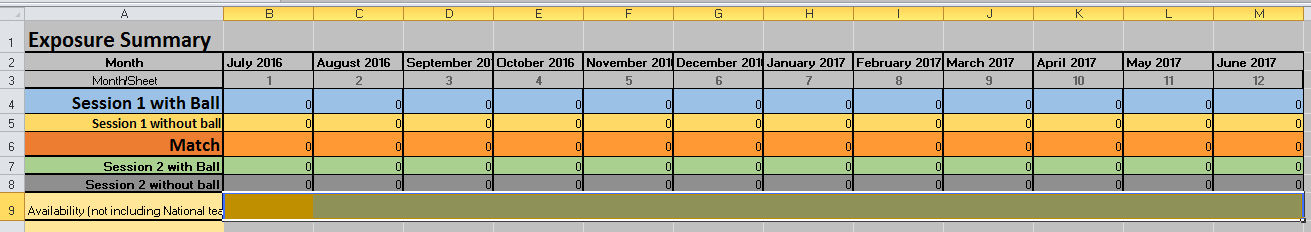


**Fig. 13:** Selection of Player’s availability from Exposure File (Exp Summary Sheet Row B9 to M9.

Export this selection to the ‘’injury-illness’’ file (sheet ‘’Summary Inj-Ill’’, Cell B-20) see figure 14.


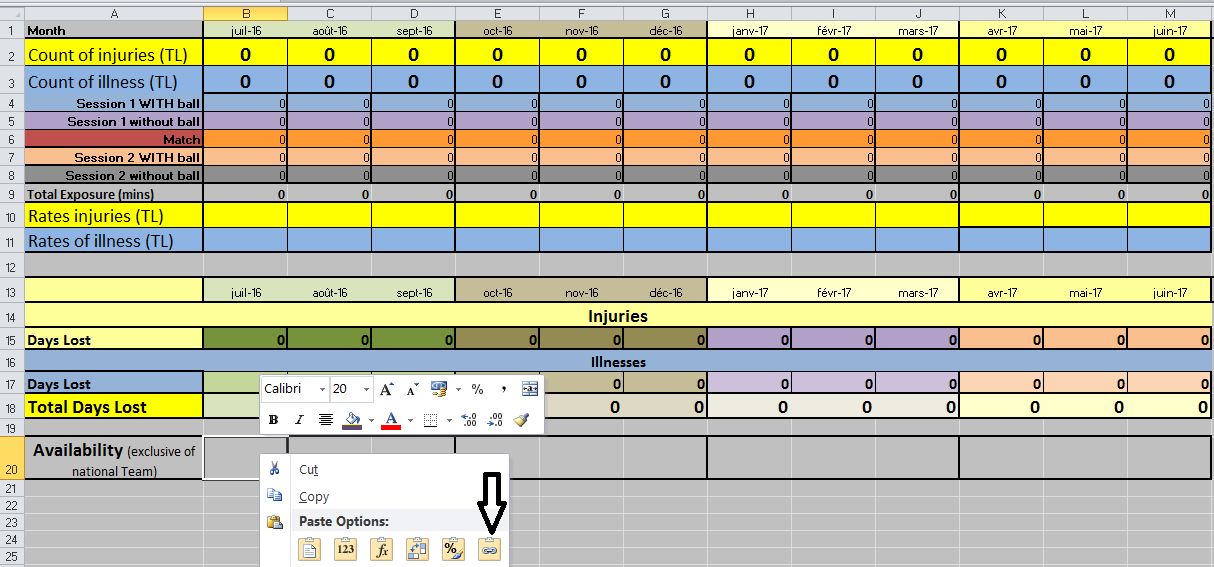


**Fig. 14:** Pasting the selection in cell D-20 with special paste option ‘’Link’’.

Once these 2 selections imported in the ‘’injury-illness’’ File, the functionality will work and you will be able to have the complete reports of the injury-Illness surveillance program.

**7 How to use the “Filter option’’ in Excel:**

The Excel file contains a specific feature called “Filter”; this feature is used when you want to display records for specific information in your data

1. Open the excel sheet, and select with the curser the empty column between the top column that contain the main information and the first player. Click where the red arrow is on Figure 1.


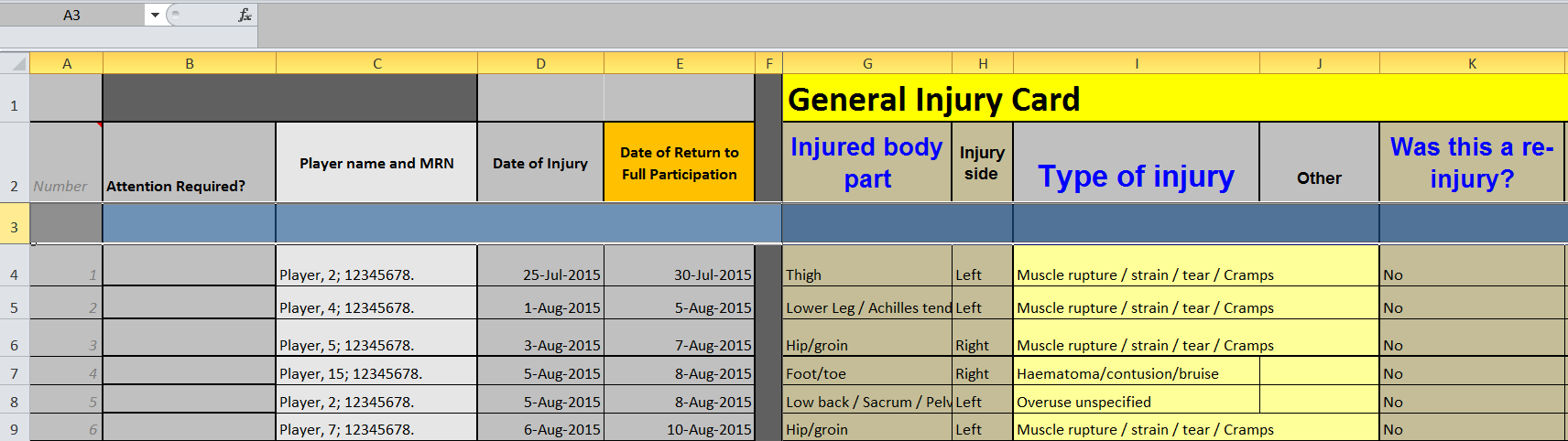


Fig. 1

1. Go to the **‘’Data menu’’** tab and then click filter (Figure 2-A and 2-B)

 
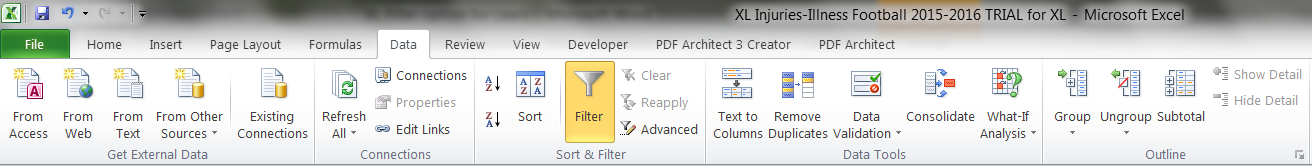


Fig. 2-A. ‘’Data Section’’ location in each XL Sheet.


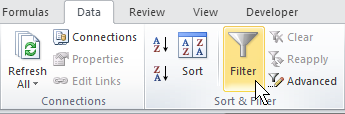


Fig. 2-B. Filter option in the Data menu section.

When you do so, the **‘’Filter option’’** will be set and ready for all the columns with a small arrow on the right side of each column (see figure 2-C)


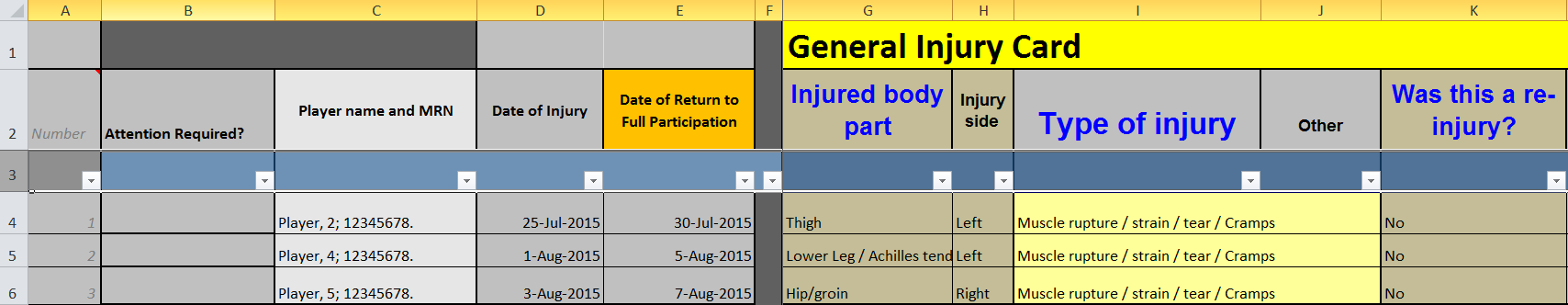


Fig 2-C. Filter option ready to use for filtering the sections under the highlighted line (line 3 in this case). By clicking the little arrow on fig 2-C one can access the filter options of the concerned column (in this case ‘’Injured Body Part’’)

3 - Click the arrow under the ‘**’Injured body part’’** to display the filter options (Figure 3):

**Important:** This example concerns ‘’Injured body part’’, but you can use the feature for any column (exp: Type of injury or Match/Training columns)


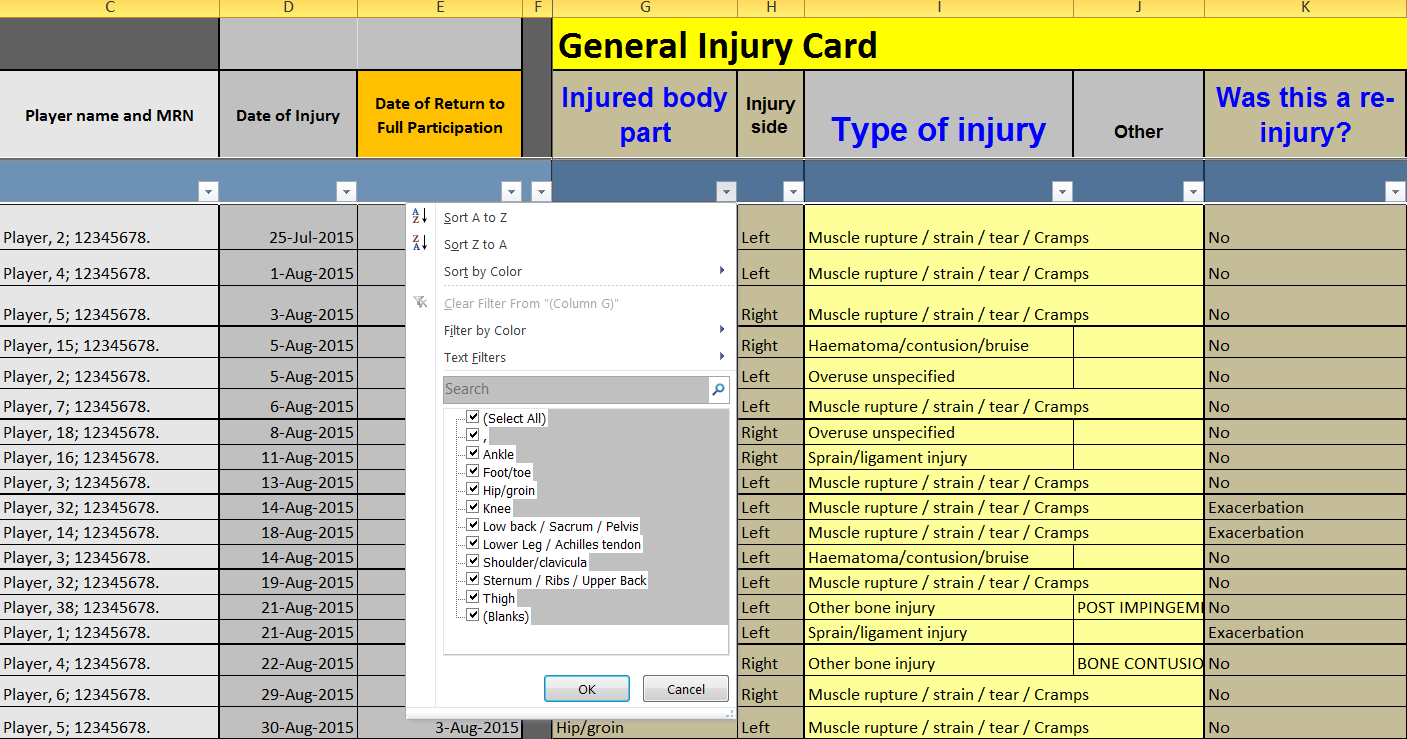


Fig. 3. Filter option displayed and ready for use.

1. Click on “Select All” to clear all the ticked boxes, and **then click/tick the check box next to any** ‘’Injured Body Part’’ you would like to know about (Figure 4)


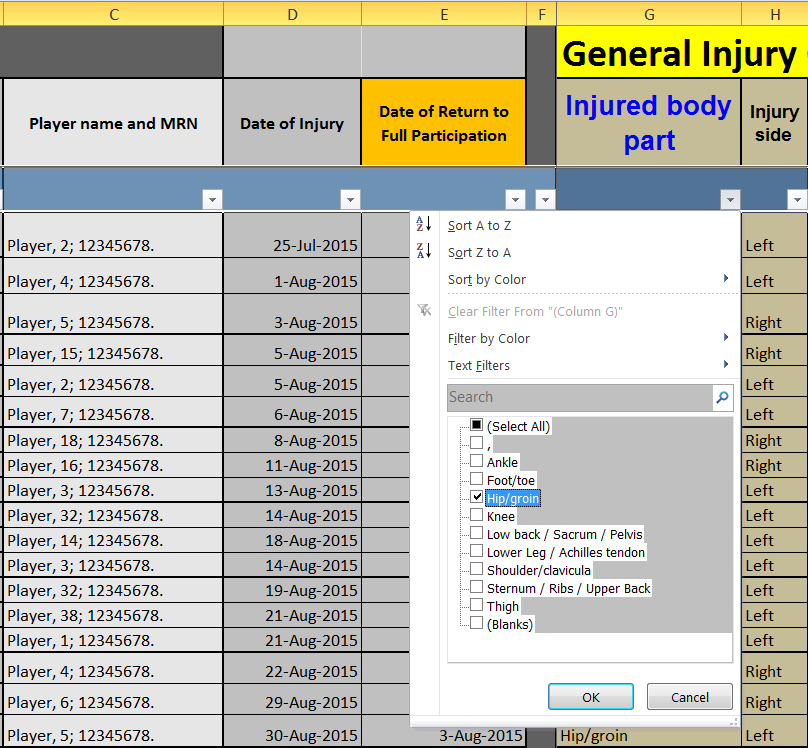


Fig. 4. All check boxes cleared and the ‘’Hip Groin’’ location was chosen for filtering.

1. **Click OK.**

Result. Excel only displays the injuries in Hip/Groin (Figure 5)


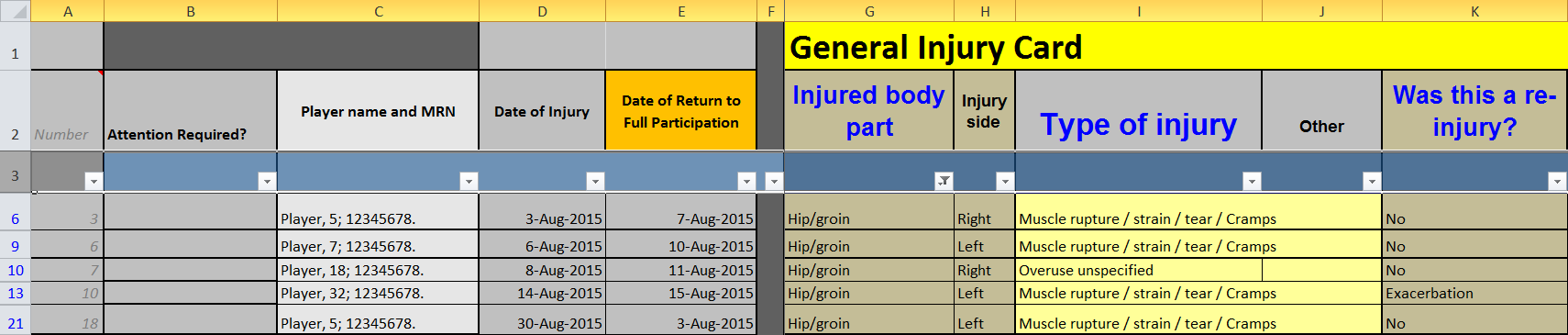


Fig. 5. Displaying the 5 cases of Hip/Groin injuries out of all other cases of injuries.

**With this selection, you can proceed with your proper exploitation of data**. For instance, you can calculate proportions of this count compared to the whole number of injuries. If you have 50 injuries in total and that the filter gave 5 injuries of the ‘’Hip and Groin’’, then the proportion is 10%.

1. To remove the filter, on the Data tab, click Clear. To remove the filter and the arrows, click Filter. (Figure 6)


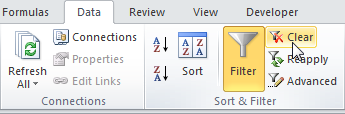


Fig.6. Clearing the ‘’Filter option’’ and come back to the original format of XL file with all injury cases (unfiltered).

**8. Sending the forms to Aspetar (Study group)**

The e-files will be given to each club with specific names. These will have to be used for the whole season without changing the file’s name.

The Hardcopies of each ‘’injury’’ or ‘Illness’’ will have to be archived at the site of the Club/Federation in the local players’ files. In case any IT issue occurs, these files will be used to re-enter the eventually lost data. But as the e-files will be saved with the Doctors and at Aspetar on 2 computers, this eventuality is unlikely to occur.

It is strongly recommended to transfer any injury/illness to the e-file as soon as possible to allow timely calculations of injury related indexes.

Every month, each club contact person will have to send the XL files related to the injuries/Illnesses and Exposure to the Contacts persons at Aspetar (Please do not send the cards as all the information from those has already been entered on the XL Files).

The information sent from the QSL Clubs will be anonymously averaged and sent back to all Clubs for comparison purposes and optimised injuries/illnesses management.

Any comment on the needed information to be sent and the way of information exchange is welcome.

**8.1 How to send the Files?**

Please send the ‘’XL files’’ by email to:

[montassar.tabben@aspetar.com](mailto:montassar.tabben@aspetar.com)

with cc to: [rima.tabanji@aspetar.com](mailto:rima.tabanji@aspetar.com)

N.B.: WE STRONGLY RECOMMEND THAT THE FORMS ARE COMPLETED ON ELECTRONIC FORMAT. PLEASE DON’T HESITATE TO CONTACT THE ‘’Program GROUP’’ IN CASE YOU ARE NOT ABLE TO USE THIS FORMAT AND WE WILL ASSIST YOU HOW TO DEAL WITH THE MATTER.

**8.2 When to send the forms?**

WE KINDLY REQUEST THE USERS TO RESPECT THE DEADLINES, AS A MATTER OF DATA RELIABILITY.

PLEASE SEND THE DATA MONTHLY, most preferably during the first week of the following month.

**8.3 Confidentiality**

All data on individual players and teams will be strictly confidential.

**8.4 Questions**

Do not hesitate to contact the study group at any time of the study for clarifications.
