# Supplementary material for: Lessons Learned from Implementing Injury and Illness Surveillance in Professional Football: Introducing a New Implementation Framework
Source: Sports Med. 2025 Jul 11;55(10):2375–85. doi: 10.1007/s40279-025-02276-5 (PMC12513881; doi:10.1007/s40279-025-02276-5)

AFC Injury & Illness Report 2021

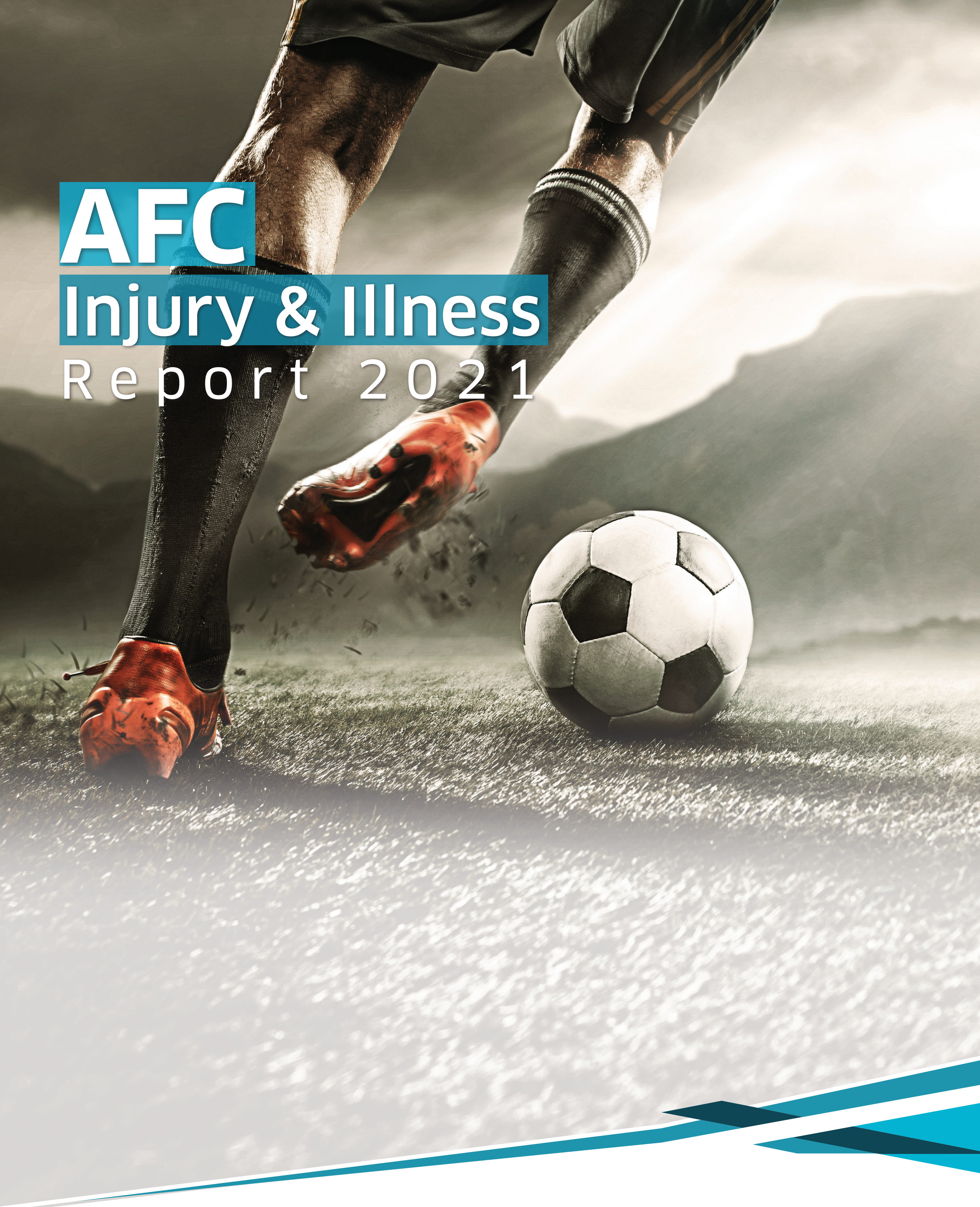

ASPETAR سبيتار

AFC CHAMPIONS LEAGUE ©AFC2008

Please note that this is just a first sample draft report which has been edited based on fake data

Dear Colleague,

We are pleased to present the report for the inaugural season of the Asian Football Confederation (AFC) Champions League Injury and Illness Surveillance Programme. This Programme would not have been possible without your commitment and cooperation. For this reason, we would like to present our most sincere gratitude.

We hope that the information presented in the following pages will be helpful to improve the injury and illness risk management strategy within your team.

In the following pages, data on the exposure, injuries and illnesses of your players are presented. May you have further specific question, we would be happy to provide you further information.

We are happy also to support you in the data interpretation, if you wish so. When reading this report, please consider that this is the first year of the surveillance, therefore the amount of some data may be limited.

The AFC research team

DEFINITIONS

1. Training exposure

Training exposure is defined as any team-based or individual physical activities under the control or guidance of the team's coaching and/or fitness staff that are aimed at maintaining or improving players' skills or physical condition/ recovery.

2. Match exposure

Match exposure is defined as play between teams from different clubs.

3. Injury

A ''time-loss" definition of team sport injury is used: An injury is any physical damage that occurs during training or match and results in the player being unable to fully participate in one or more training or match-play sessions.

4. Illness

"Time-loss" illnesses are collected: A physical or psychological complaint or manifestation experienced by an athlete that occurs at any time (during or out of football activities) and which causes absence from football activities"

5. Onset of injury

Injuries are classified as 'Sudden" or ''Gradual", according to their onset. If the injury is resulting from a specific incident clearly recalled by the athlete, it's classified as ''Sudden Onset". If the injury developed progressively over time (days/weeks/months, it's classified as ''Gradual Onset".

6. Injury burden

A combined measure of the frequency (injury rate) and severity (days of absence) of injuries. Injury burden is expressed as the number of days of absence per 1,000 hours of exposure. Example: Team A with 10 injuries in 5,000 hours of exposure, each resulting in an absence of 10 days on average, has an injury burden of 20 days per 1,000 hours. Team B with 20 injuries in 5,000 hours, each resulting in an absence of 5 days on average, has also an injury burden of 20 days per 1,000 hours

DATA INTERPRETATION

PLEASE CONSIDER THE FOLLOWING WHEN READING AND INTERPRETING YOUR RESULTS:

This was the first year of data collection, the amount of data is still limited, therefore some results should be thoughtfully interpreted. In the case of injuries where it was not possible to establish a return to play date by the club medical staff (end of the season, player's change of club, etc), the club medical staff's or study group's estimated return date (based on the current scientific literature) has been used. Some injury data could consequently be based on approximations. Also, the number of months of data provided and the football season schedule differs between teams, and this may affect some comparisons.

GRAPHS IN YELLOW REPRESENTS ALWAYS THE TOTAL DATA (TRAINING + MATCHES), BLUE GRAPHS ARE FOR TRAINING AND RED GRAPHS FOR MATCHES.

Correspondence should be addressed to:

Asprev Department
29222 Aspetar
Sport City
Doha (Qatar)

Dr Cristiano Eirale
29222 Aspetar
Sport City
Doha (Qatar)
Tel: +974 4132696
Mob: +974 55897823
Fax: + 974 4132040
cristiano.eirale@aspetar.com

Prof Karim Chamari
29222 Aspetar
Sport City
Doha (Qatar)
Tel: +974 4413 2725
Mob: +974 3318 6096
Fax: + 974 4413 2020
karim.chamari@aspetar.com

Dr Montassar Tabben
29222 Aspetar
Sport City
Doha, Qatar
Tel: +974 44132557
Mob: +974 66180975
Fax + 974 4413 2020
montassar.tabben@aspetar.com

AFC CHAMPIONS LEAGUE ©AFC2008

MONTHLY TOTAL EXPOSURE

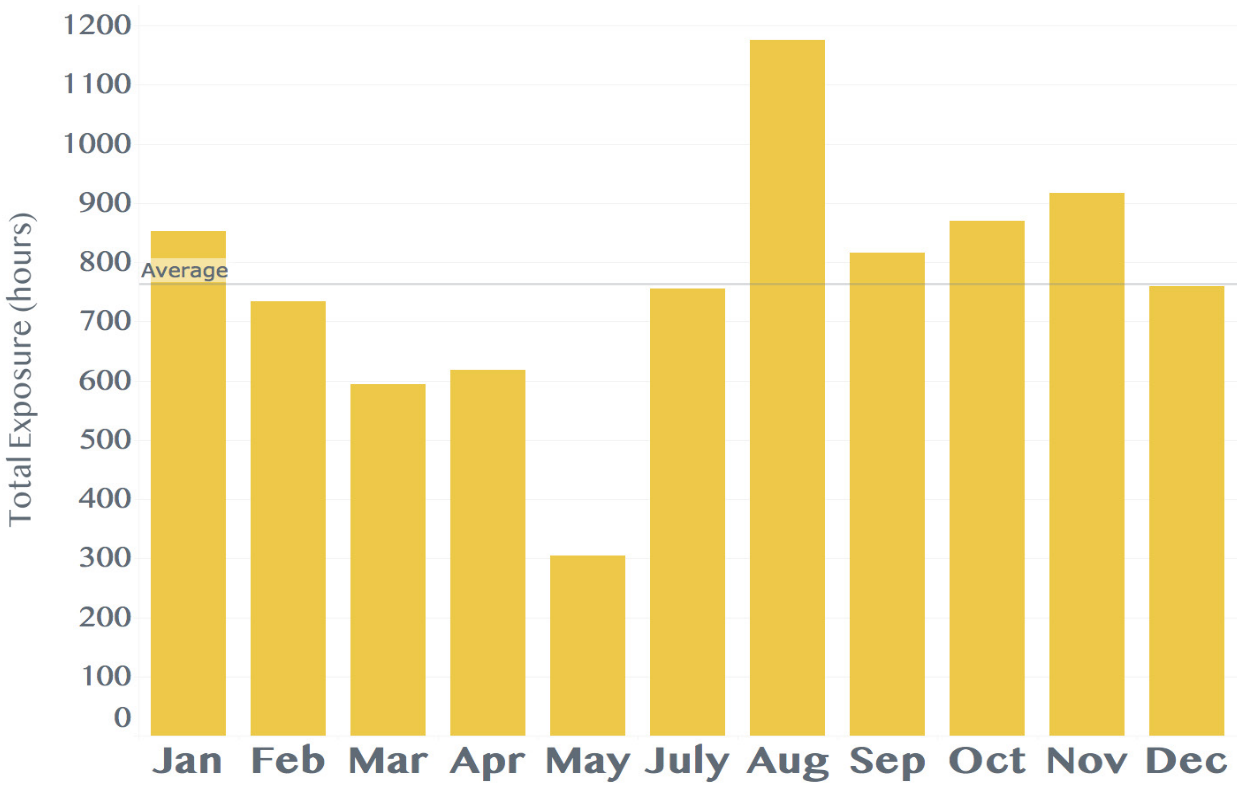

MONTHLY TRAINING EXPOSURE

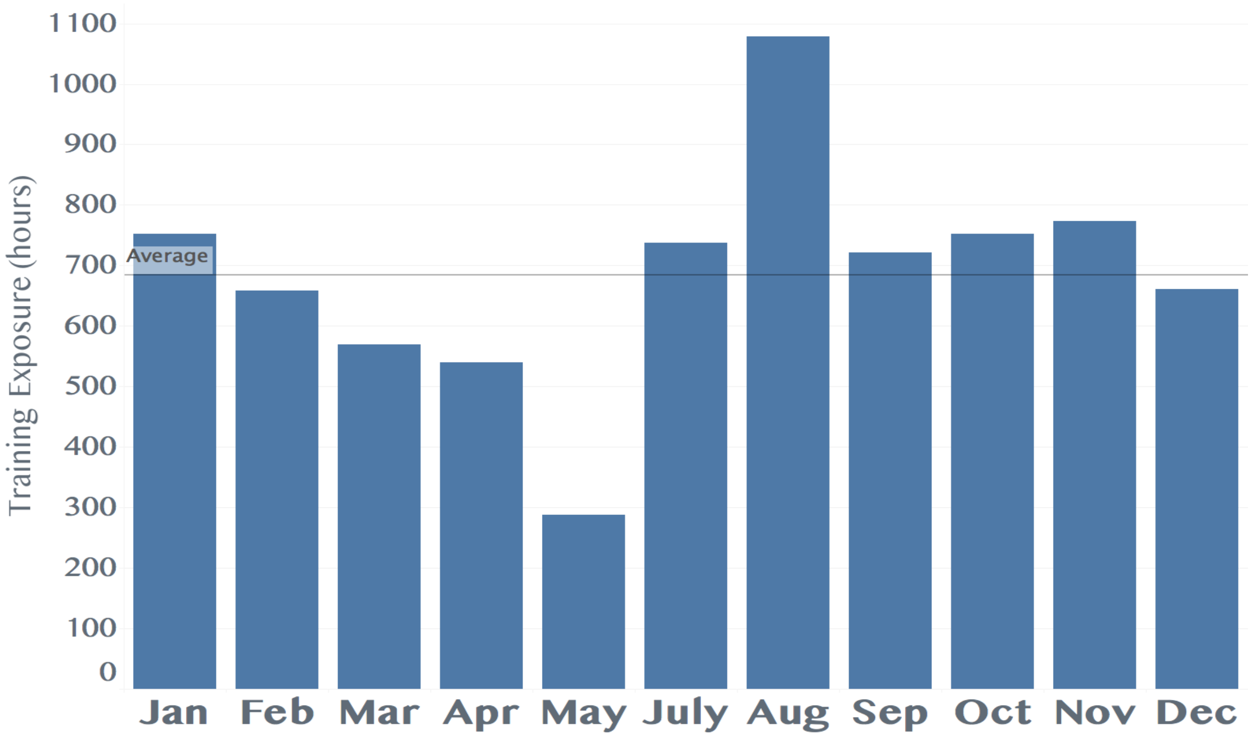

MONTHLY MATCH EXPOSURE

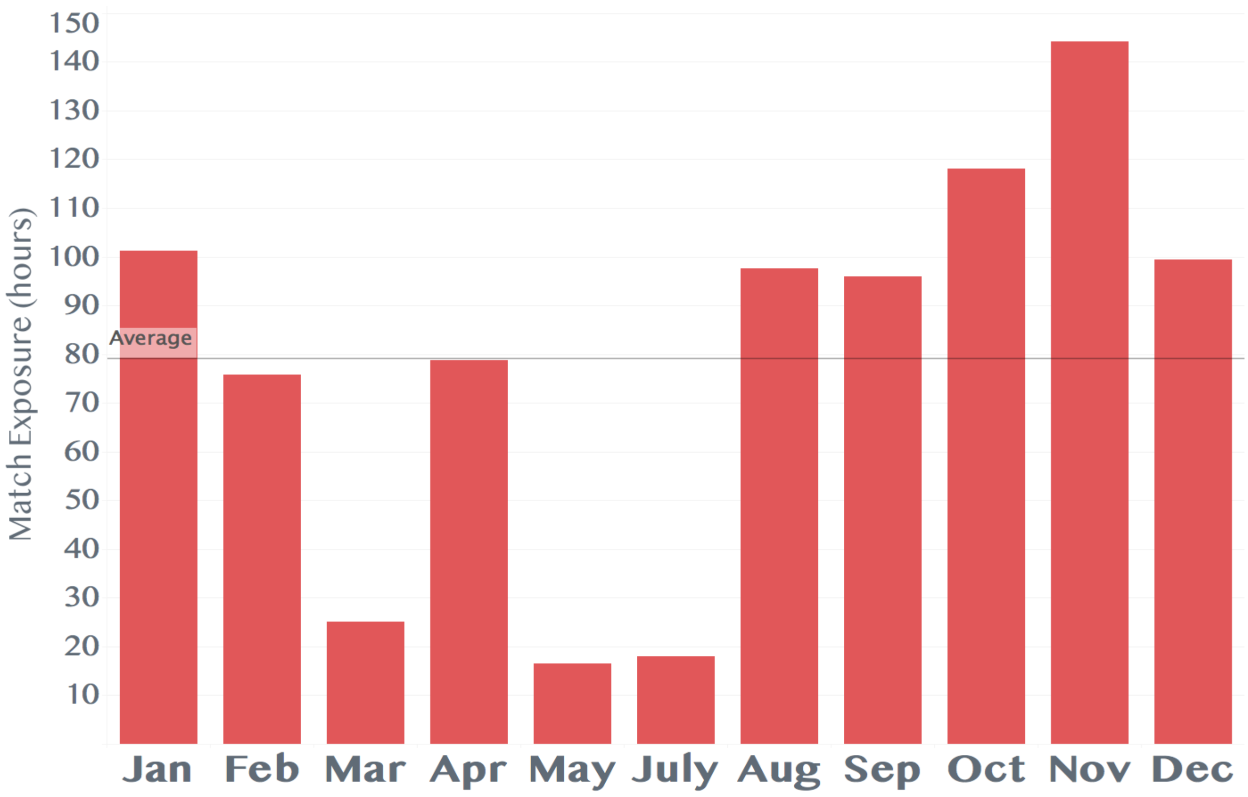

RATIO TRAINING TO MATCH HOURS

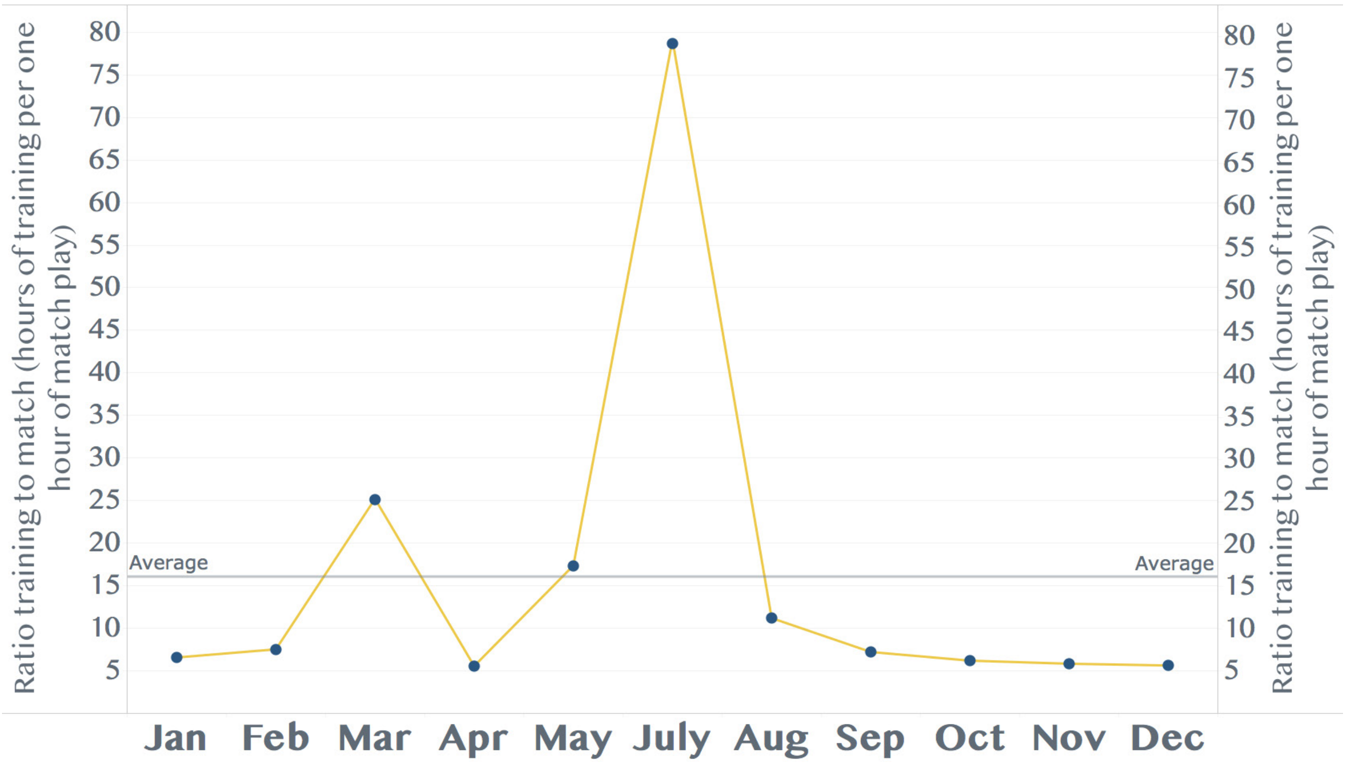

COMPARE YOUR EXPOSURE

TOTAL EXPOSURE MONTHLY AVERAGE

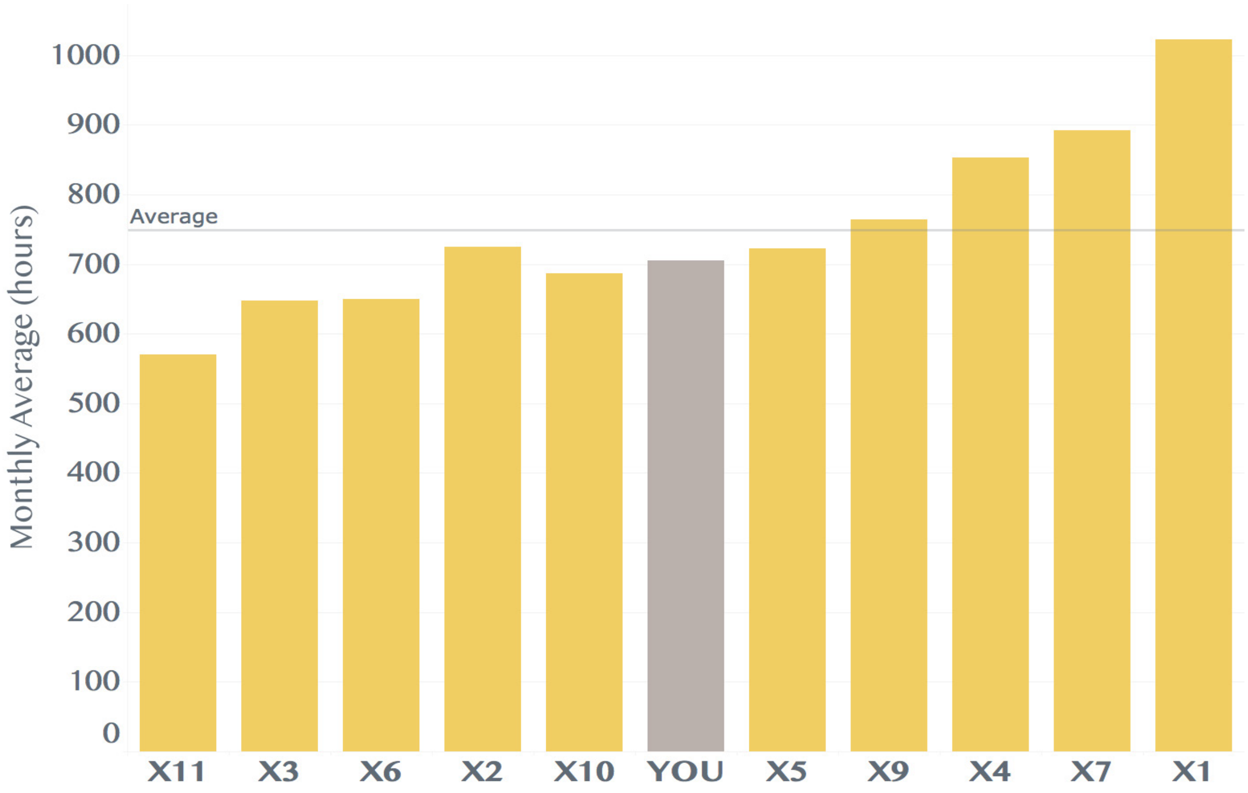

TRAINING EXPOSURE MONTHLY AVERAGE

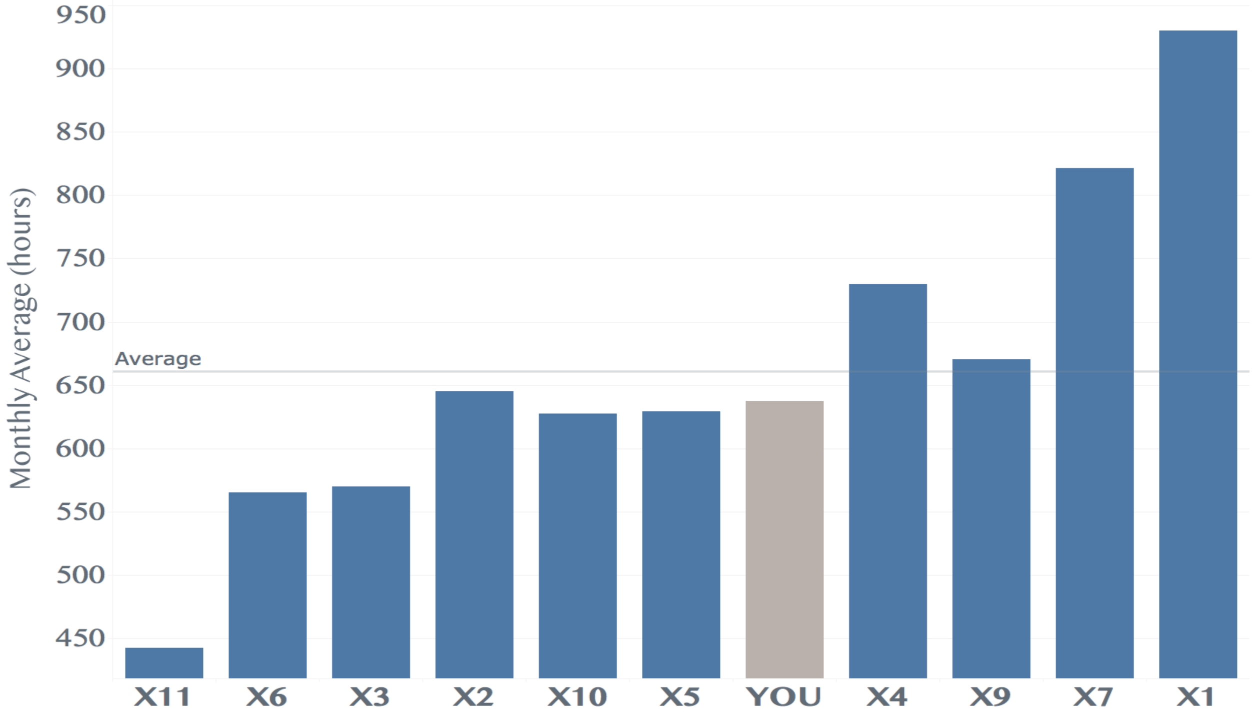

MATCH EXPOSURE MONTHLY AVERAGE

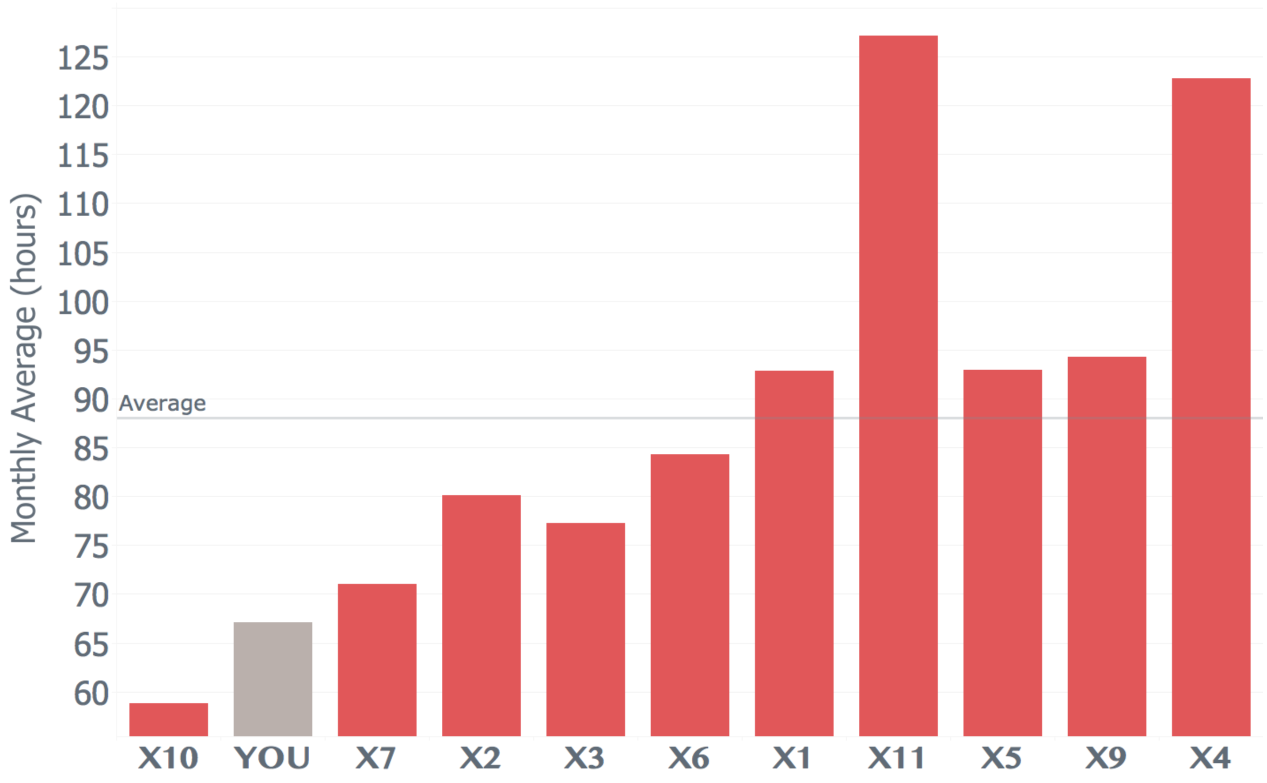

RATIO TRAINING TO MATCH HOURS

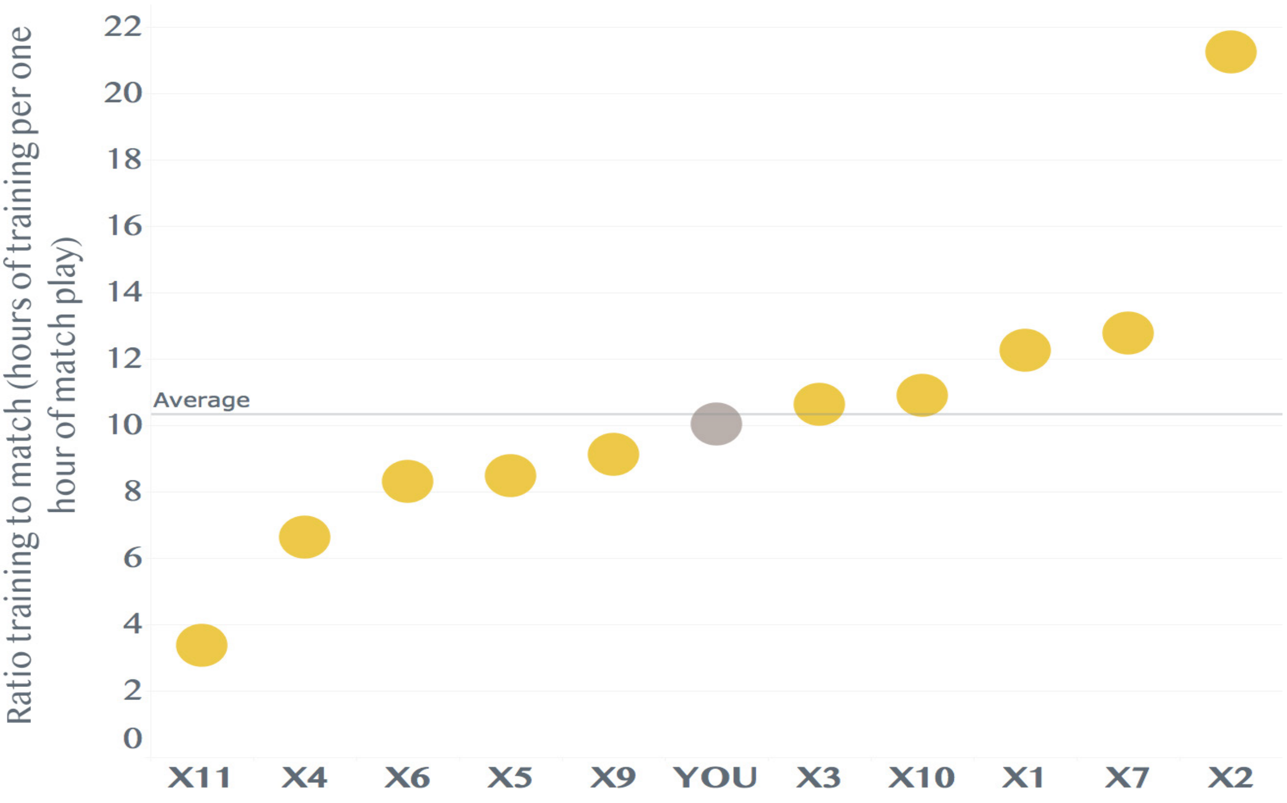

MONTHLY TOTAL INJURY RATE

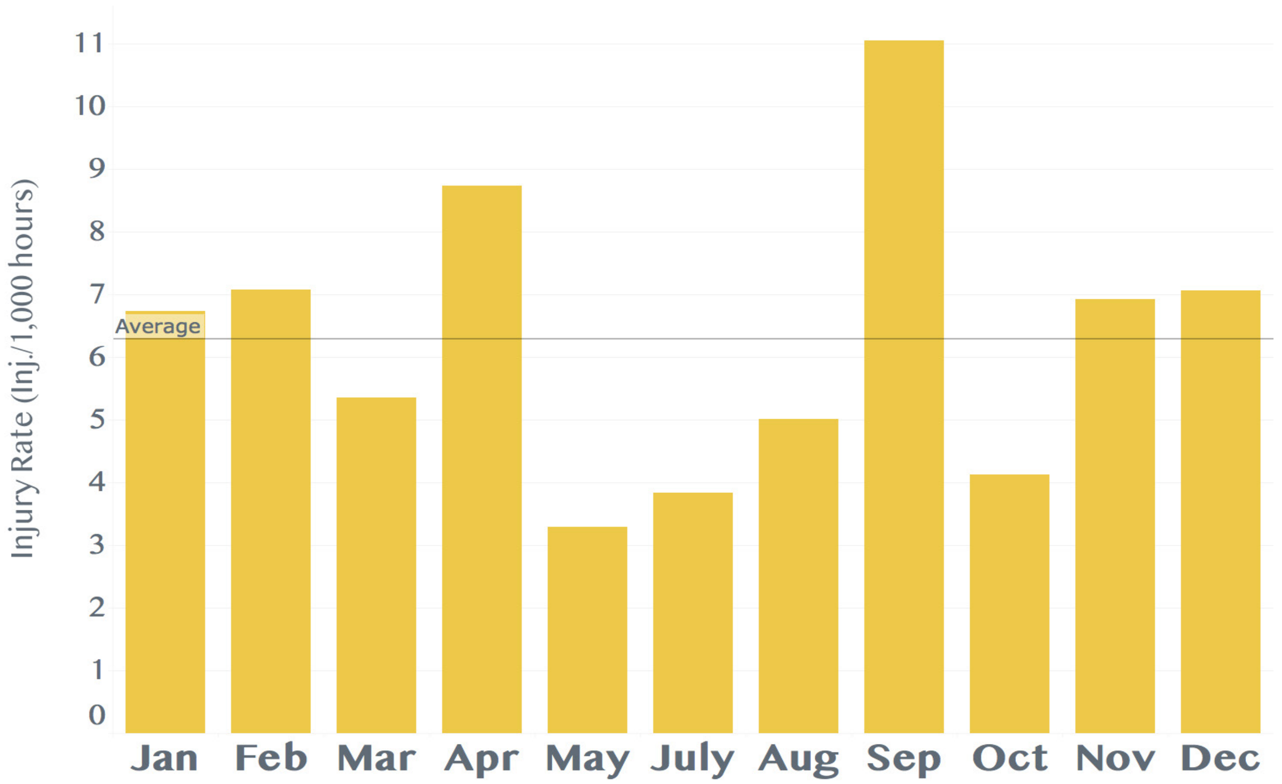

MONTHLY TOTAL INJURY BURDEN

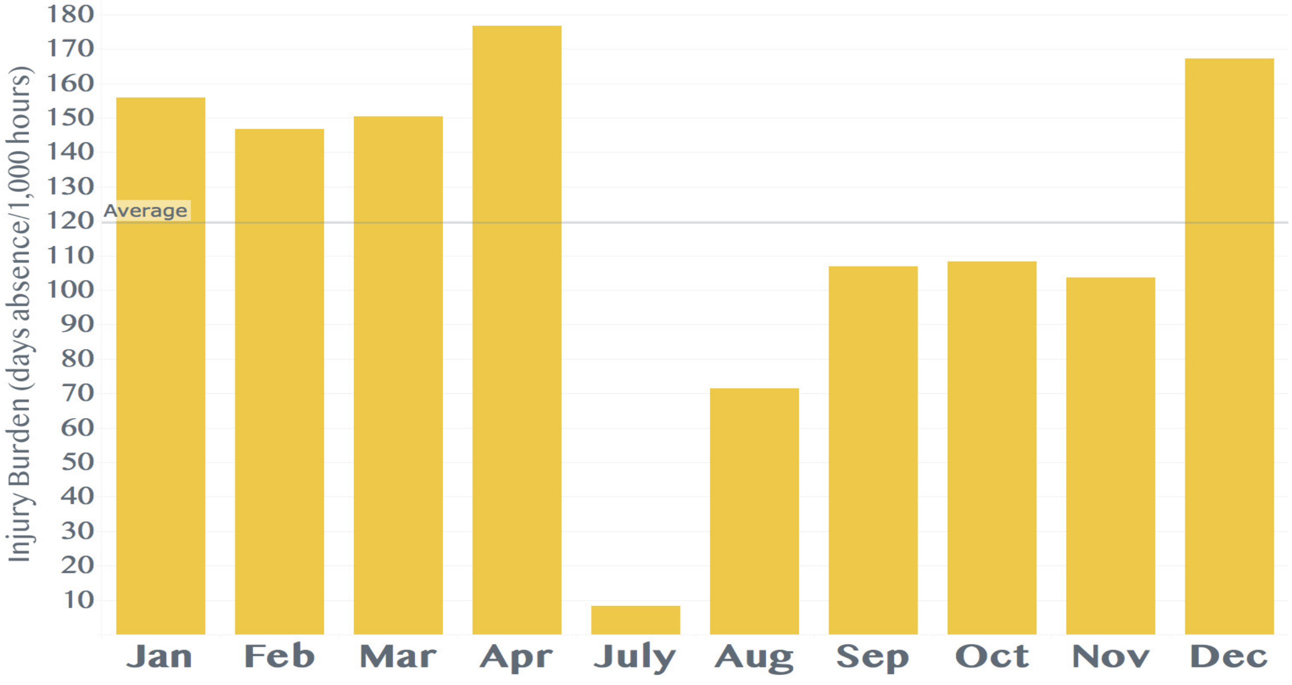

MONTHLY TRAINING INJURY RATE

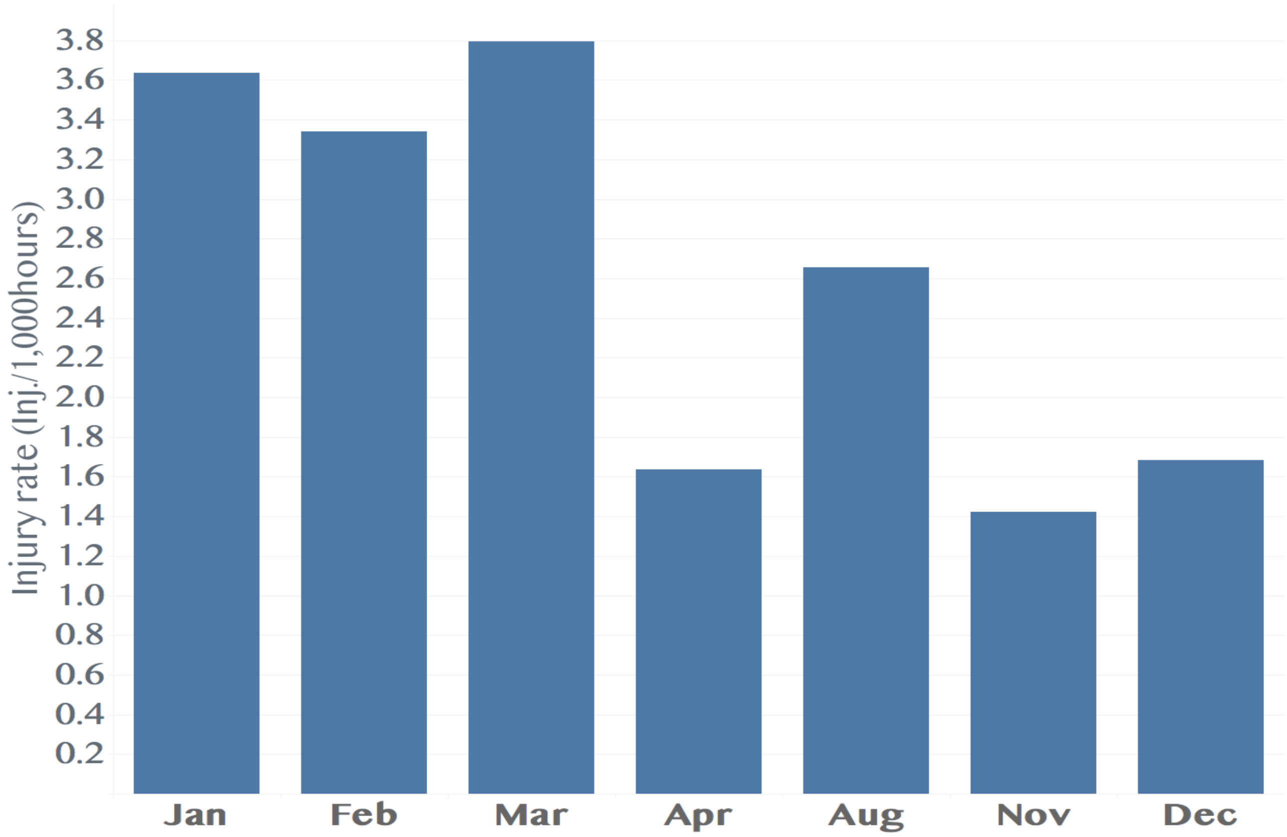

MONTHLY TRAINING INJURY BURDEN

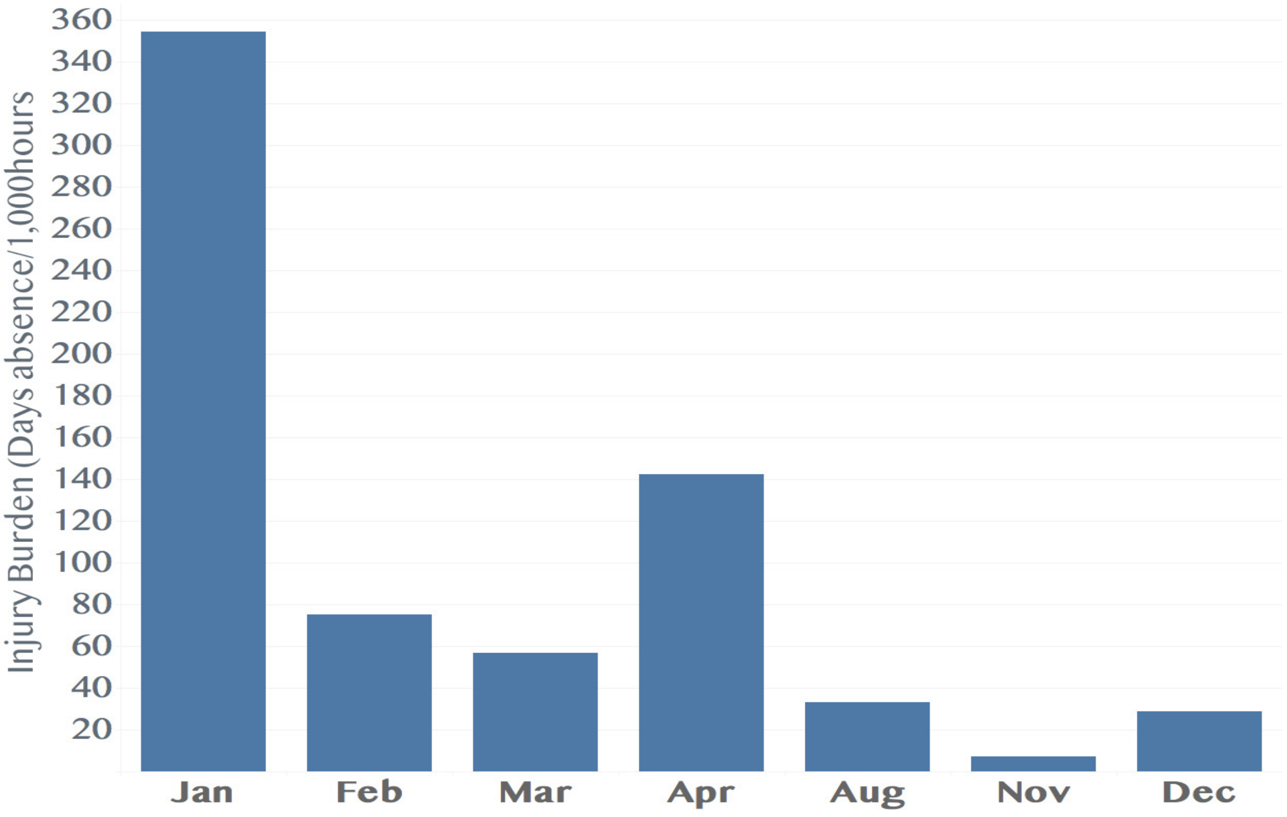

MONTHLY MATCH INJURY RATE

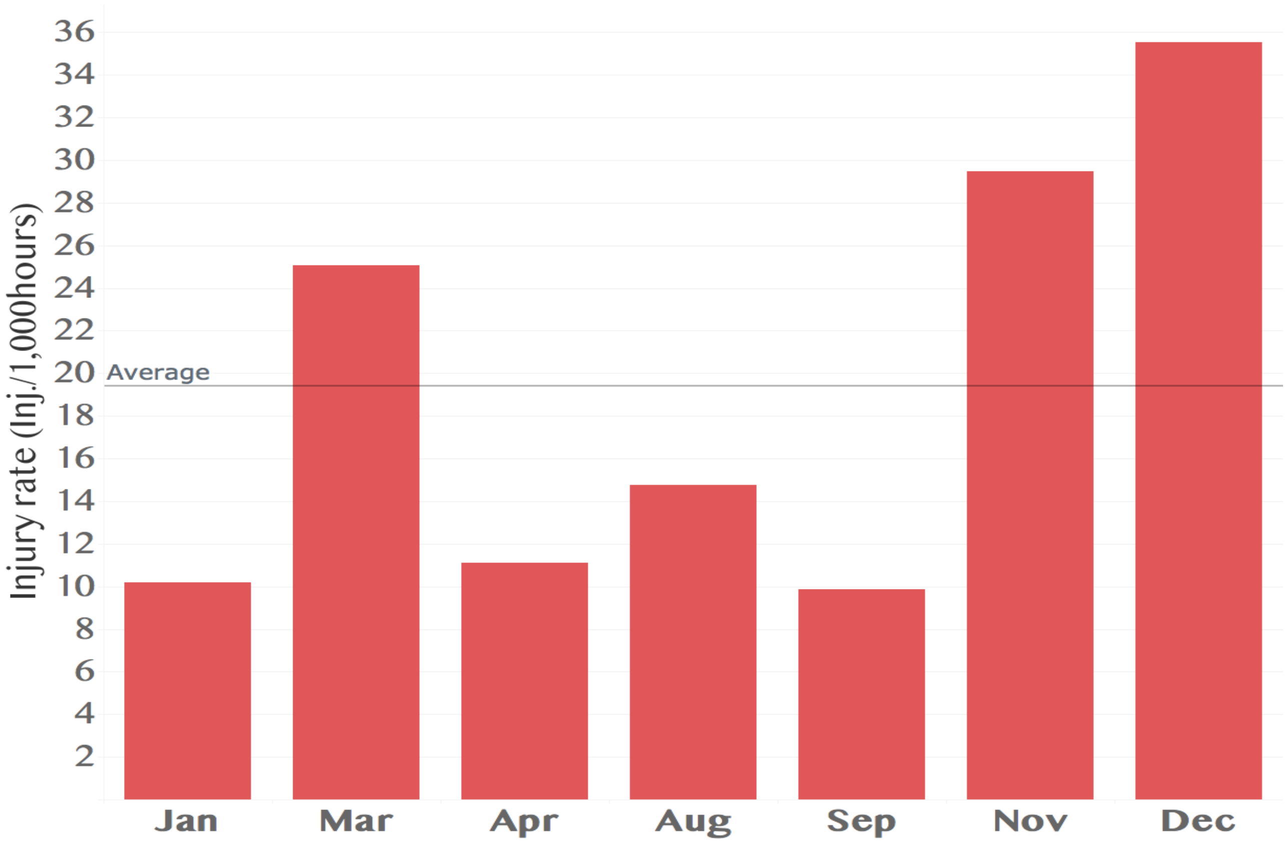

MONTHLY MATCH INJURY BURDEN

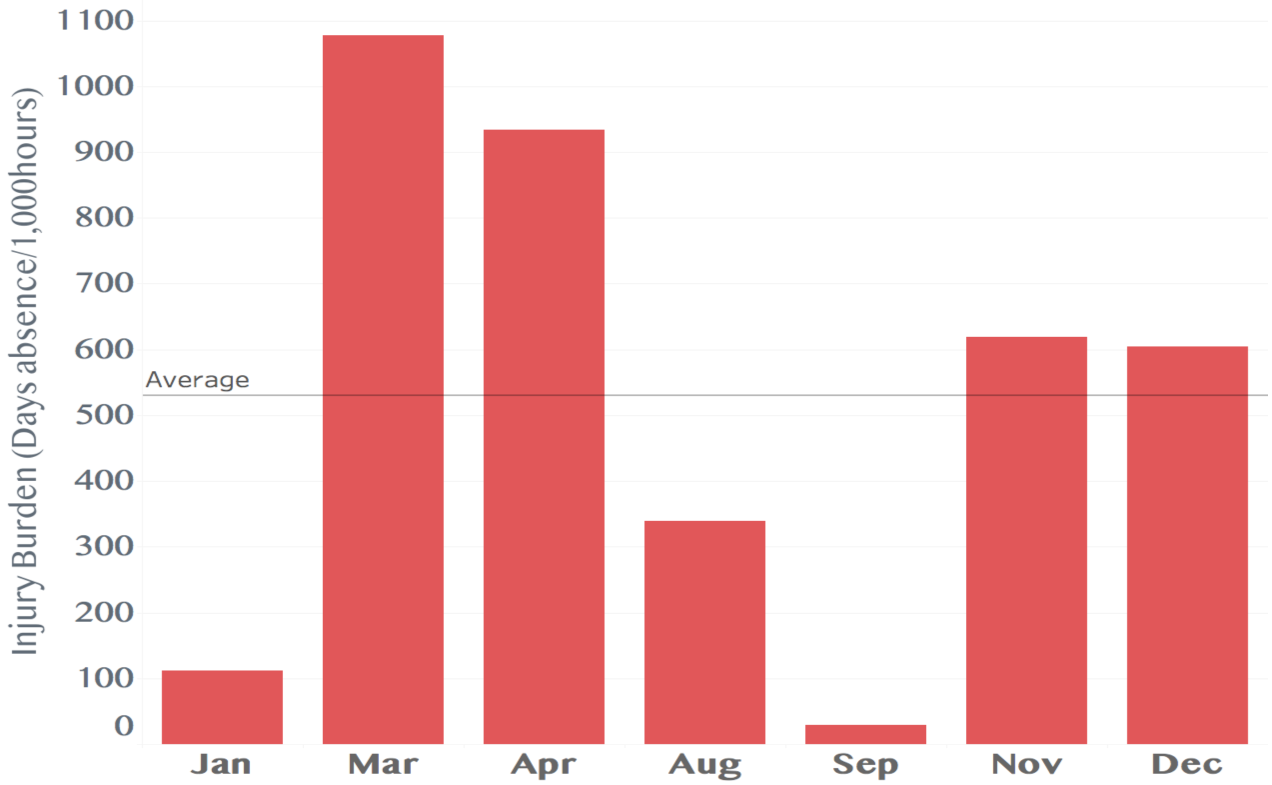

TOTAL INJURY RATE

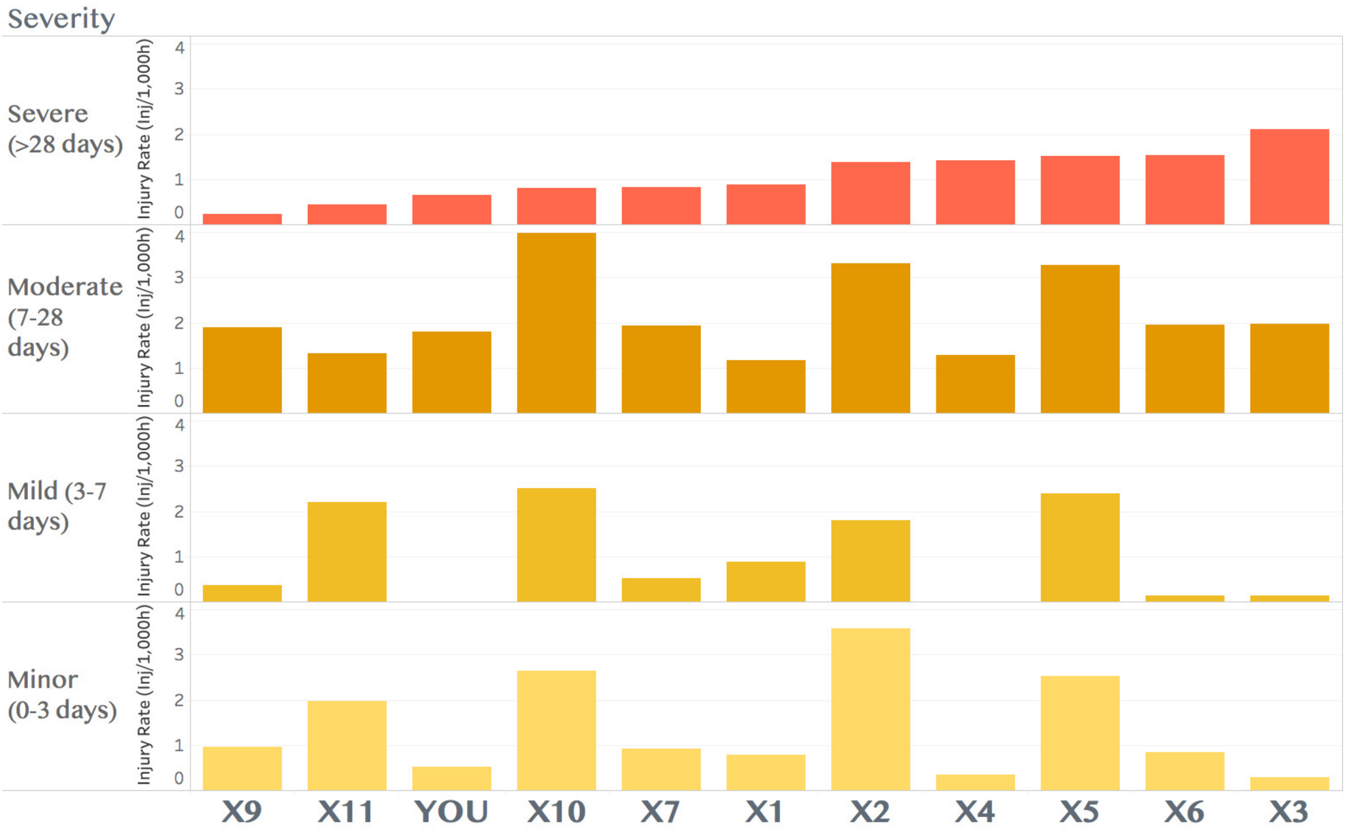

TOTAL INJURY BURDEN

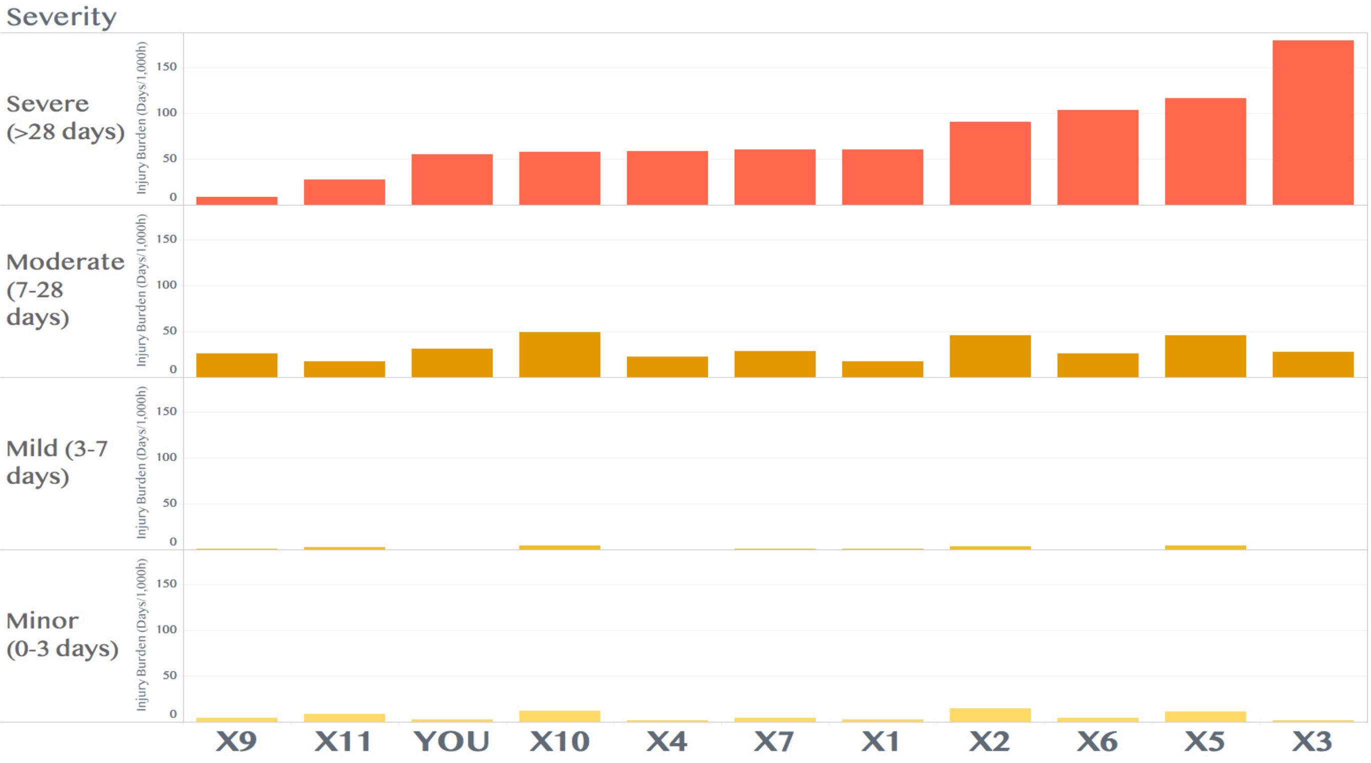

PERCENTAGE OF INJURY OF YOUR CLUB
(AVERAGE OF THE AFC)

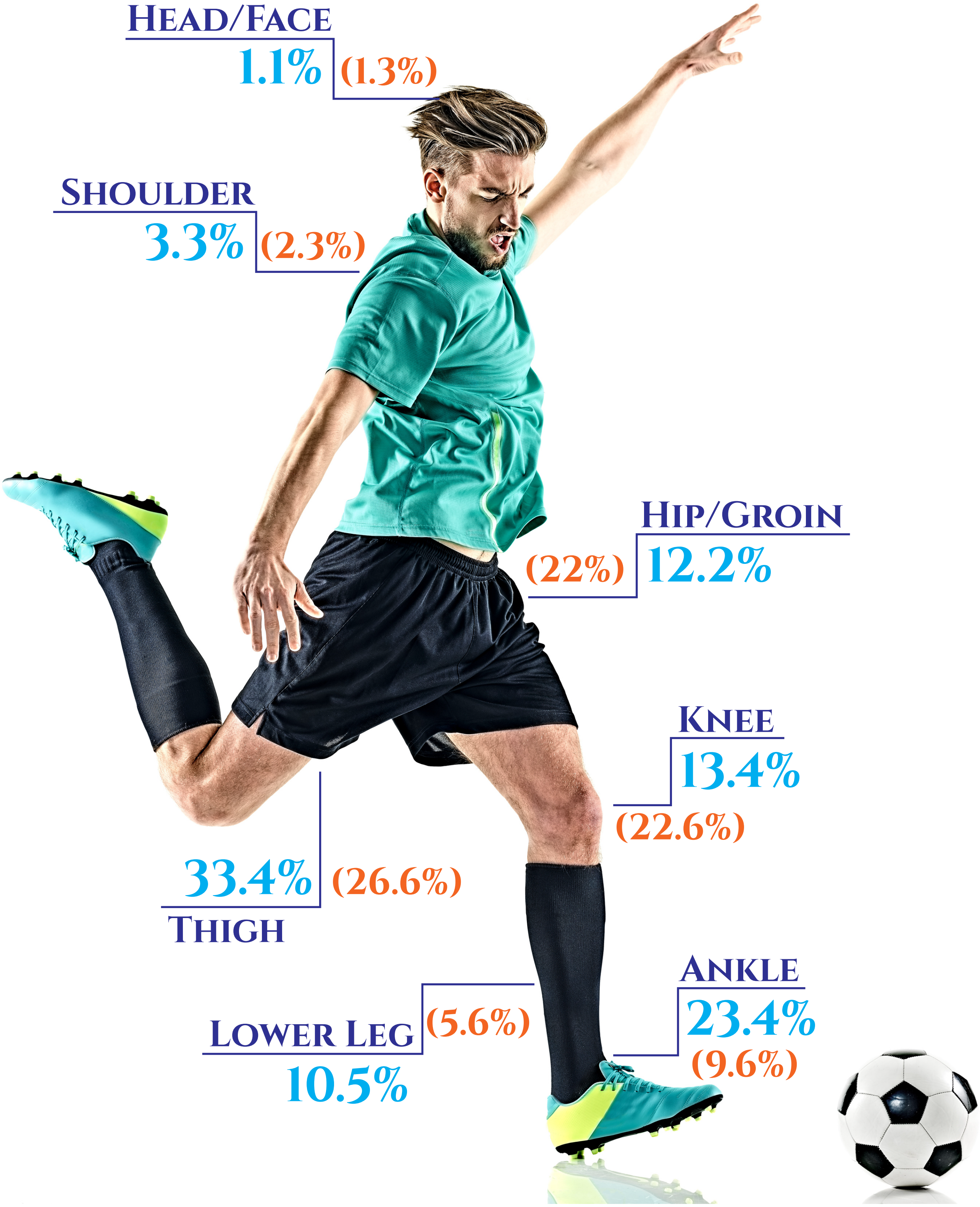

INJURY RATE -Inj/1000h- OF YOUR CLUB
(AVERAGE OF THE AFC)

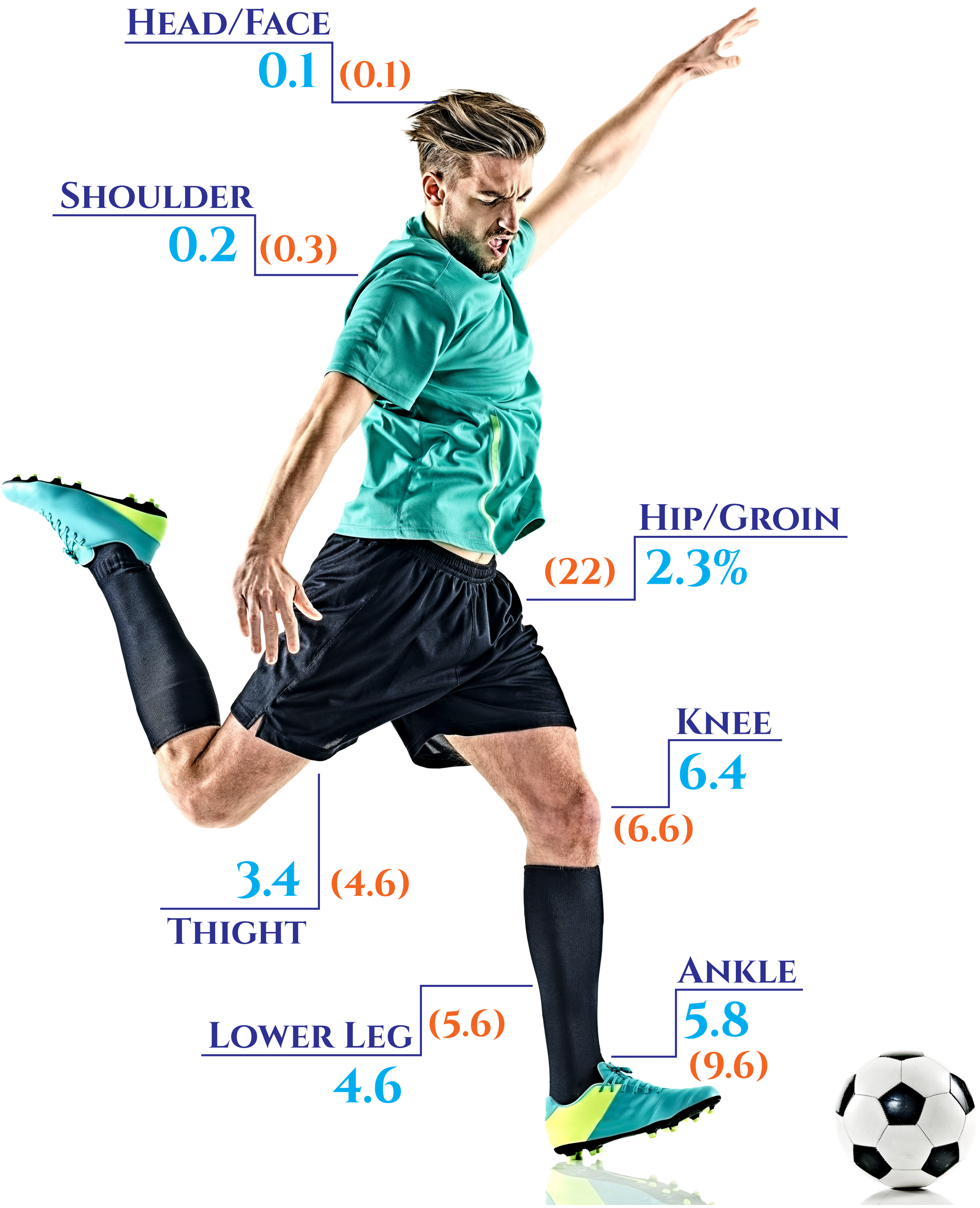

INJURY BURDEN -Days/1000h- OF YOUR CLUB
(AVERAGE OF THE AFC)

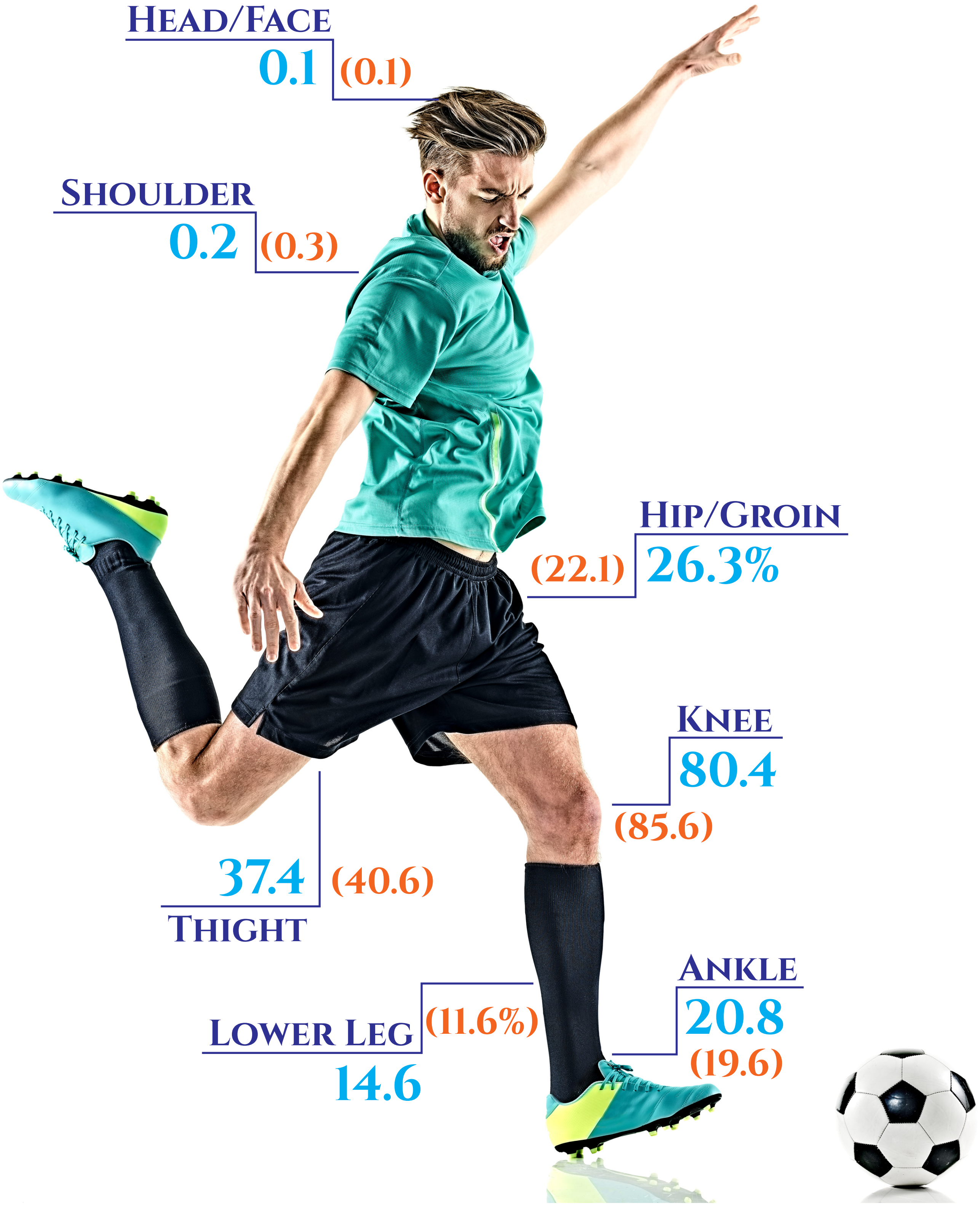

INJURY RATE BY TYPE

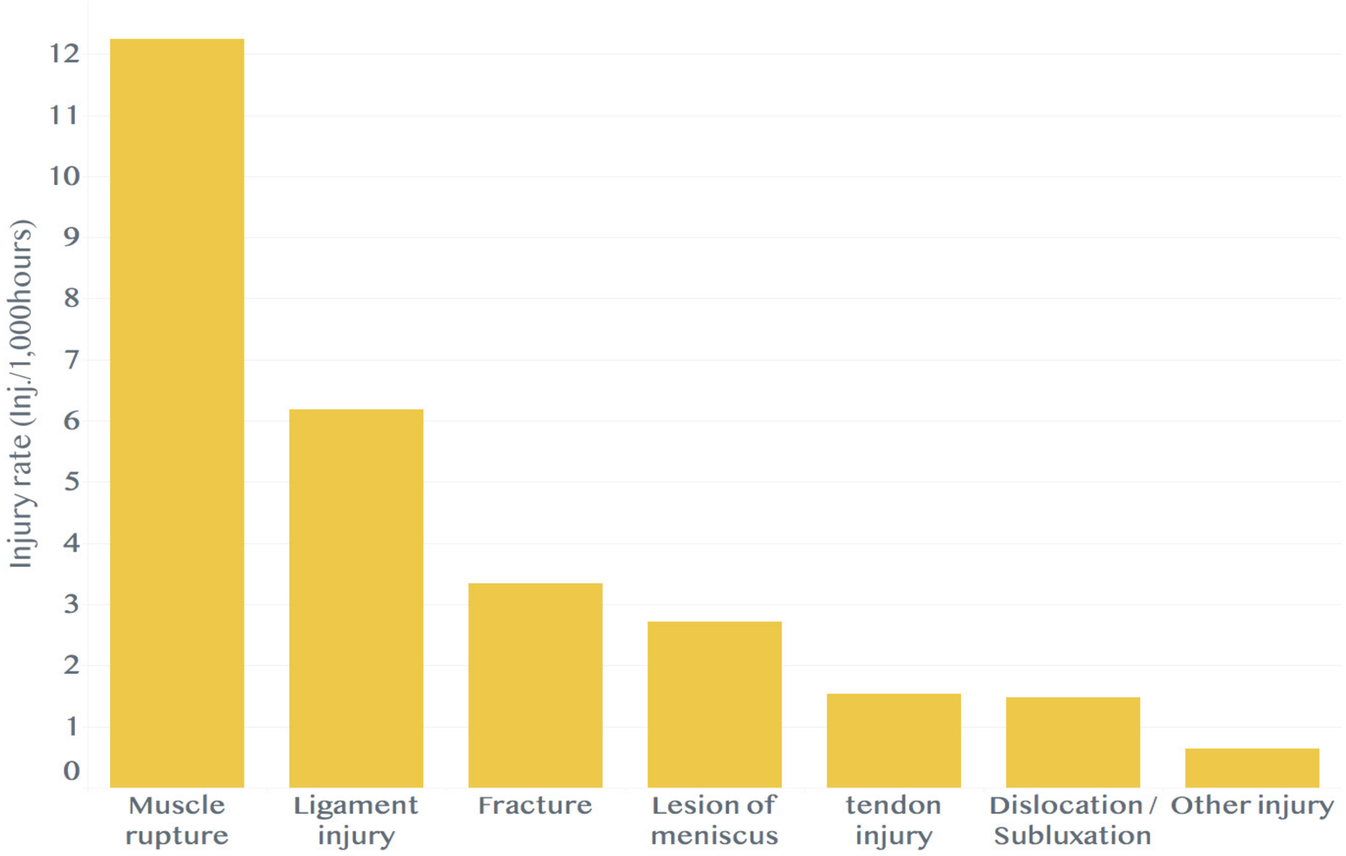

INJURY BURDEN BY TYPE

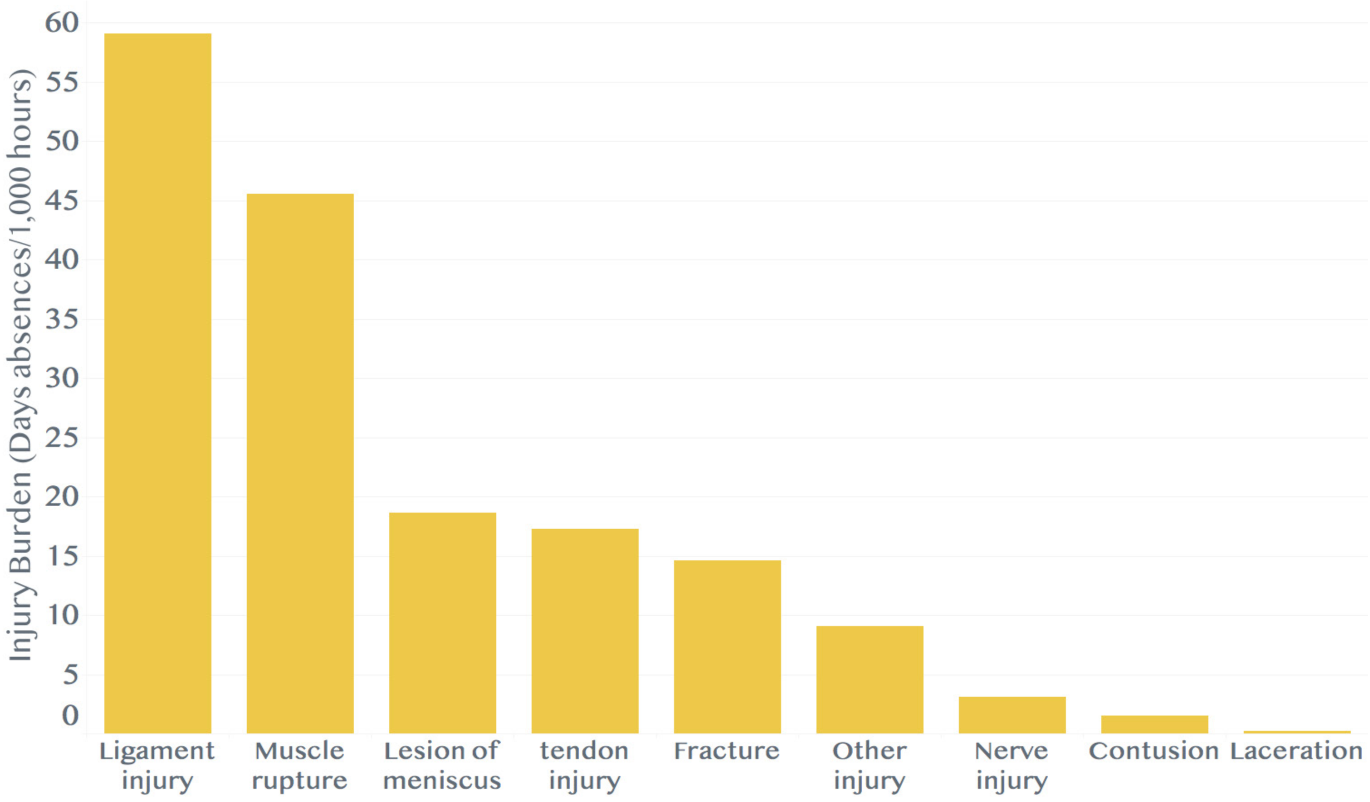

INJURY RATE BY MECHANISM

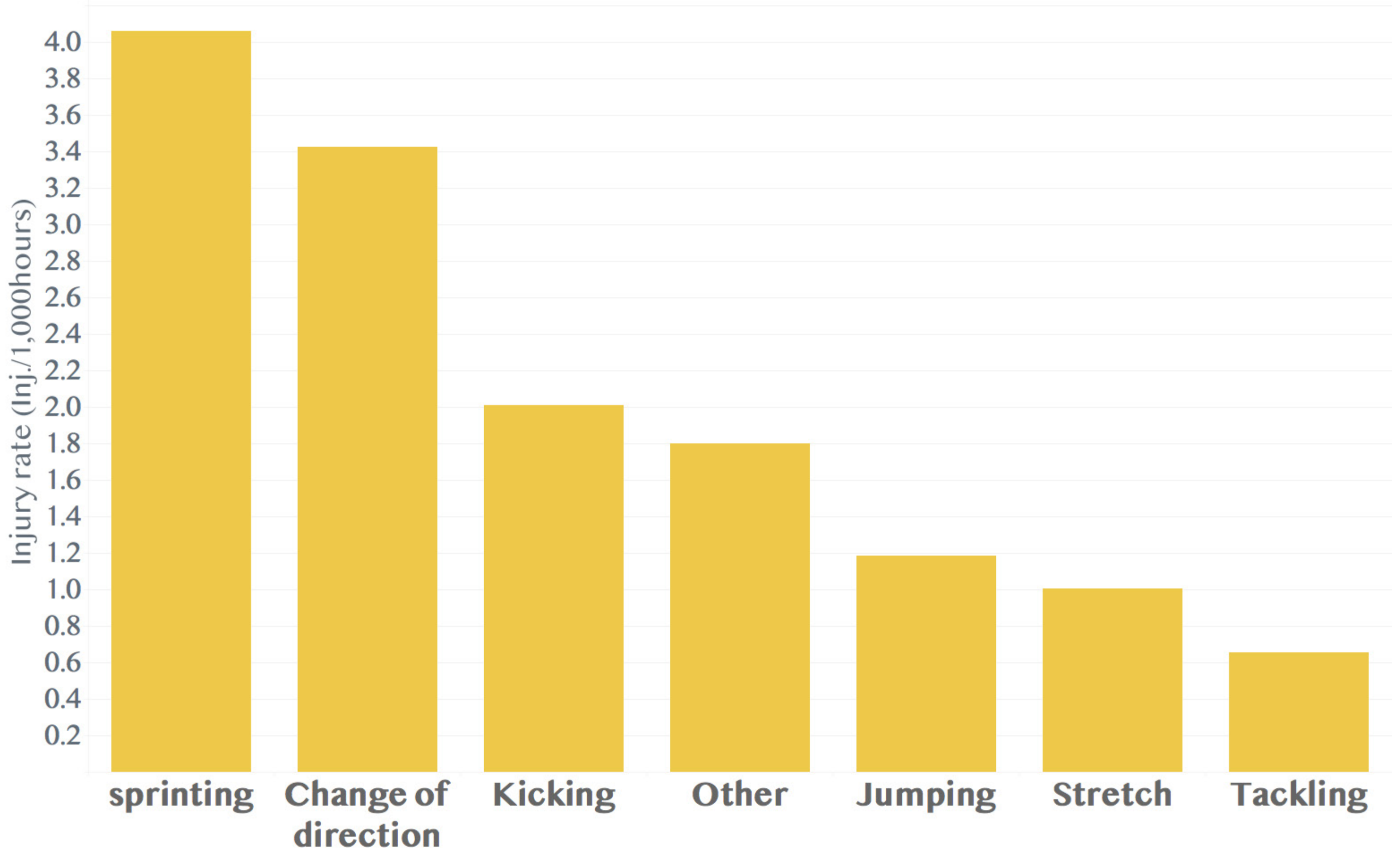

INJURY BURDEN BY MECHANISM

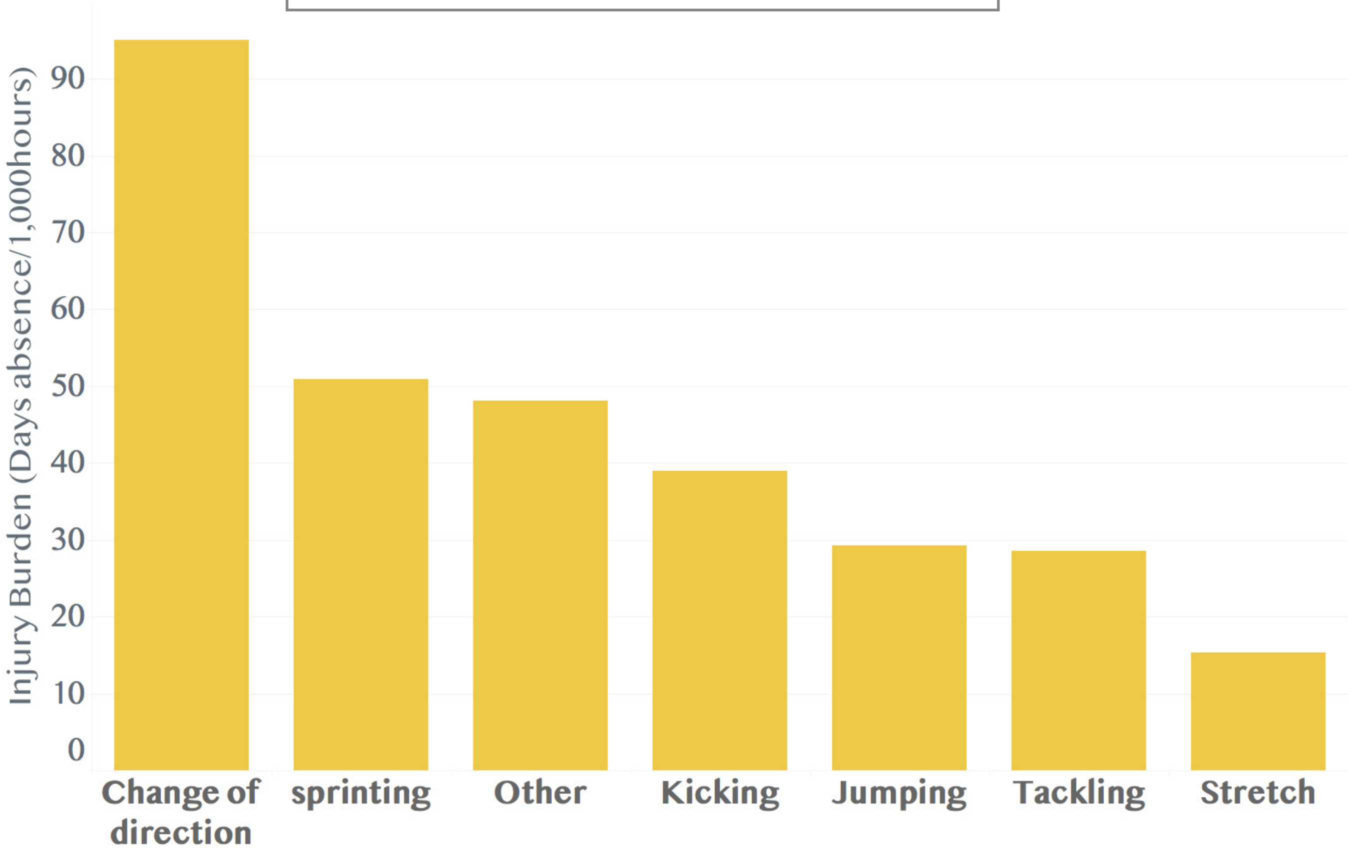

ONSET DISTRUBUTION BY TEAM

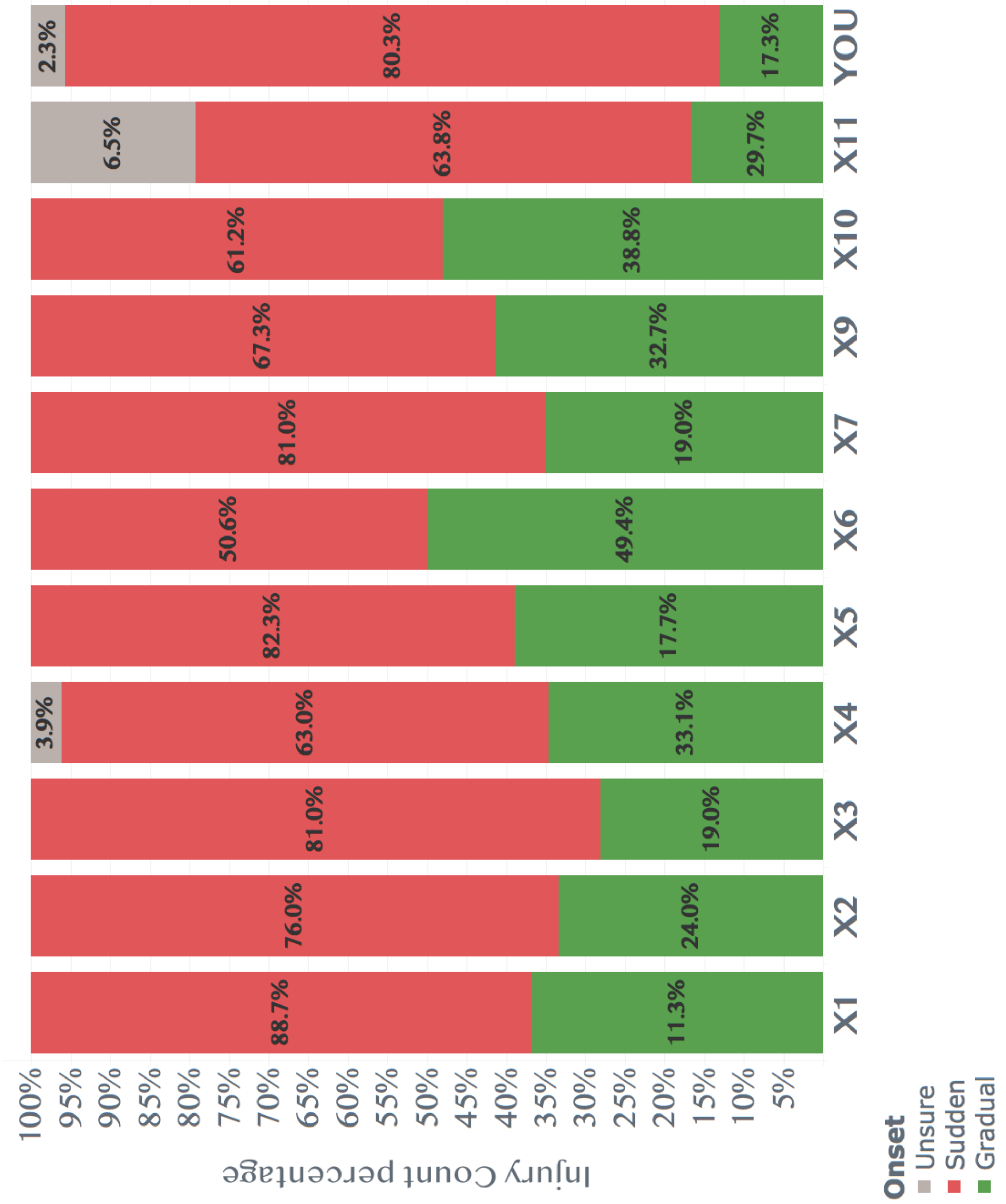

RE-INJURY DISTRUBUTION BY TEAM

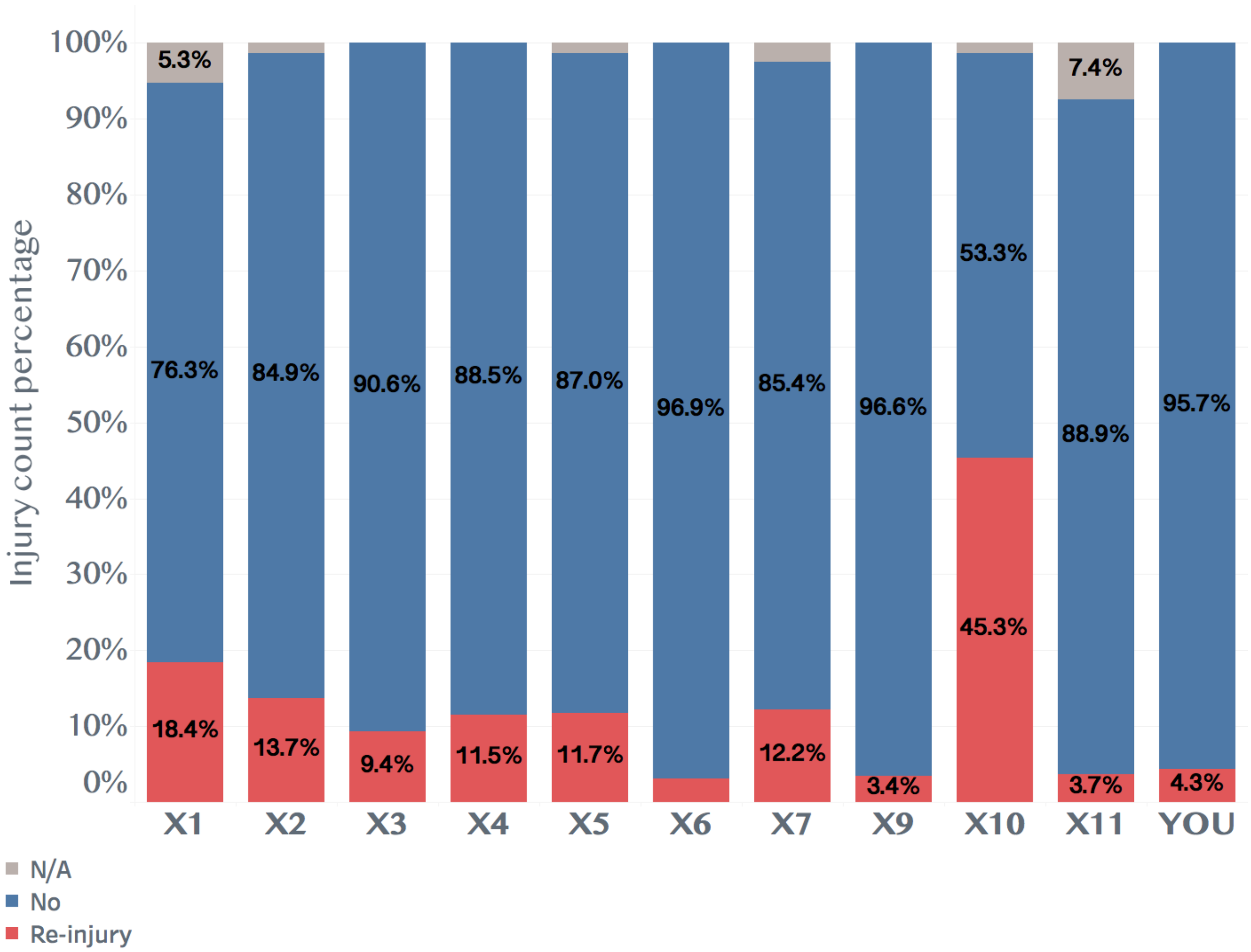

PERCENTAGE OF RE-INJURY OF YOUR CLUB
(AVERAGE OF THE AFC)

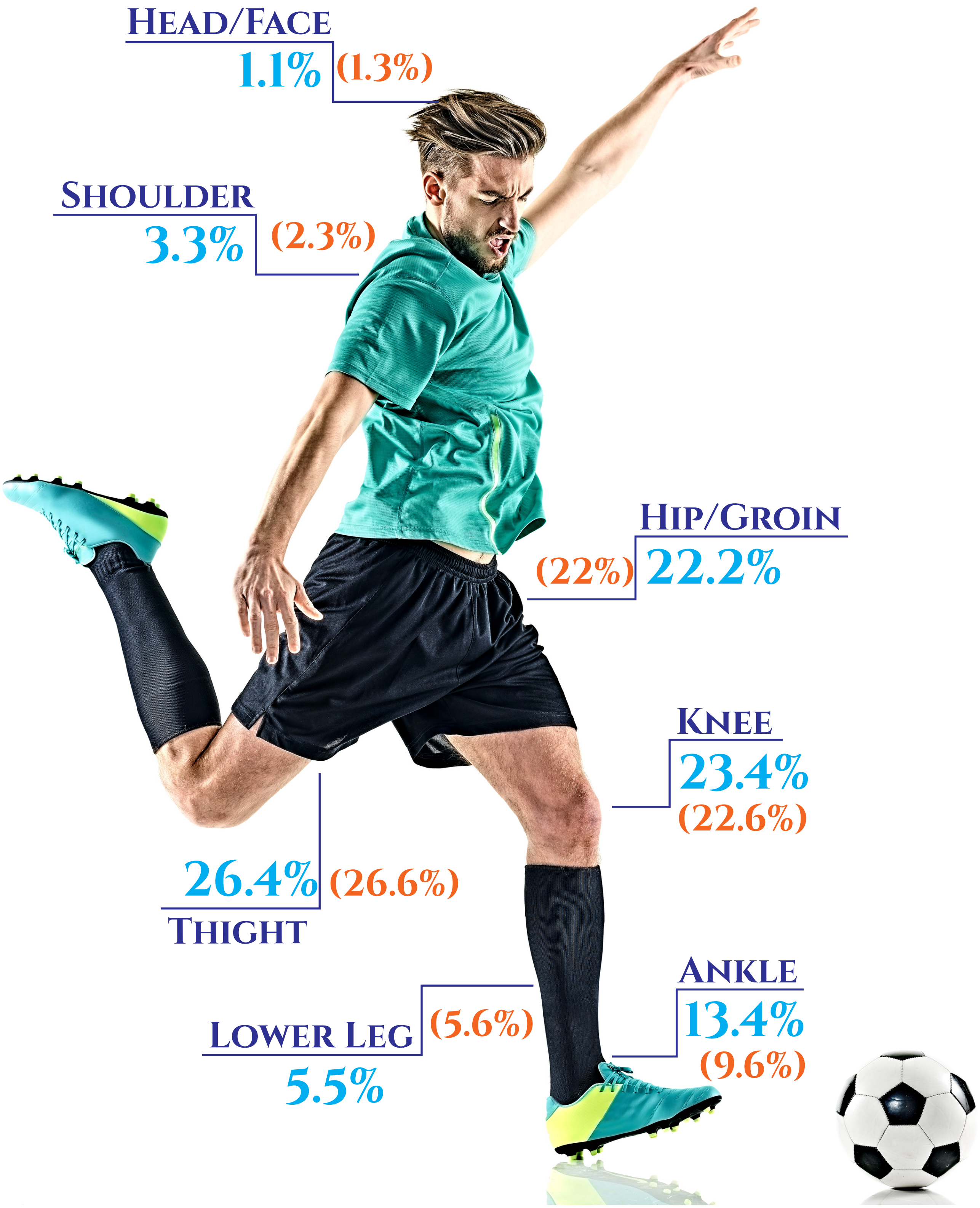

MUSCLE TOTAL INJURY RATE

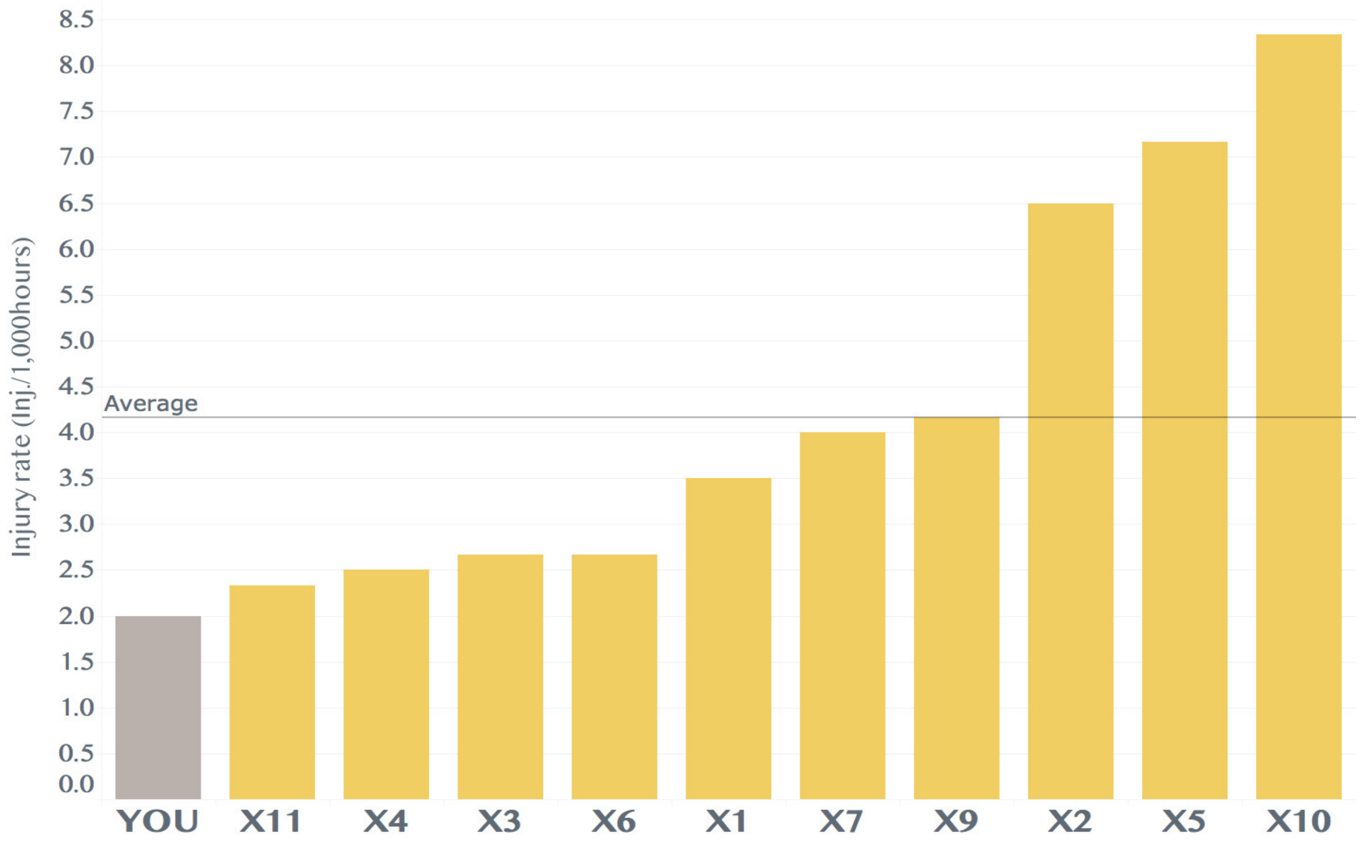

MUSCLE TOTAL INJURY BURDEN

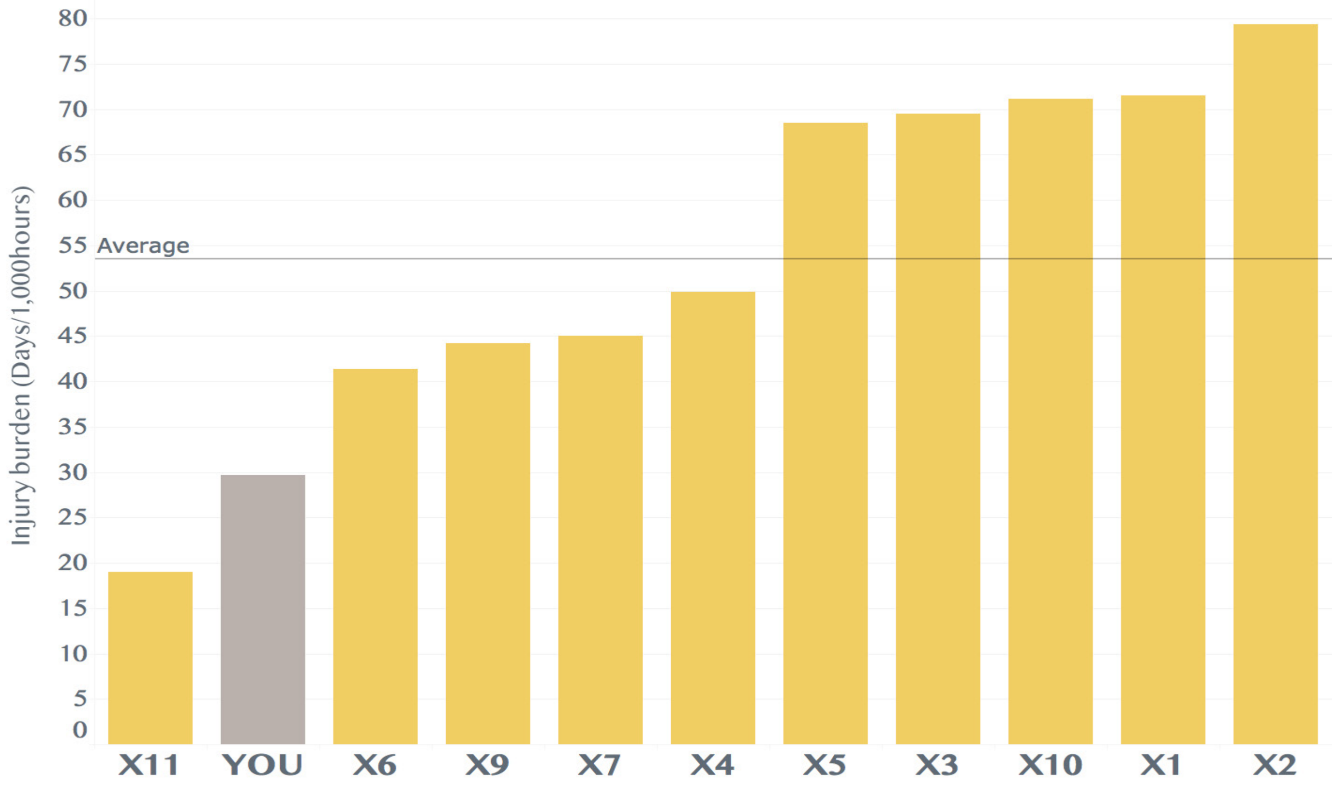

MUSCLE TRAINING INJURY RATE

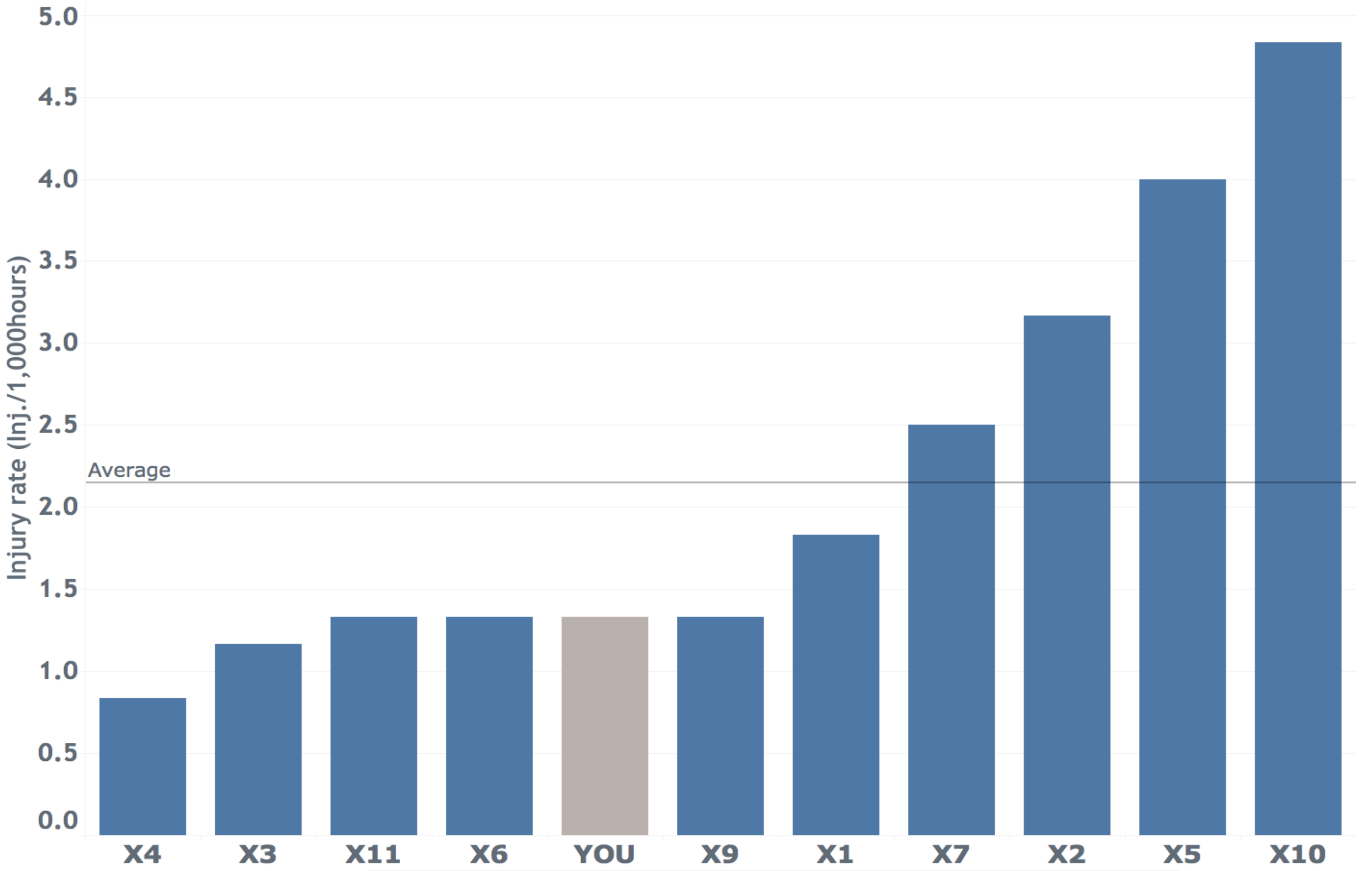

MUSCLE TRAINING INJURY BURDEN

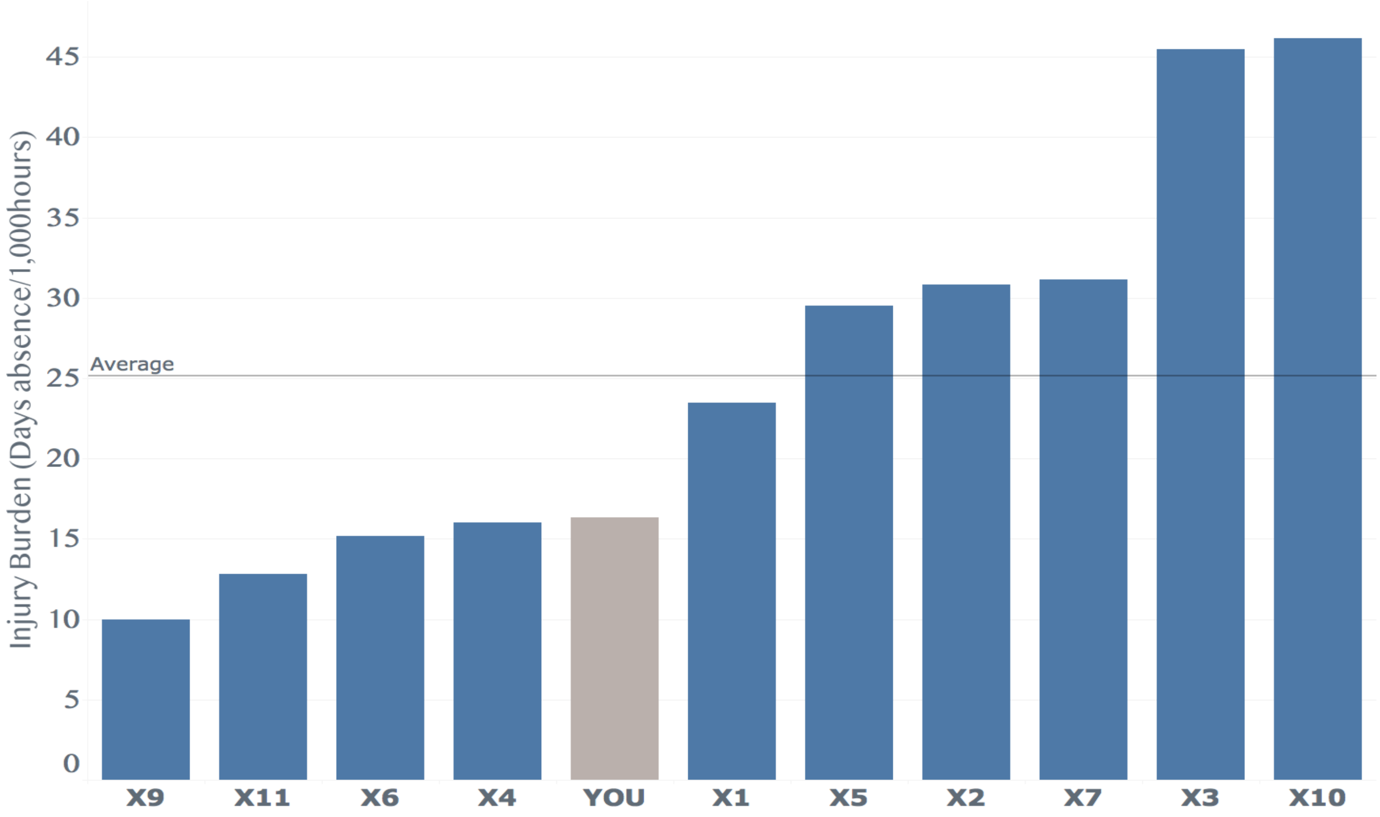

MUSCLE MATCH INJURY RATE

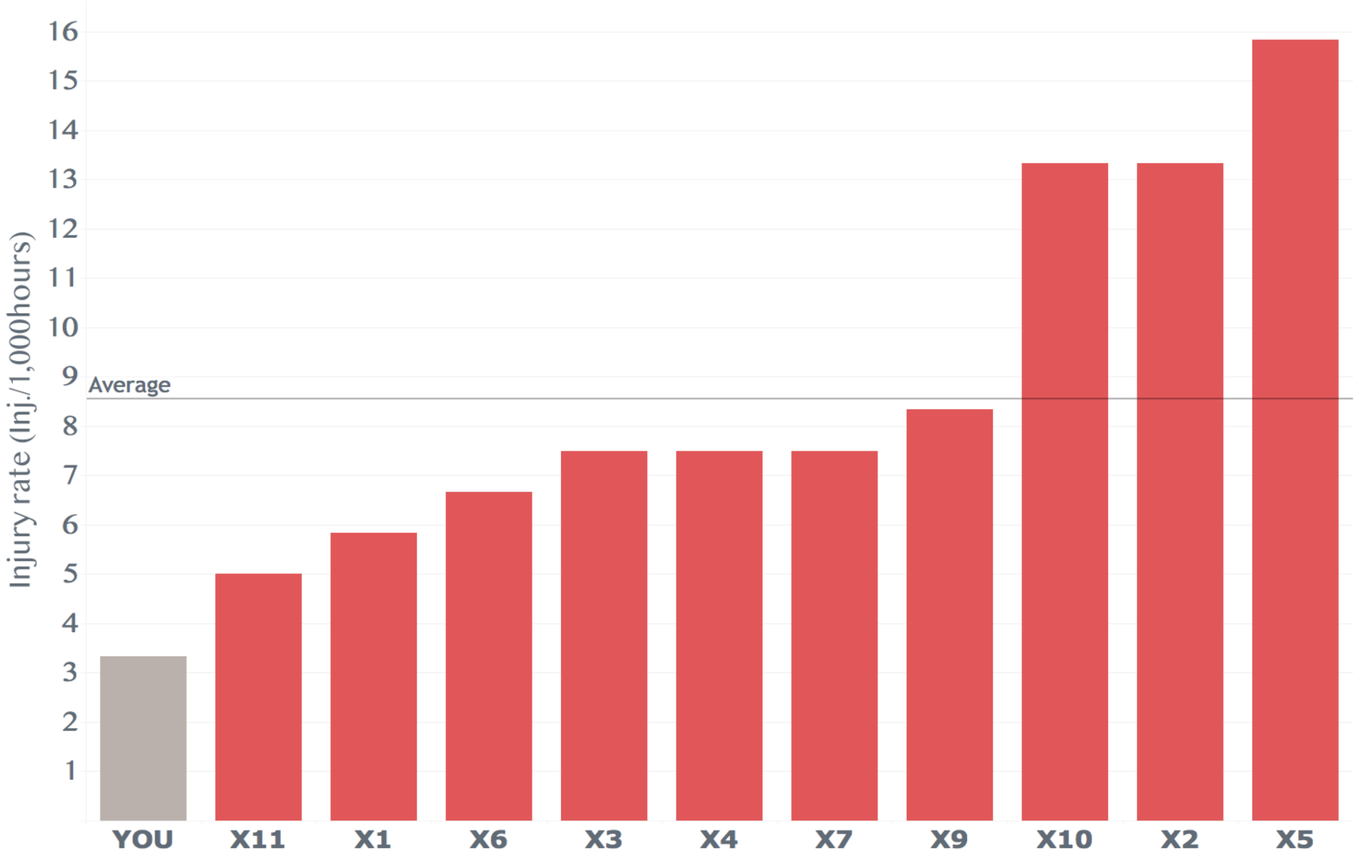

MUSCLE MATCH INJURY BURDEN

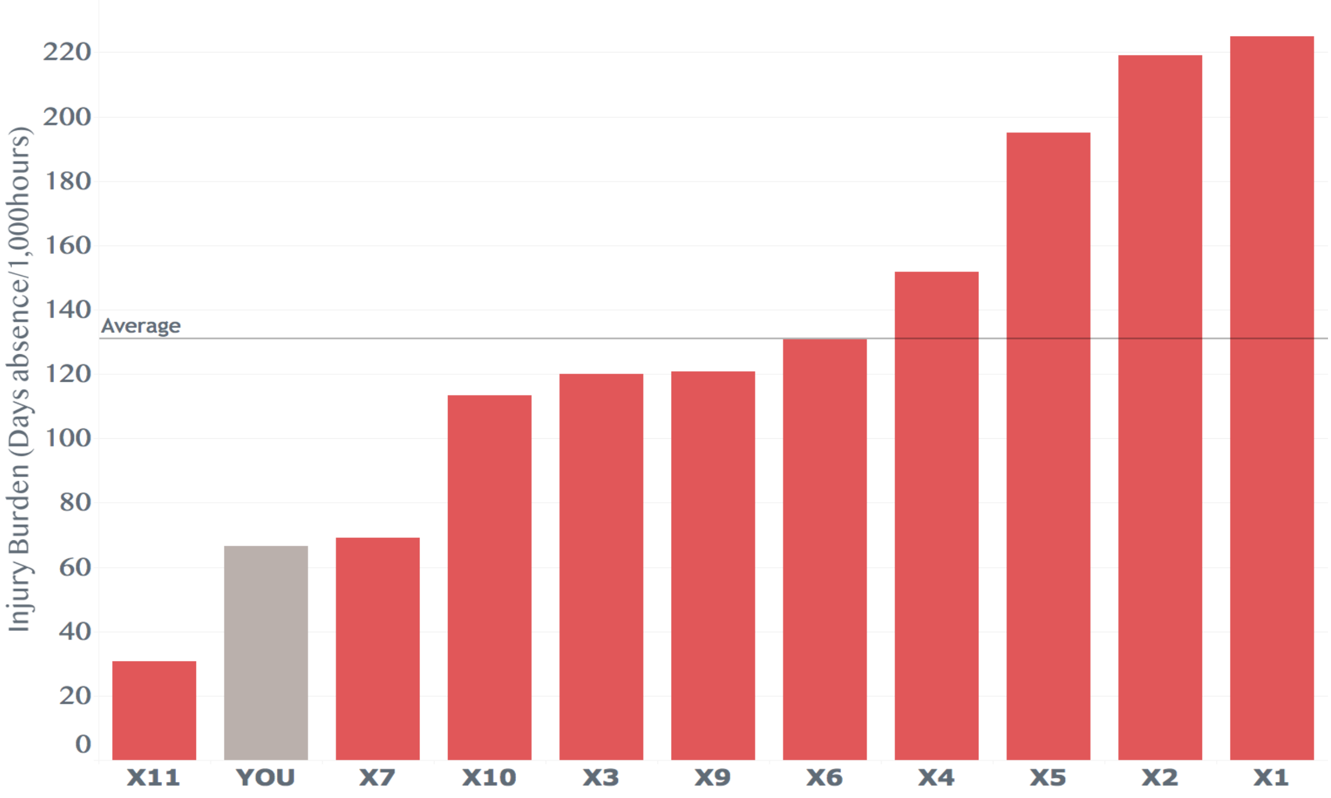

MUSCLE INJURY RATE BY MECHANISM

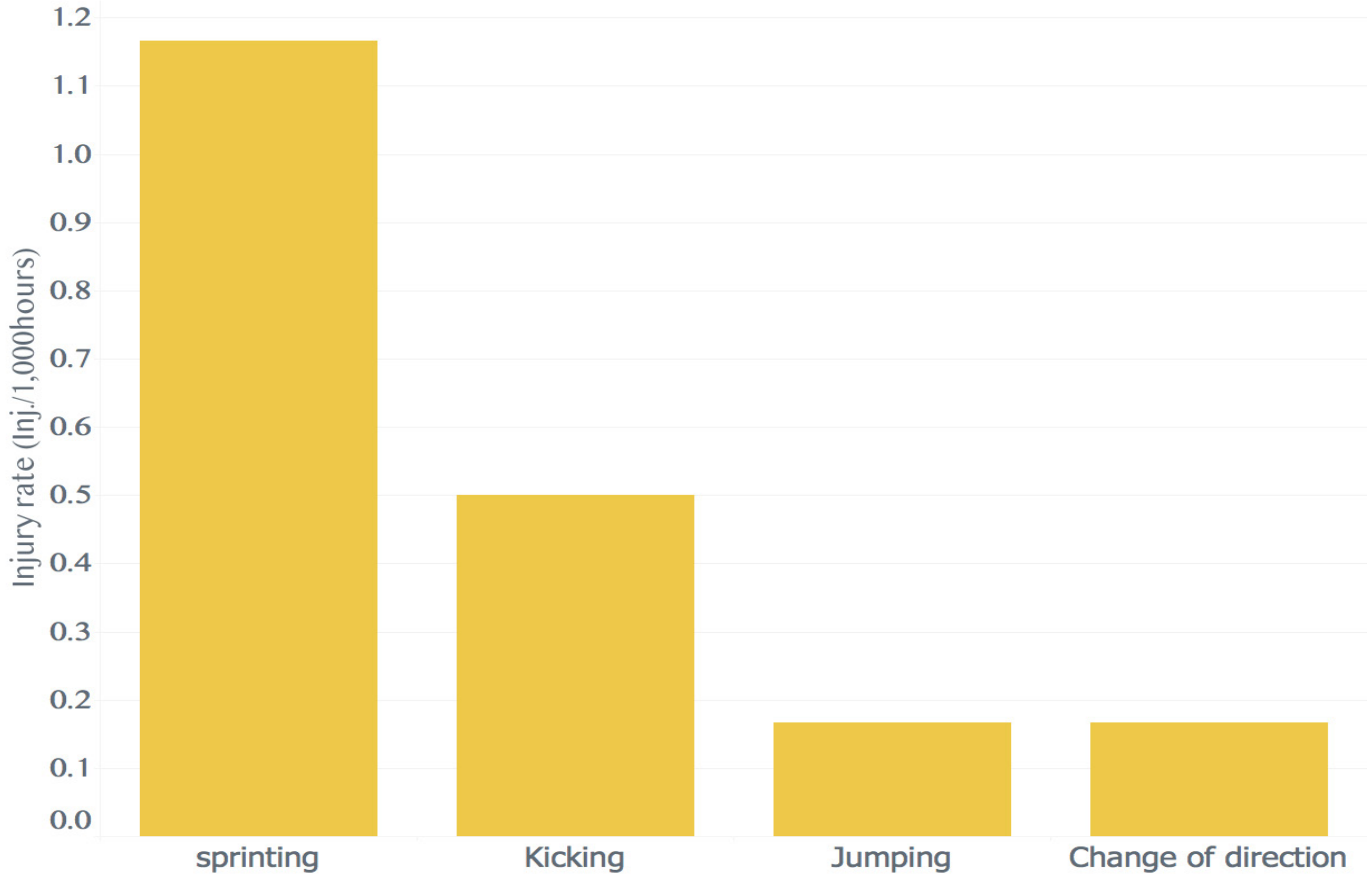

MUSCLE INJURY BURDEN BY MECHANISM

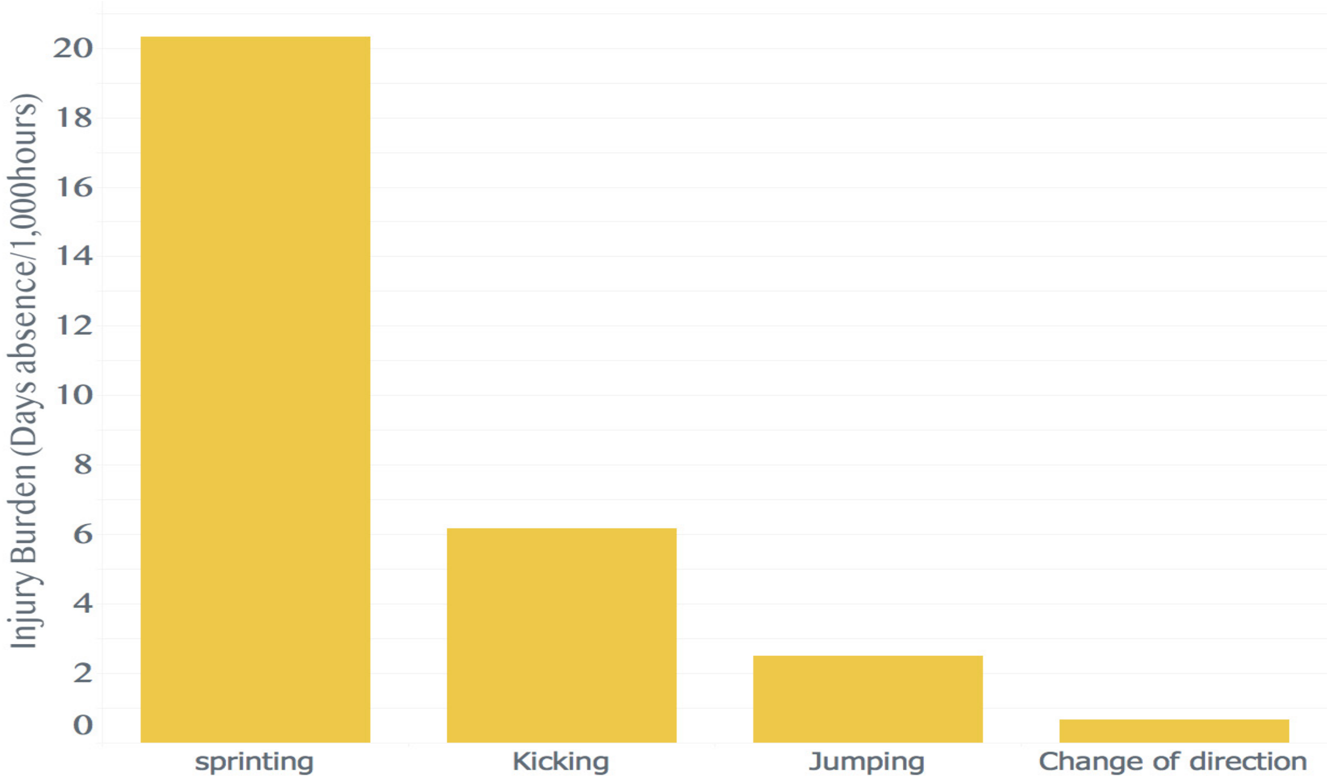

ILNESS RATE

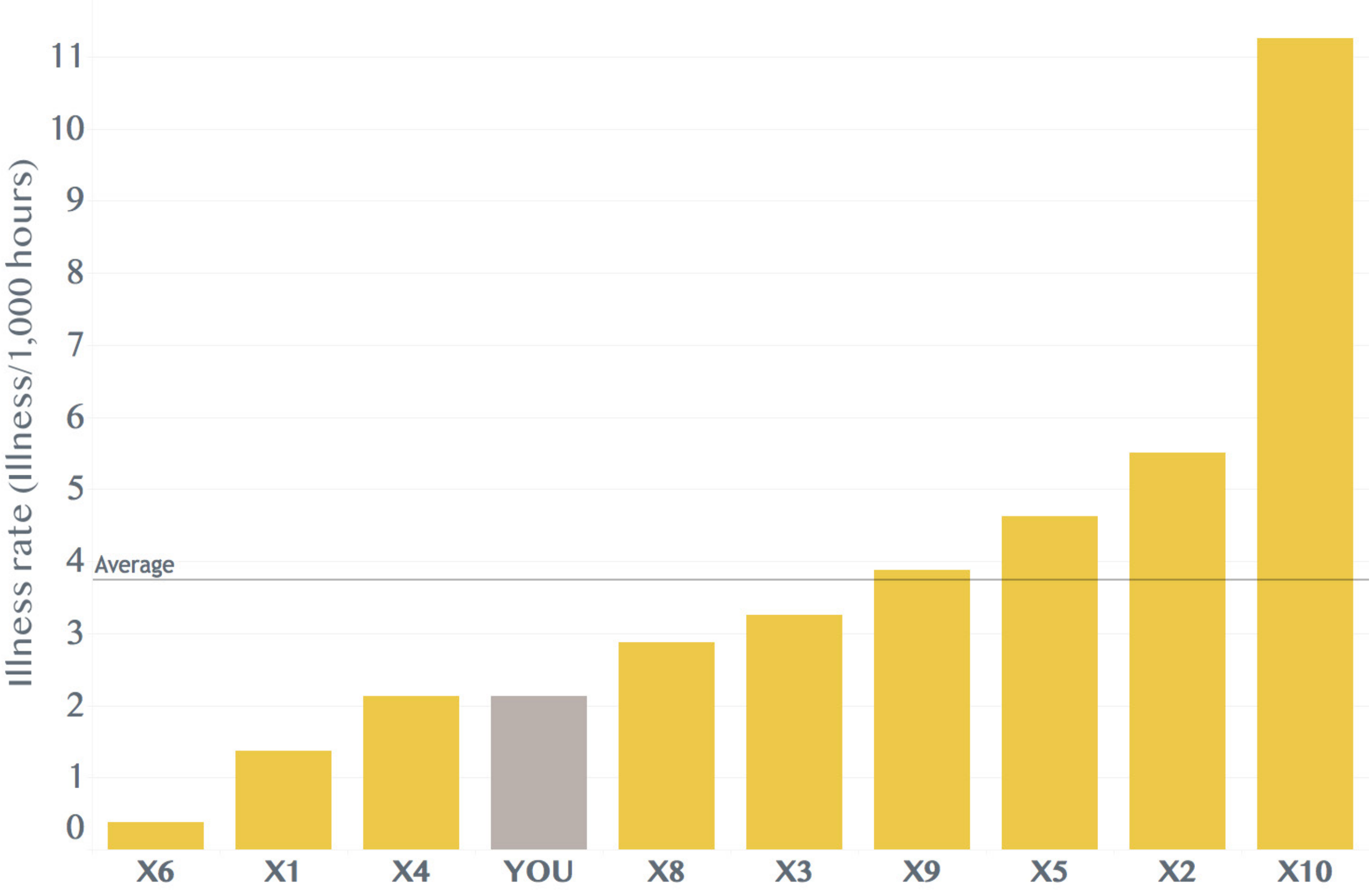

ILLNSS RATE BY AFFECTED SYSTEM

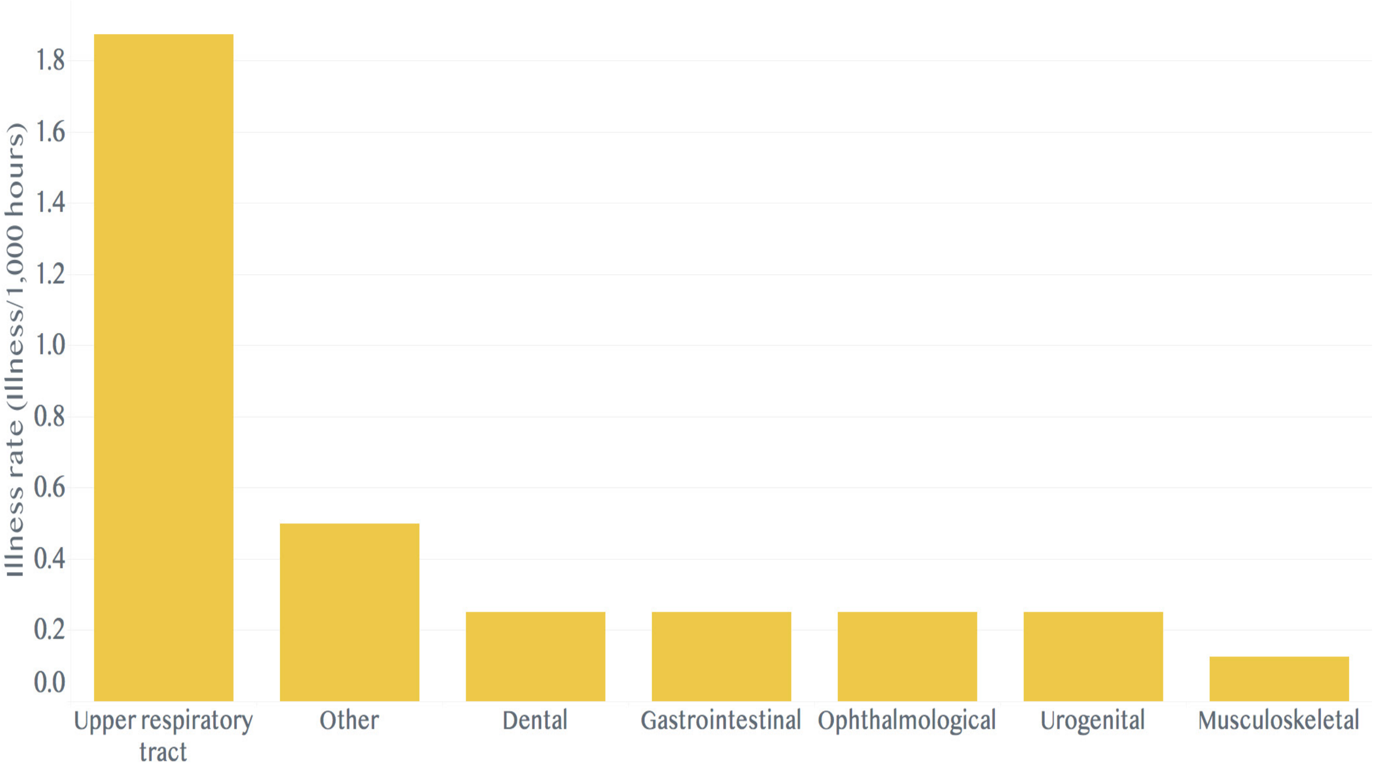

AVAILABILITY
(Excluding National Team Absence)

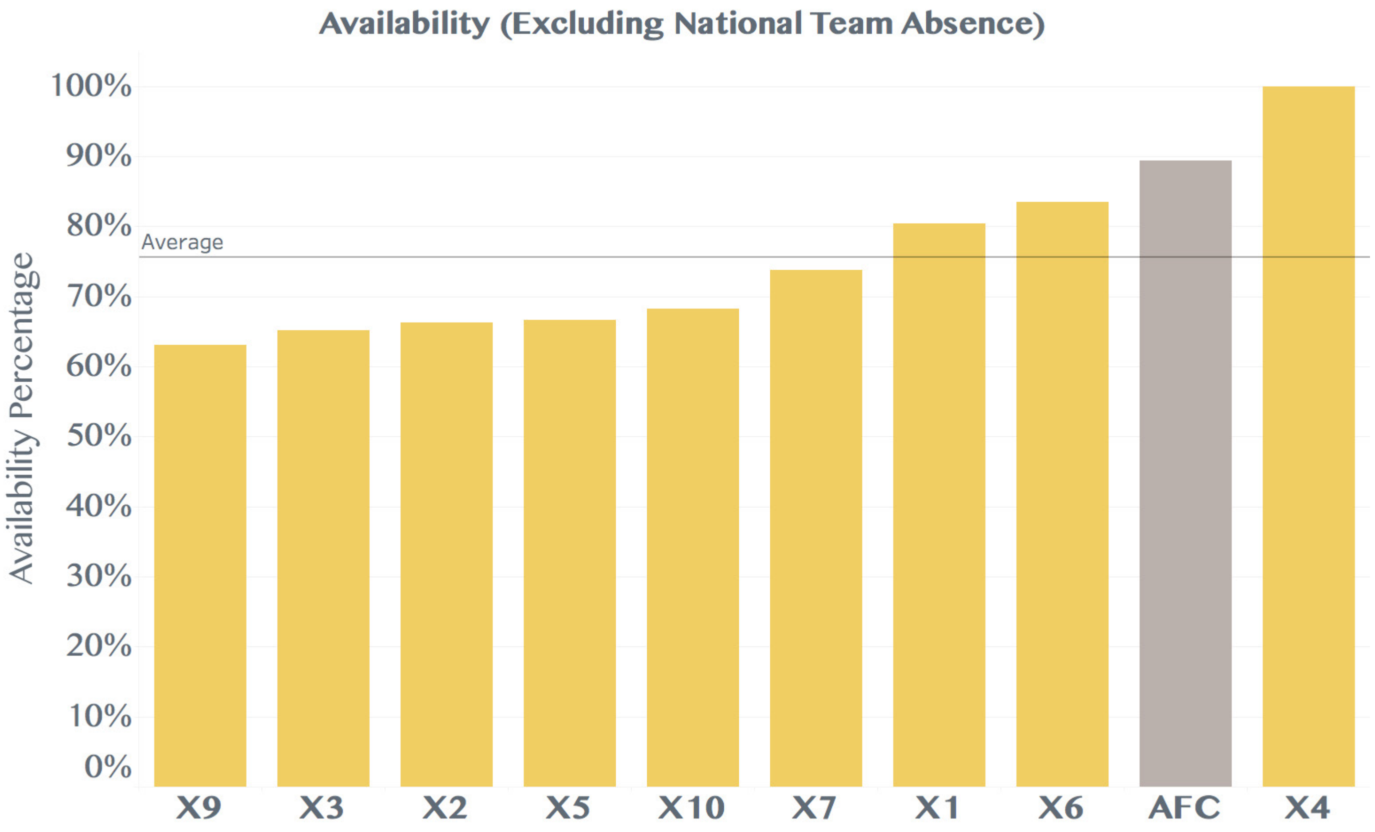

Supplement: Supplementary file 4 — Appendix 3: Dummy Report Example (PDF 12190 KB) [file 40279_2025_2276_MOESM4_ESM.pdf]
