# Supplementary material for: Lessons Learned from Implementing Injury and Illness Surveillance in Professional Football: Introducing a New Implementation Framework
Source: Sports Med. 2025 Jul 11;55(10):2375–85. doi: 10.1007/s40279-025-02276-5 (PMC12513881; doi:10.1007/s40279-025-02276-5)
Supplement: Supplementary file 5 — Appendix 4: AFC Surveillance Manual (PDF 1264 KB) [file 40279_2025_2276_MOESM5_ESM.pdf]

Asian Football Confederation (AFC) Champions League Injury and Illness Surveillance Program

Study Manual

Aspetar Sport Injury and Illness Prevention Program (ASPREV)

June 2016 version

Study group contact persons

Please, in case of doubts and questions, contact:

Dr Cristiano Eirale
29222 Aspetar
Sport City
Doha (Qatar)
Tel +974 4132696
Mob +974 5866032
Fax + 974 4132040
cristiano.eirale@aspetar.com

Prof. Karim Chamari
29222 Aspetar
Sport City
Doha (Qatar)
Tel +974 4413 2725
Mob +974 3318 6096
Fax + 974 4413 2020
karim.chamari@aspetar.com

Mrs. Rima Tabanji
29222 Aspetar
Sport City
Doha, Qatar
Tel +974 44132570
Fax + 974 4413 2020
rima.tabanji@aspetar.com

Contents

Page

1. Definitions 5
1.1 Training exposure
1.2 Match exposure
1.3 Injury
1.4 Illness
1.5 Rehabilitation
1.6 Date of Injury/Illness
1.7 Date of Return to Full Participation
1.8 Re-injury
1.9 Exacerbation
1.10 Onset of injury
1.11 Injury occurring during Match or Training

2. Who is responsible and which players should be included? 9
2.1 Who should be the club's contact person?
2.2 How many players from each club should be included in the surveillance program?
2.3 What about changes in the first team squad?
2.4 What should be done when a player join his National Team for matches-training camps?

3. How to fill in the "Exposure file" 10
3.1 – How to introduce Players in the list.
3.2.1 – Session Exposure
3.2.2 – Players' Exposure
3.2.3 What if the coach decides that a player should not participate in a training session?

4. How to fill-in the "Injury Card" or "Illness Card" form 19
4.1 Specific injury cards
4.2 Groin Card

6. Reports Sheets 24
Description and content

5. Links between XL files 26

7. Sending the forms to Aspetar (Study Group) 28
7.1 How to send the Files?
7.2 When to send the forms?
7.3 Confidentiality
7.4 Questions

Introduction

The prevention of injuries and illnesses relies upon a sound Risk Management Plan for each athlete/team of athletes. The first step in developing a Risk Management Plan is to identify the injury/illness risks for a given sport/group of athletes. Once the Risk has been identified, then prevention programs can be developed targeting the most important problems in terms of frequency and severity.

Therefore, the Aspetar Orthopaedic and Sports Medicine Hospital, with the approval of the AFC, has developed the "Asian Football Confederation (AFC) Champions League Injury and Illness Surveillance Program" to identify specific risks to the clubs participating in the AFC Champions League.

To be able to draw meaningful conclusions from the results of this surveillance program, it is vital that all participating Clubs collect data in a uniform way. This program follows international definitions and methodology used by e.g. FIFA and IOC and Aspetar guidelines.

It is recommended that any Medical staff involved with this program reads this booklet. These guidelines set the basis of the Surveillance Program by setting important definitions and data collection procedures.

We hope you will enjoy reading it and we would be happy to receive your feedback for its continuous improvement. Please don't hesitate to contact us for any doubts or question you may have.

1. Definitions

1.1 Training exposure

Training exposure is defined as any team-based or individual physical activities under the control or guidance of the team's coaching and/or fitness staff that are aimed at maintaining or improving players' skills or physical condition/recovery.

Examples:

1) recovery session with water training after a match is considered as a training session
2) Pre-match warm-up and post-match cool-down sessions should be recorded as "training" exposure. If ever an injury occurs in these pre- or post-match sessions, please record it as a "training" injury.

Personal training and rehabilitation or prevention activities undertaken by players away from the team and not planned and or supervised by the team's coaching or fitness staff will not be recorded as training exposure.

1.2 Match exposure

Match exposure is defined as playing during a competitive or friendly play match between teams from different clubs.

Examples:

1) A "training match" between "yellow" and "green" players of the same team is considered as training and NOT "Match activity".
2) Friendly games played against "another Club", (usually with the presence of an official referee) are considered as "Match activity"

<u>Note:</u> this includes match exposure for the first team players who also sometimes play for teams other than the first team, for example the club's reserve team or national teams. Therefore, for example, when a first player plays a match with the reserve team, the match exposure (together with eventual injuries) should be recorded.

1.3 Injury

A **"time-loss"** definition of injury is used:

An injury is any physical damage that occurs during Training or a Match and results in the player being unable to fully participate in one or more training or match-play sessions.

Injuries that **CAUSE ABSENCE** (even only for a part of a match and training session) should be recorded.
PLEASE RECORD THEM.

Injuries that **DO NOT CAUSE ABSENCE** from football activity do not count. **PLEASE DON'T RECORD THEM**.
Note: a slight injury (for instance, skin abrasion that is been treated for few minutes on the side of the pitch with the player resuming training/play) does not count.

Injuries that occur **OUTSIDE FOOTBALL ACTIVITIES** (training and matches) do not count. These are injuries requiring "medical attention and care", but as long as they did not occur during football activity (match or training), they should not be recorded in the files. **PLEASE DON'T RECORD THEM**. The Doctor will thus keep the "injury cards" separately from the other injuries.
If they cause absence from football, the player should be considered "absent for other reasons" and the exposure sheet filled with the symbol A (see XL section further).

IMPORTANT: for each injury, specific questions will be asked (please refer to the Excel file XL injuries-illnesses and to the injury cards)

1.4 Illness

A "**time-loss**" definition of Illness is used:

An illness is a physical or psychological complaint or manifestation experienced by a player that occurs at any time (during or out of football activities) and results in the player being unable to fully participate in one or more training or match-play sessions.

Illnesses or complaints that **CAUSE ABSENCE** (even only for a part of a match and training session) **OCCURRING AT ANY TIME** should be recorded.
PLEASE RECORD THEM.

IMPORTANT: for each illness, specific questions will be asked (please refer to the Excel file XL injuries-illnesses and to the injury cards)

1.5 Rehabilitation

The "Rehabilitation period", is the full period comprised between the first day of activity resumption on the field/clinics (after the full stop due to injury/illness), to the last session of rehabilitation/training before "Return to full participation"

A player is classed as injured or ill for as long as he cannot participate in any type of football activity. A player is considered fully rehabilitated when the team doctor/physiotherapist declares him fit for full participation to training AND matches.

Note: if any part of a training session is modified for a player due to an existing injury this is considered part of the rehabilitation programme for that player and should not be considered as training.

1.6 Date of Injury/Illness

The date of injury/illness is set when the athlete stops football activity (training OR matches) **because of this injury/illness** (NOT WHEN THE SYMPTOMS STARTED and INDEPENDENTLY OF THE DATE OF CONSULTATION with the medical staff).

1.7 Date of Return to Full Participation

This is the date corresponding to the first day of "full/unrestricted" participation to football activity (training or matches).

The date of return to play cannot be the same as the day of injury/illness (otherwise there will not be at least one day of time-loss).
The information of this date has to be entered in the XL file in order to fully complete the injury/illness Card (whenever no date of "Return to full participation" is entered, the column with "Special attention" will stay "Red" as a reminder to enter this date to close the Card).
Please note that the choice of the coach to use the player or not for any game has no influence on this date.

1.8 Re-injury

"Re-injury" is defined as an injury OF THE SAME TYPE AND AT THE SAME ANATOMICAL LOCATION which occurs after a player's "Return to full participation" from the previous injury occurring WITHIN 1 YEAR.

A "re-injury" has to be recorded as a NEW injury case/card with the previous "original injury" file closed with a clear "Return to full participation" date.

Note: When recording the re-injury period you have options ranging from within "one week" to within "12 months".

1.9 Exacerbation

An Exacerbation occurs when time-loss from Rehabilitation occurs to a player who has not been yet in "full participation" after an injury.

The injury shall be classified as an Exacerbation where the "original injury" worsens to the point where the player has to stop his rehabilitation/training activity for at least one session.
Obviously, if the player has been previously declared fully fit to participate from a previous injury, the injury shall be recorded as a "Re-injury" and not as "Exacerbation" (see section 1.8).

For any "Exacerbation injury", a NEW file/Card (and date of injury) has to be set, with the previous "original injury" file NOT yet closed by a "Return to full participation date".

The only exception lies when a player joins the club with an "already existing complain". In this case, the Doctor does not have the "original injury file", and thus any eventual "exacerbation" will not be preceded by an "original injury" file.

<u>Example</u>: the player had an injury and is regularly performing his rehabilitation. The medical staff is happy with the evolution and decides to test the player in full training before clearing him for a 'Date of return to full participation" (full training + competition). If an injury occurs at that moment of "testing" it has to be classified as exacerbation, since this specific training was a testing and not a regular training occurring after "date of return to full participation".

1.10 Onset of injury

Injuries should be classified as "**Sudden**" or "**Gradual**", according to their onset.

SUDDEN ONSET: the injury is resulting from a specific incident clearly recalled by the player.

GRADUAL ONSET: the injury developed progressively over time (days/weeks/months) and a specific incident or moment in which the injury occurred CANNOT be recalled by the player.

1.11 Injury occurring during Match or Training

If a "sudden onset" injury is experienced, please mention if the injury occurred during "**Match**" or "**Training**" and also mention the corresponding timing of the injury (at which moment it occurred during the corresponding match or training session).

N/A (Not Applicable) applies to the cases of "Gradual Onset" injuries, where it is not possible to state if the injury occurred during Match or Training.

2. Who is responsible for the surveillance and which players should be included?

2.1 Who should be the club's contact person?

The contact person will be responsible for collecting the data and delivering them to the study group. We suggest the Team Doctor (or Head Physiotherapist if there is no doctor available) as contact person.

2.2 How many players from each club should be included in the surveillance program?

All players in the first team squad should be included in the study. The exposure file allows entering 50 players however, we suggest that approximately, 25 players per team are included in the surveillance program. It is worth noting, that during the preparation phase of the season, in some clubs the number of players can exceed 30 players. The injuries/illnesses and exposure of all these players should be noted.

Any player being involved with the club for more than a few days (testing days, for example), should be monitored. Once the player has successfully passed any such testing (eg. 2-3 days) please include him in the list of the surveillance program. If such a player leaves the club within a 2 week period, then delete/exclude him from the data archived. Any player having spent 2 weeks or more with the club should be included in the data collection and analysis.

2.3 What about changes in the first team squad?

Players leaving the team during the season
Players who leave the club during the season SHOULD BE EXCLUDED FROM THEIR DATE OF LEAVING.
Note: if a player is injured and then leaves the club (or the end of season off-period occurs), then the contact person should fill an injury card for that injury and *estimate the date of return to full participation.*

Players joining the team during the season
Players who join the team during the season SHOULD BE INCLUDED FROM THEIR DATE OF JOINING.

Players training regularly with the first team
Any player who continuously train with the first team, but sometimes go and play with the second/Reserve/U23 team, SHOULD BE INCLUDED IN THE SQUAD.
Even when not training or playing with the First team, but with the second/Reserve/U23 team, their training and match exposure should be recorded.
If their training or match exposure cannot be obtained, they should be noted as "Absent" (for other reasons than injured/illness).

3. How to fill in the "Exposure file"

Important!

Please note that the files are "Simple Excel (XL)" files, with many cells containing formulae that allow the file to function.
For instance, in the figure-1, the cursor has been placed on: Cell H-12 containing the formula shown in fx: =IF(OR(E12="";G12="");"";DV!E2). ***Please <u>do not press DELETE</u> when your cursor is on any Cell containing a formula.***
Also, please do not go to *fx* and modify it. This would either introduce an error in the file or impede the function related to this cell to work properly.

Microsoft Office Excel versions prior to 2010 are not advised for use, due to functionalities issues.

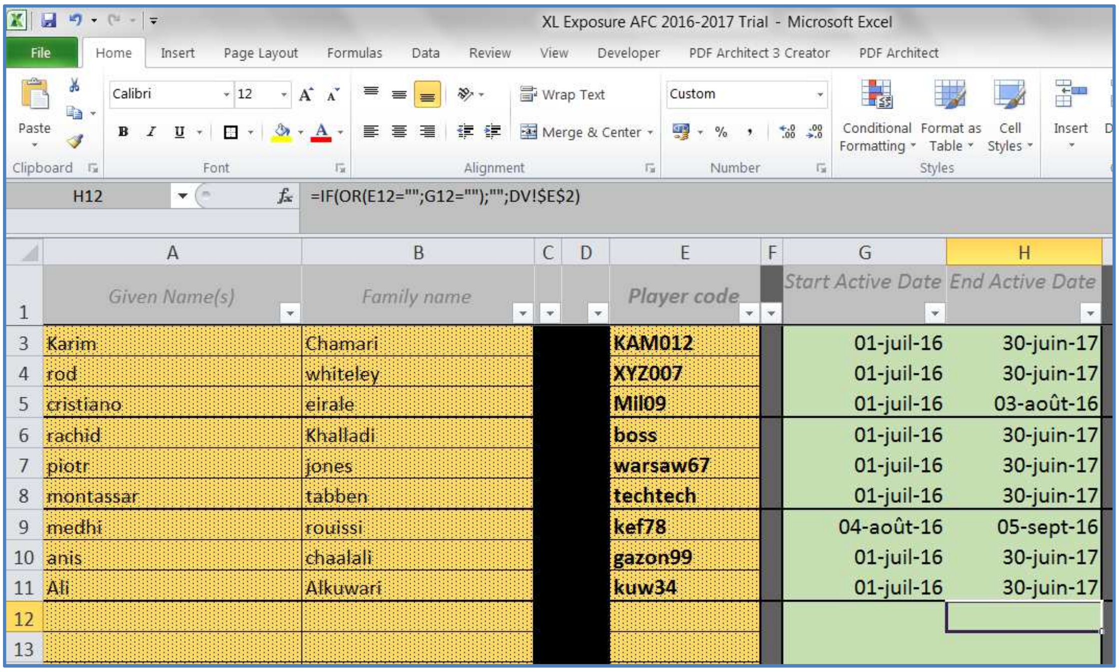

	A	B	C	D	E	F	G	H
1	Given Name(s)	Family name			Player code		Start Active Date	End Active Date
3	Karim	Chamari			KAM012		01-juil-16	30-juin-17
4	rod	whiteley			XYZ007		01-juil-16	30-juin-17
5	cristiano	eirale			Mil09		01-juil-16	03-août-16
6	rachid	Khalladi			boss		01-juil-16	30-juin-17
7	piotr	jones			warsaw67		01-juil-16	30-juin-17
8	montassar	tabben			techtech		01-juil-16	30-juin-17
9	medhi	rouissi			kef78		04-août-16	05-sept-16
10	anis	chaalali			gazon99		01-juil-16	30-juin-17
11	Ali	Alkuwari			kuw34		01-juil-16	30-juin-17
12								
13								

<u>**Figure 1:** "Exposure Sheet" file. Sheet "Players-List".</u>

3.1 – How to Introduce Players in the List

a/ Order of entry:

Enter first, the players who are in contract with the club.
Thereafter, enter the players who are in the Reserve Team.
Lastly enter the players who are "under testing" or at risk of leaving the group.
This will allow having the most active players on the top of the list.
<u>Please note that it is not possible to change the order of the player list in the XL file during the season.</u>

b/ Entry of a new player in the list:

For the identity of the player, please enter only A, B, E (columns) information (Given name(s), Family Name, and CODE (see below) – Figure 1.

Please enter this information in the "Exposure XL file". The 'Player list" is always entered and modified in this file. The list in the "Injuries/Illness XL file" is simply transferred from this file by a simple copy/paste. When exporting the list of players from the "Exposure" file to the "Injury/Illness" File, please only export columns A to E (Players Given Name to Players' code column).

	A	B	C	D	E	F	G	H	I	J	K	L	M	N	O	P	Q	R	S
1	Given Name(s)	Family name			Player cod		Start Active Da	nd Active Date		Start Active Dat	d Active Dat	is Active?	Today:	11-Jun-16	Age (years)	Weight (kg)	Height (cm)	Main Position	Dominant leg
2																			
3	Karim	Chamari			KAM012		01-juil-16	30-juin-17				FALSE			27	70	171	Goal Keeper	Left
4	rod	whitel			XYZ007		01-juil-16	30-juin-17				FALSE			32	89	187	Lateral Defender	Right
5	cristiano	eirale			Mil09		01-juil-16	03-août-16				FALSE			22	92	190	Central Midfielder	Right
6	rachid	bouras			boss		01-juil-16	30-juin-17				FALSE			19	90	195	Lateral Mid fielder	Right
7	piotr	jones			warsaw67		01-juil-16	30-juin-17				FALSE			23	78	175	Central Defender	Right
8	montassar	tabben			techtech		01-juil-16	30-juin-17				FALSE			24	85	187	Central Striker	Right
9	medhi	rouissi			kef78		04-août-16	05-sept-16		05/01/2017	30-Jun-17	FALSE			27	80	183	Lateral Mid fielder	Right
10	anis	chaalali			gazon99		01-juil-16	30-juin-17				FALSE			23	76	179	Lateral Forward	Left
11	Ali	Al Kuwari			90876		01-juil-16	30-juin-17				FALSE			21	84	188	Lateral Defender	Right

Fig 2: Players' data in the "Exposure XL Sheet".

Basic information about the players (Fig 2, right section):

On the right of the "Players' List" sheet in the "Exposure XL" file you can enter basic information about the players (Age in years; Body mass (weight) in kg; Body height in cm).

Player's Main Position:
This corresponds to the player' preferred position. If the coach uses the player in another position for tactical reasons, this does not have to impact on this option. Usually the player knows his "preferred position" and normally it is the position in which he plays the majority of the matches.
You can chose between the following options:

- Goalkeeper
- Central defender
- Lateral defender
- Central midfielder
- Lateral midfielder
- Central striker
- Lateral striker

Dominant leg:
Simply ask the player which is his dominant leg, i.e. the leg with which he predominantly shoots and passes the ball. If the player says he is ambidextrous, please ask him the following question: "If you are about to shoot a decisive penalty kick in an important official game, with which leg would you shoot the ball?". The answer should provide the information about his dominant leg.

Players' basic information should be collected at the beginning of the season (or as soon as the player joins the team). If for instance, body mass changes during the season, please do not enter the new body mass in this basic information section.

Player' Code

Each Team in the Study will have to allocate an individual AND UNIQUE code for each player. If ever this specific player leaves the club, this code has not to be used anymore for any other player, to avoid any mixing-up of player's identities. This code will allow the clubs, to anonymize their files before sending them to the Study Group at Aspetar Hospital.
It is important to note that the Code is freely chosen by the Club's Doctor, who will be the only one knowing to which player corresponds a certain code.

Practical tip:
We suggest that the Doctor uses letters and digits that could help her/him easily recognize the identity of the player when working on the sheets.
If on the Player's-List sheet, the identity is displayed with the name/family name AND code, **when inserting data** (exposure, injuries, and illnesses) **only the players' code will be displayed** but please understand that the players' names are NOT listed. Therefore, if the user chooses a "friendly code" this will allow an easier way of working with the spreadsheet on a daily basis.
If the Doctor/physiotherapist in charge of data collection chooses to use a complex code, he can easily check the identity-code corresponding on the "Players' list" Sheet.

<u>**VERY IMPORTANT**</u>: Once codes have been allocated to a player, you should print-out the list of codes/identities and keep them locked in a safe place as a backup. If ever an error of manipulation happens on the XL files, the Doctor will be able to determine which code corresponds to which player.
Please note that this printed list will need to be updated (printed-out) during the season, whenever a new player joins the team.

Files Anonymization.

<u>Before sending the file to the Aspetar study group, the identity of the players need to be anonymized. For anonymizing the file please:</u>

1- Open the files on XL.
2- Select "Save as" and save the files with the same name but by adding "Anonym" at the end of the file's name.
3- "Save the new "anonym" file.
4- Now on this "Anonym" File, delete (by the command clear contents) the players' names and family names (not the codes !) (Fig. 3). At the moment of deleting the Names please make sure you are on the "Anonymized" file.
5- Save the file on which the names have been deleted (please make sure the codes are still on the anonymized file).
6- Send the file to the study group.

Afterwards, please do NOT work on the anonymous file but continue your injury/illness data surveillance on the regular file adding further data on the "original file". <u>Anonymized files are only created at the moment of sending the files to the study group.</u>

At the end of each month when you send anonymous data to the Aspetar study group, please repeat the steps described here above on both XL files (the "Exposure" and the "Injury/Illness" files).

When doing this for the second time (and all consecutive times), a message will pop-up at the moment of saving (a file with the same name already exist, do you want to over-write it?). Please do so, overwrite the older version of the "anonymous" file and then, delete Again the names (leaving the codes) and send it to the study group.

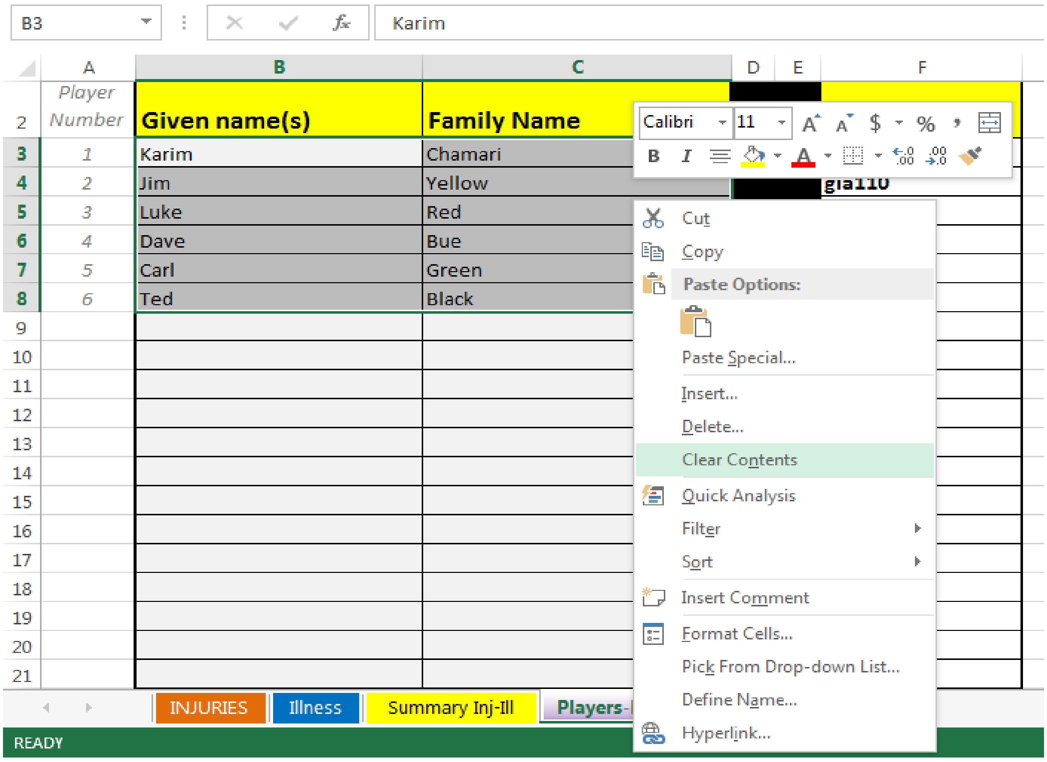

<u>**Fig 3.:** how to delete the names from the anonymized file. Select the names and family names (without selecting the codes) and delete them through the right click and selecting "clear contents" (please read the text for the process of anonymization of the files. Please note that on fig 3, only 2 columns of the "Given names" and "Family name" are highlighted for deletion.</u>

Activity Dates

These dates are set on the XL of the exposure sheet to facilitate the collection of the data.

When you enter a player, the automatic dates of "Activity" will be set as "1st July to end of June of the following year".

Please change the dates accordingly to the reality of the activity of each player.

	Given Name(s)	Family name		Player code	Start Active Date	End Active Date	Start Active Date 2	End Active Date 2	is Active?
1									
2									
3	Karim	Chamari	[redacted]	KAM012	1-Jul-16	30-Jun-17			FALSE
4	cristiano	eirale		CEI0010	1-Jul-16	10-Jul-16	1-Sep-16	30-Jun-17	FALSE
5	francesco	camerano		bar010	1-Jul-16	30-Jun-17			FALSE
6	luiso	torcane		Jan009	1-Jul-16	30-Jun-17			FALSE
7	william	wannabe		wan188	30-Mar-17	30-Jun-17			FALSE
8	henry	logan		log118	1-Jul-16	30-Jun-17			FALSE
9	continente	green		asi015	20-Jul-16	30-Jun-17			FALSE
10	emmanuel	atton		ema710	1-Jul-16	30-Jun-17			FALSE
11	siljo	eandiral		raf437	1-Jul-16	30-Jun-17			FALSE
12	warsee	itiella		tit174	1-Jul-16	30-Jun-17			FALSE
13	firm	werghiangi		how123	1-Jul-16	1-Jul-16	20-Jul-16	30-Jul-16	FALSE
14									FALSE
15									FALSE
16									FALSE
17									FALSE
18									FALSE
19									FALSE

Fig 4: Example of Activity dates

For instance, on Fig 4, the player in raw 7 joined the group during the season (for example, this could apply for the players that are bought during the "players' transfer window").
The player in raw 9 joined the group late (around 3 weeks after the start of training resumption).
The player raw 4 trained with the team for around 10 days and then left. He came to join the team again from the first of September to the end of the season.

The XL file allows to have 1 to a maximum of 2 "Activity" periods per player. This will allow the File to automatically declare the player as "Present" for any session within these "Activity" period. For any period not included, the file will NOT consider the player within the team, and then will not set any exposure for him.
The concerned cell will display "inactive", not allowing to enter any exposure.

The "Activity" periods are for important changes of status (player leaving the club or joining the Club) and NOT for absences for injuries (even if very long – please see below).

Note: It is practical to use these "Activity Dates" for players. This will avoid having an "inactive" player (player having left the club, for instance), being allocated a training or match exposure, for every session. This will force the used to go to his cell and remove the exposure. These are unnecessary actions that are boring for the user. By declaring the player "inactive" the XL automatically sets the exposure as "inactive" and the user does not have to care about all "inactive players".

3.2 Managing the Sessions' "Attendance/Exposure"

The "Exposure Sheet" contains 12 sheets corresponding to 1 sheet per month.
Each sheet is called by a number. "1" for the first month of the season. **Please do not change the name of the sheet (leave it as 1)**, as the injuries/illnesses reports do rely on the reference of each sheet by its' name (1) to calculate the report scores.
Each sheet contains as many columns as the number of days in the month.
For each day, there are 5 possibilities of sessions to be entered (1 match, 2 training sessions with the ball and 2 training sessions without the ball, see Fig. 5).

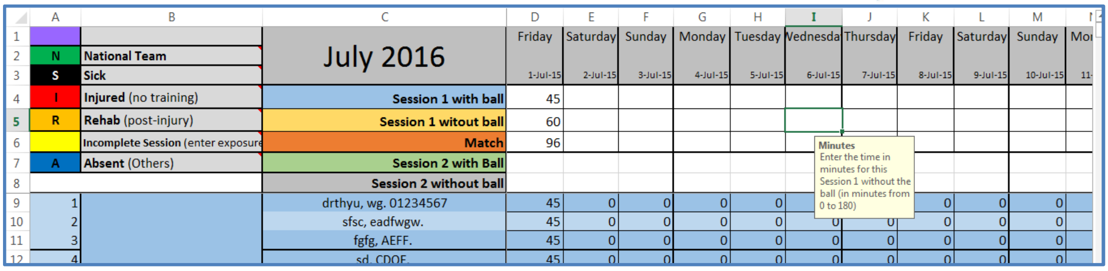

	A	B	C	D	E	F	G	H	I	J	K	L	M
1			July 2016	Friday 1-Jul-15	Saturday 2-Jul-15	Sunday 3-Jul-15	Monday 4-Jul-15	Tuesday 5-Jul-15	Wednesda 6-Jul-15	Thursday 7-Jul-15	Friday 8-Jul-15	Saturday 9-Jul-15	Sunday 10-Jul-15
2	N	National Team											
3	S	Sick											
4	I	Injured (no training)	Session 1 with ball	45									
5	R	Rehab (post-injury)	Session 1 witout ball	60									
6		Incomplete Session (enter exposure	Match	96									
7	A	Absent (Others)	Session 2 with Ball										
8			Session 2 without ball										
9	1		drthyu, wg. 01234567	45	0	0	0	0	[illegible]	[illegible]	0	0	0
10	2		sfsc, eadfwgw.	45	0	0	0	0	0	0	0	0	0
11	3		fgfg, AEFF.	45	0	0	0	0	0	0	0	0	0
12	4		sd, CDOF	45	0	0	0	0	0	0	0	0	0

Minutes
Enter the time in minutes for this Session 1 without the ball (in minutes from 0 to 180)

Figure 5: Monthly ''Exposure/attendance sheet''.

3.2.1 – Session Exposure

If your team performed only one session in a day, please enter the ''Exposure/Duration'' (in minutes) in the appropriate cell.
For instance, if for the 1st of July the team played a match of 98 min, enter ''98'' in the cell D6 (Figure 6).

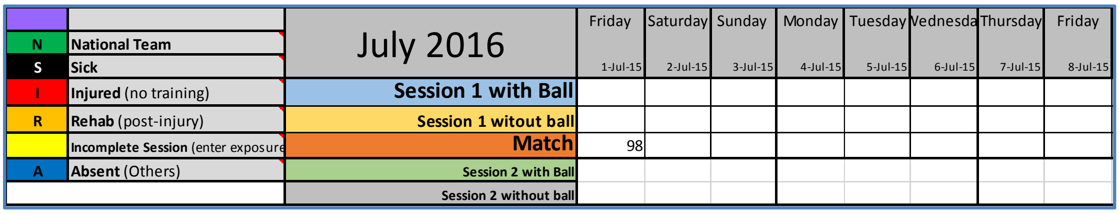

			Friday 1-Jul-15	Saturday 2-Jul-15	Sunday 3-Jul-15	Monday 4-Jul-15	Tuesday 5-Jul-15	Vednesda 6-Jul-15	Thursday 7-Jul-15	Friday 8-Jul-15
N	National Team	July 2016								
S	Sick									
I	Injured (no training)	Session 1 with Ball								
R	Rehab (post-injury)	Session 1 witout ball								
	Incomplete Session (enter exposure	Match	98							
A	Absent (Others)	Session 2 with Ball								
		Session 2 without ball								

Figure 6: example of data entry, match played on the 1st of July

For any exposure entered, the entered exposure will be allocated in the TABLE corresponding to the type of session chosen for all the ''active'' players (Figure 7).

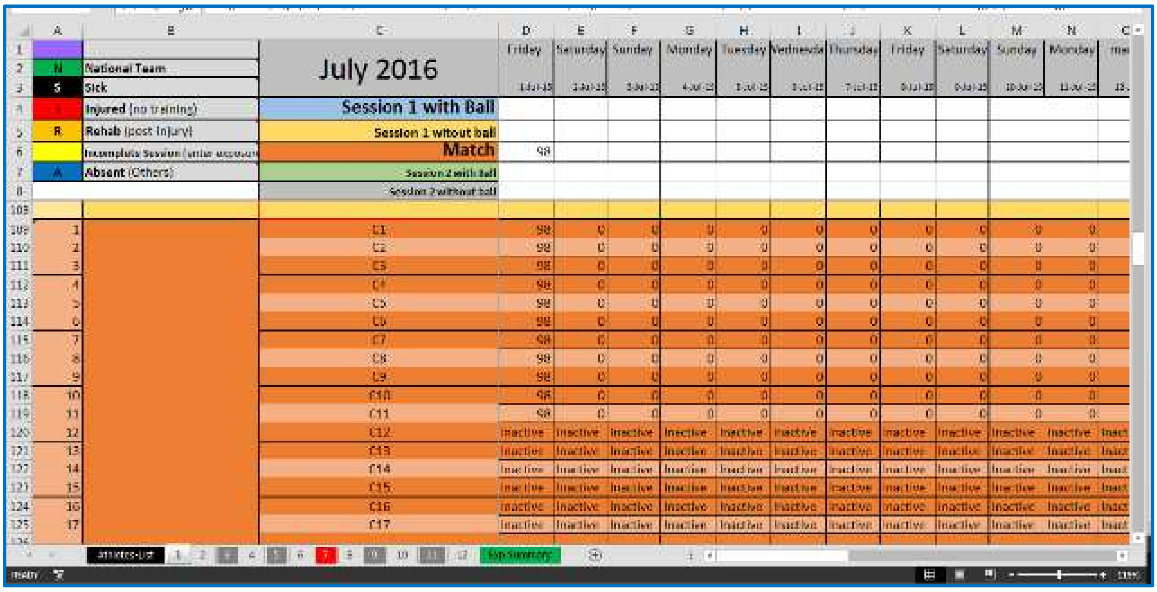

	A	B	C	D	E	F	G	H	I	J	K	L	M	N
1			July 2016	Friday	Saturday	Sunday	Monday	Tuesday	Wednesda	Thursday	Friday	Saturday	Sunday	Monday
2	N	National Team												
3	S	Sick												
4	I	Injured (no training)	Session 1 with Ball											
5	R	Rehab (post-injury)	Session 1 witout ball											
6		Incomplete Session (enter exposure	Match	98										
7	A	Absent (Others)	Session 2 with Ball											
8			Session 2 without ball											
108														
109	1		C1	98	0	0	0	0	0	0	0	0	0	0
110	2		C2	98	0	0	0	0	0	0	0	0	0	0
111	3		C3	98	0	0	0	0	0	0	0	0	0	0
112	4		C4	98	0	0	0	0	0	0	0	0	0	0
113	5		C5	98	0	0	0	0	0	0	0	0	0	0
114	6		C6	98	0	0	0	0	0	0	0	0	0	0
115	7		C7	98	0	0	0	0	0	0	0	0	0	0
116	8		C8	98	0	0	0	0	0	0	0	0	0	0
117	9		C9	98	0	0	0	0	0	0	0	0	0	0
118	10		C10	98	0	0	0	0	0	0	0	0	0	0
119	11		C11	98	0	0	0	0	0	0	0	0	0	0
120	12		C12	Inactive	Inactive	Inactive	Inactive	Inactive	Inactive	Inactive	Inactive	Inactive	Inactive	Inactive
121	13		C13	Inactive	Inactive	Inactive	Inactive	Inactive	Inactive	Inactive	Inactive	Inactive	Inactive	Inactive
122	14		C14	Inactive	Inactive	Inactive	Inactive	Inactive	Inactive	Inactive	Inactive	Inactive	Inactive	Inactive
123	15		C15	Inactive	Inactive	Inactive	Inactive	Inactive	Inactive	Inactive	Inactive	Inactive	Inactive	Inactive
124	16		C16	Inactive	Inactive	Inactive	Inactive	Inactive	Inactive	Inactive	Inactive	Inactive	Inactive	Inactive
125	17		C17	Inactive	Inactive	Inactive	Inactive	Inactive	Inactive	Inactive	Inactive	Inactive	Inactive	Inactive

Figure 7: Any exposure will be allocated to the corresponding table.

There are 5 tables superimposed in the XL exposure sheet with different colours:
Blue: ''Session 1 with the ball'',
Yellow: ''Session 1 without the ball''
Orange: ''Match''
Green: ''Session 2 with the ball'',
Gray: ''Session 2 without the ball''

If you wish to enter multiple sessions, please enter the exposure of all sessions and then go and manage the individual players in the corresponding table.

For instance, if in the same day, the first of July, your **first team played a Match** (96 minutes in total) and a **subgroup of other players had 2 sessions** (one with the ball of the duration of 45 minutes and one without the ball of the duration of 60 minutes). Enter the durations of each session in the lines 4 and 5, the duration of the match in the line 6 and then go to each table to manage individual players' exposure (Figure 8).

	A	B	C	D	E	F	G	H	I	J	K
1			July 2016	Friday	Saturday	Sunday	Monday	Tuesday	Wednesda	Thursday	Friday
2	N	National Team									
3	S	Sick		1-Jul-15	2-Jul-15	3-Jul-15	4-Jul-15	5-Jul-15	6-Jul-15	7-Jul-15	8-Jul-15
4	I	**Injured** (no training)	**Session 1 with ball**	45							
5	R	**Rehab** (post-injury)	**Session 1 witout ball**	60							
6		**Incomplete Session** (enter exposure	**Match**	96							
7	A	**Absent** (Others)	**Session 2 with Ball**								
8			**Session 2 without ball**								
9	1		drthyu, wg. 01234567	45	0	0	0	0	[illegible]	[illegible]	0
10	2		sfsc, eadfwgw.	45	0	0	0	0	0	0	0
11	3		fgfg, AEFF.	45	0	0	0	0	0	0	0
12	4		sd, CDQE.	45	0	0	0	0	0	0	0
13	5		xz, htur.	45	0	0	0	0	0	0	0
14	6		cxxc, ueduda.	45	0	0	0	0	0	0	0
15	7		dfdf, caiodsj.	45	0	0	0	0	0	0	0
16	8		xzzx, .	45	0	0	0	0	0	0	0
17	9		sdfs, .	45	0	0	0	0	0	0	0
18	10		xzx, .	45	0	0	0	0	0	0	0
19	11		fgfg, .	45	0	0	0	0	0	0	0

Minutes
Enter the time in minutes for this Session 1 without the ball (in minutes from 0 to 180)

Athletes-List | 1 | 2 | 3 | 4 | 5 | 6 | 7 | 8 | 9 | 10 | 11 | 12 | Exp Summary

Figure 8: Multiple sessions per day. For instance, the team played a game of 96 min and the other players (not involved in in the match) trained twice a day, once with the ball (45 min) and once without the ball (60 min).

Session with the ball

It corresponds to a session completely dedicated to technical/tactical aspects OR to sessions performed **mainly with the ball** (in which there might be some "physical exercises").

Important: these sessions are the 'majority' of football training sessions and are mainly guided by the coaching staff (technical). Again, even if a part of the session contains exercises without the ball, this session has to be classified as "with the ball".

Session without the ball

It corresponds to sessions exclusively dedicated to physical training OR recovery (activities without the ball) and are usually guided by Fitness coaches/Physiotherapists.

3.2.2 – Players' Exposure

When the "Session" duration is set, all "active" players are automatically set as present and the duration of the session is allocated to each one of them (Figure 5).
For non-active players, the cell will display "inactive" (example: player having left the club at Mercato (players' transfer window), all the rest of the season cells for him will be displayed as "inactive").

Individual correction of players' exposure

For each session, please adapt for those players not having performed a part or the entire session.

<u>Training sessions</u>
For training sessions, you will have to correct manually the cell for the special cases (injuries, rehabilitation, absence…).

<u>Matches</u>
For matches, you will have to manage all the cases of

(i) players not exposed at all (injures, rehabilitation, absence, …).
(ii) players having had "partial exposure" (player substituted and their substitutes)
(iii) players on the bench and not involved in the game.

How to manage special cases

Absence - non exposure

1- Go to the top-left of the Excel sheet and pick-up the "Cause of absence"

B	**Bench**
N	**National Team**
S	**Sick**
I	**Injured** (no training)
R	**Rehab** (post-injury)
A	**Absent** (Others)

Tab 1

2- Choose and copy the cell corresponding to the "cause of absence"
3- Paste it on the cell corresponding to the "absence session". (this will erase the allocated session duration, which will be replaced by a "Letter/Color".

For instance: Black Cell with the Letter "S" will be allocated to a sick player.

Different types of Absences (Table 1):

Player with National Team: when a player of the Club is with the National team, his exposure with the club should be noted as ''with national team'' (see further in the manual).

Players being sick, injured or in rehabilitation shall be marked accordingly (please see table 1).

For players in the bench you should use the B option.

For all ''other'' absences please use the blue ''A'' Cell.

Partial exposure

When a player does not complete the entire session for any reason. Please go to the cell of his exposure and change the duration of exposure (Enter manually the exposure). An option to easily track these rare uncomplete sessions should be to colour the cell in yellow. Next paragraph explains how you could put a note to explain the reason of the uncomplete session.

For instance if the player g7 only 37 out of 98-min (session 1 with the ball), please change the cell in Yellow (optional), and change the exposure to ''37'' (Figure 9).

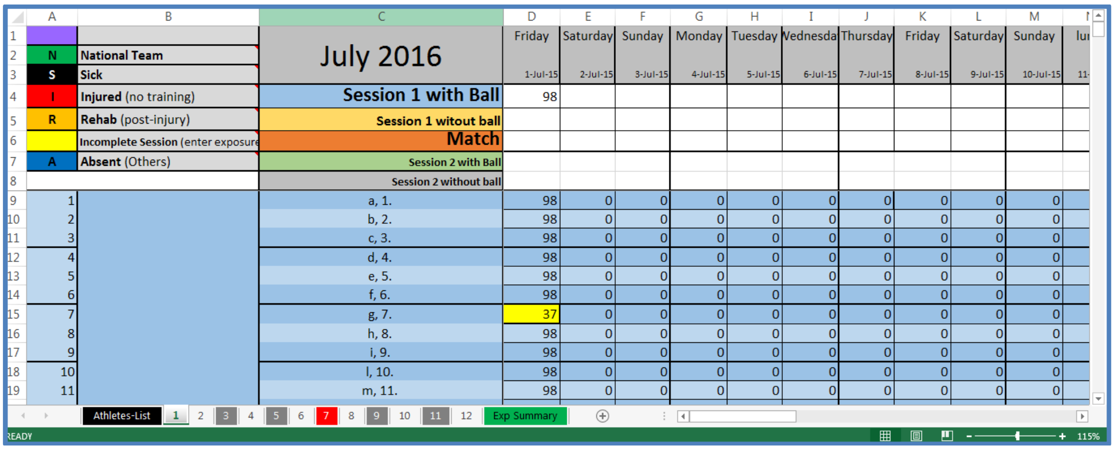

	A	B	C	D	E	F	G	H	I	J	K	L	M
1			July 2016	Friday	Saturday	Sunday	Monday	Tuesday	Wednesda	Thursday	Friday	Saturday	Sunday
2	N	National Team											
3	S	Sick		1-Jul-15	2-Jul-15	3-Jul-15	4-Jul-15	5-Jul-15	6-Jul-15	7-Jul-15	8-Jul-15	9-Jul-15	10-Jul-15
4	I	Injured (no training)	Session 1 with Ball	98									
5	R	Rehab (post-injury)	Session 1 witout ball										
6		Incomplete Session (enter exposure	Match										
7	A	Absent (Others)	Session 2 with Ball										
8			Session 2 without ball										
9	1		a, 1.	98	0	0	0	0	0	0	0	0	0
10	2		b, 2.	98	0	0	0	0	0	0	0	0	0
11	3		c, 3.	98	0	0	0	0	0	0	0	0	0
12	4		d, 4.	98	0	0	0	0	0	0	0	0	0
13	5		e, 5.	98	0	0	0	0	0	0	0	0	0
14	6		f, 6.	98	0	0	0	0	0	0	0	0	0
15	7		g, 7.	37	0	0	0	0	0	0	0	0	0
16	8		h, 8.	98	0	0	0	0	0	0	0	0	0
17	9		i, 9.	98	0	0	0	0	0	0	0	0	0
18	10		l, 10.	98	0	0	0	0	0	0	0	0	0
19	11		m, 11.	98	0	0	0	0	0	0	0	0	0

Figure 9: Managing the case of a player having had an uncomplete exposure to a session. Manually change the duration in the cell and colour the cell in Yellow.

Note: How to insert a comment (optional)

If you need to mark any comment on any cell you can put the ''cursor on the cell - then: Click right – and choose from menu: Insert comment''. You will be able to add any comment you would like to have related to this particular cell. When you complete the entering of the text, the cell will display a little small triangle red mark in its upper-right corner. Later, to see your comment, just put the cursor on that cell without clicking. This will display your ''comment''. By right-clicking again, you could, from the menu (i) delete your comment, or (ii) modify it.

3.2.3 What if the coach decides that a player should not participate in a training session?

If a player is recommended to rest due to an existing injury or so as not to aggravate an existing condition this should be marked as an injury on the attendance record (and obviously an injury card should be filled-in in the "Injury File").
If a player is recommended to rest as a safety precaution without any existing symptoms of any injury this should be marked as absence for "Other reasons" on the attendance record (no injury card required).

4. How to fill-in the "Injury Card" or "Illness Card" form

The Excel (XL) file contains 2 different spread-sheets for "Injury" and "Illness".
The File is set with some instructions on how to manage the information about injury/Illness. These appear when the cursor is placed on the corresponding cells (see fig 10).

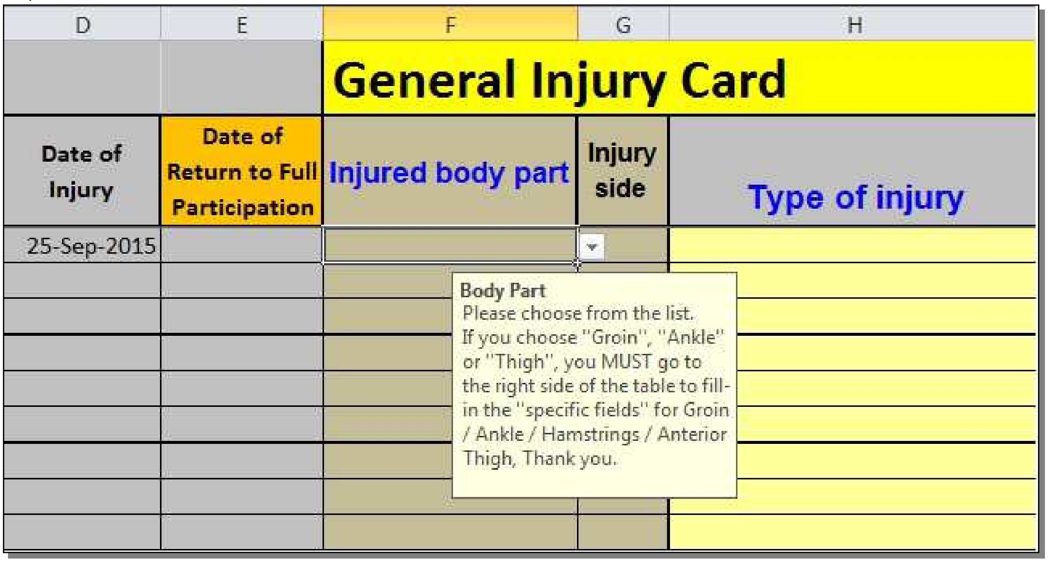

Figure 10: Pop-up "instructions" that appear when the cursor is located on a cell.

Most of the Options are set as "scrolling menus" from which the Doctor/Physiotherapist has to choose options (fig 11).

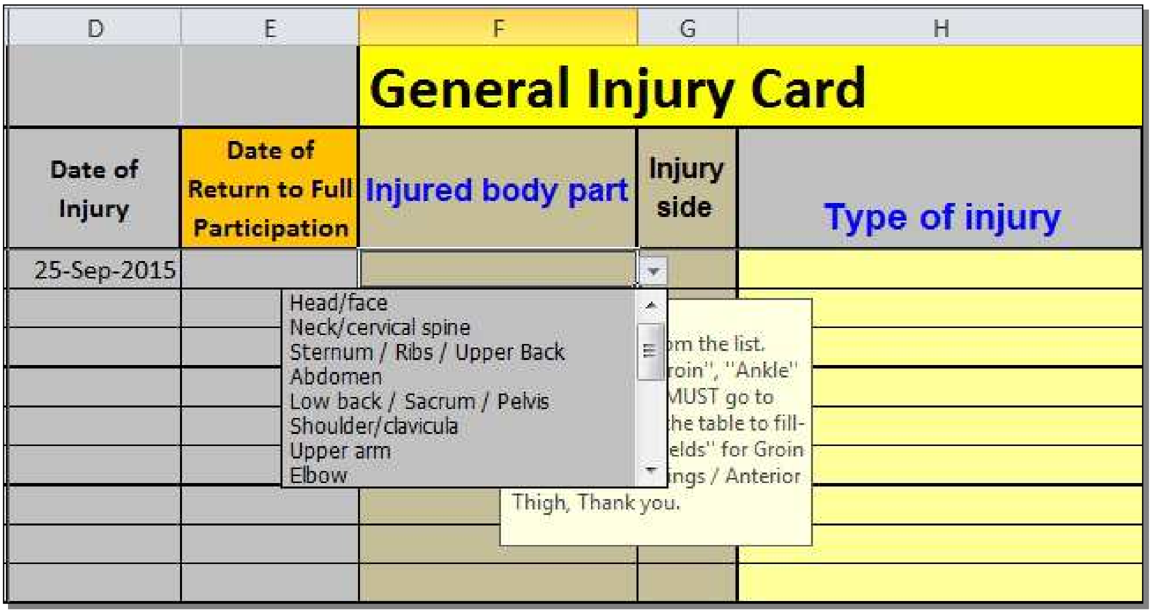

Figure 11: Scrolling menus that appear when the cell is clicked.

In some appropriate cases some "open cells" are set for adding free text (fig 12).

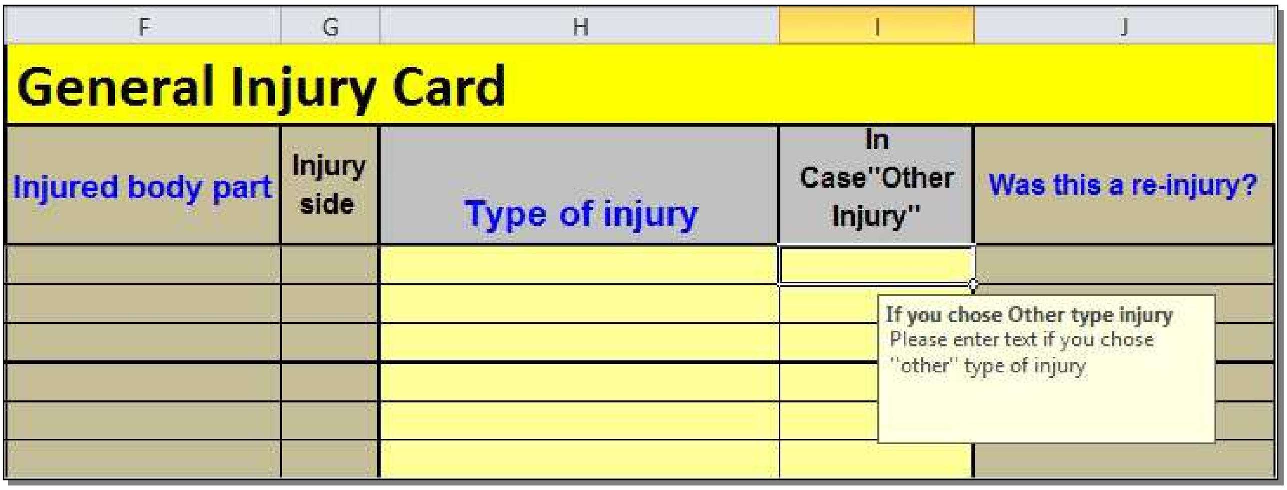

Figure 12: example of "open text cell" where the user can enter a free text.

An injury/Illness card should be filled in for every TIME LOSS injury/Illness that occurs during the season (from the first to the last day of the season, including the pre-season training period).

All fields prepared for Injury and Illness should be filled.

In some cases, if it is not possible to choose from the menu, there is the "I don't know/Unknown/N-A" option to choose from.

In some scrolling menus, at the bottom, there is the option "other". In case you tick this option, please fill in by "free text" the next column headed: "other".

Diagnosis

Write the best diagnosis you have for the moment.
If, during the following days, you discover that the diagnosis was wrong, you can modify it. The important thing is that, in the moment you send the data to the study group, you write the correct diagnosis (to your knowledge).

Estimation of hours slept during the last 24h before the "sudden onset" injuries
For "Sudden onset injuries", please estimate the number of hours slept by the player including eventual naps. If the consultation with the player is delayed and/or if the player does not remember or is not sure of this information, please tick "Unknown".

Other comments

You can write any information which can be useful for a better understanding of the injury/illness case.

Note

When the "Affected Systems" is the Upper Respiratory Tract, please provide the details of the affected areas: [Throat, Ears, and/or "Nose-Sinuses"] in the "Diagnosis" section.
When the "Affected Systems" is the Lower Respiratory Tract, please provide the details of the affected areas: [Trachea, Bronchi, Lungs] in the Diagnosis section.

4.1 Specific injury cards

For some of the more common injuries in football, specific injury cards have been created:

1 - Anterior thigh
2 - Posterior thigh (Hamstrings)
3 - Ankle Sprain
4 – Groin

These "Sections" in the XL file are located to the right of the "General Injury Card". Please fill-in any of these sections when appropriate.

4.2 Groin Card

The Groin injury card contains 4 slots for 4 possible diagnosis ("Entities").
An athlete can have more than one entity; in this case multiple entities can be entered. Please fill in first the most probable diagnosis (the "known" pain – main entity) and then, if there are more entities present, continue with the next more dominant pain, and so forth.

Please use the proposed **"Entities"** (Fig. 13):

1 - Adductor-related groin pain:
Adductor tenderness AND resisted adduction testing painful.

2 - Iliopsoas- related groin pain

Iliopsoas tenderness + more likely pain on resisted hip flexion AND / OR pain hip flexor stretching.

3 - Inguinal-related groin pain

Pain AND tenderness located in the inguinal canal region. No palpable inguinal hernia present. More likely if the pain is aggravated with resistance testing of the abdominal muscles OR on Valsalva/cough/sneeze.

4 - Pubic-related groin pain

Local tenderness of the pubic symphysis and the immediately adjacent bone. No particular resistance tests.

5 - Hip-related groin pain

Pain from the hip joint should always be considered as a possible cause of groin pain. Physical examination including passive range of motion and hip special tests - (Flexion-abduction-external rotation (FABER) and Flexion-adduction-internal rotation (FADIR) test should be performed in all cases. Tests are utilized for excluding hip-related groin pain when testing does not reproduce the athlete's pain.

6 - Other

The main categories are orthopaedic, neurological, rheumatological, urological, gastro-intestinal, dermatological, oncological and surgical, but many other rare conditions could possibly cause pain in the groin region. This entity should be utilized when the complaints cannot easily be classified into one of the common defined entities.

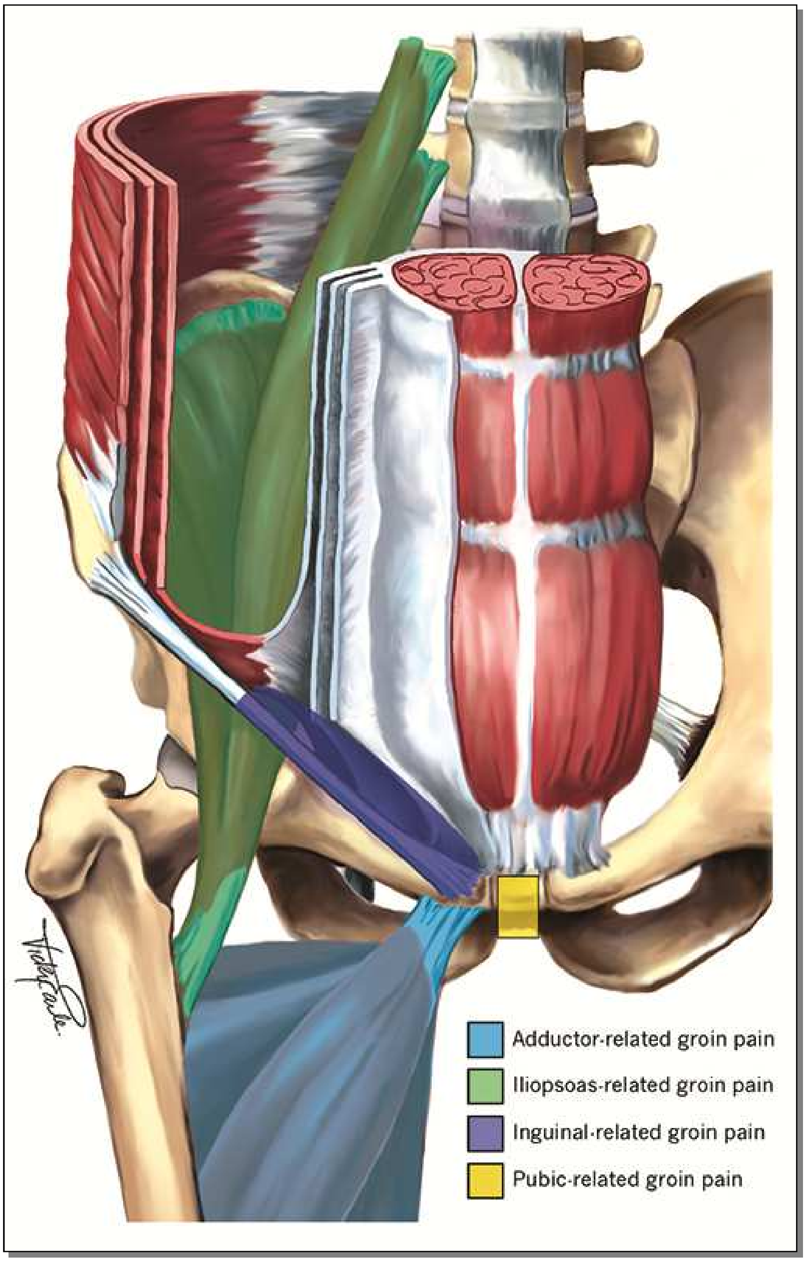

Figure 13: Illustration of the Groin Entities.

Important note:
Please do not use one of the following terms: adductor and iliopsoas tendinitis or tendinopathy, athletic groin pain, athletic pubalgia, biomechanical groin overload, Gilmore's groin, groin disruption, Hockey-goalie syndrome, Hockey groin, osteitis pubis, sports groin, sportsman's groin, sports hernia, sportsman's hernia.

5. Report Sheets:

The Reports tables allow having the REAL TIME OUTCOME of the surveillance monitoring job done by the Medical Staff.

In each XL file there is one ''report sheet''.

1 - In the Exposure file, the report (''Exp Summary'' sheet) gathers the exposures of each month (with details of matches and specific training, see fig. 14).
This sheet serves as basis of exposure for the injury and Illness rate calculation.
The numbers appearing are the displayed in minutes (product of number of players per sessions' duration).

Exposure Summary		
Month	juil-15	août-15
Availability		
Session 1 WITH ball	15380	49575
Session 1 without ball	1170	1470
Match	0	6783
Session 2 WITH ball	2900	14790
Session 2 without ball	0	0

Fig. 14: Sample of Exposure Report (please note that the names of the months will be displayed in the language set for the computer –here july and august in French (Juil/Aout).

2 - The Injury/Illness file contains also a report sheet named '' Summary Inj-Ill'' (see fig. 15).

Month	juil-15	août-15
Availability		
Count of injuries	1	17
Count of illnesses	2	3
Session 1 WITH ball	15380	49575
Session 1 without ball	1170	1470
Match	0	6783
Session 2 WITH ball	2900	14790
Session 2 without ball	0	0
Total Exposure (mins)	19450	72618
**Injuries** *per 1000 hours*	3,1	14,0
**Illnesses** *per 1000 hours*	6,2	2,5

Fig. 15: Sample of Injuries/Illness Report.

For each month the number (count) of injuries and illnesses is displayed (upper part of the table). In the bottom part of the table, the injury and illness rates (n/1000 hours of exposure) are displayed.

At the bottom of the former table, there is another table summarizing the days lost for injuries or Illnesses per month. The availability (% of the players available for training/matches) is also set (see fig. 16). This data does not take into account absences for participation to national team camps/competitions.

For specific availabiliy including the national team activity, please refer to the summary sheet of the ''Exposure XL file".

	juil-15	août-15
**Injuries**		
**Days Lost**	86	93
**Illnesses**		
**Days Lost**	28	31
**Total Days Lost**	114	124
**Availability** (exclusive of national Team)	100,0%	100,0%

Fig. 16. Report of days lost and players' availability.

6. Links between XL files

Two separate XL files have been created to allow the staff to manage 1) the Injuries/Illnesses and 2) Exposure in different files. Typically, the exposure file is used daily and could be managed by the Doctor, the Physio or the Fitness Coach. The Injuries/Illnesses file is usually managed by the Club Doctor.

1 - If ever the surveillance tools are managed by the same person in the club, we will send you a unique file combining both XL files.

2 – If ever the surveillance tools are managed by two different persons, then you will have two options to manage them.

Option 1:

You can set the link between files and therefore, be able to obtain the complete reports at any time. The procedure to set the links between files is as follows:

The XL files are linked to allow the ‘’Injury-Illness’’ file to use the data entered in the ‘’Exposure’’ file to calculate the injury and illness rates and also the players’ availability. To allow this functionality to work, save both files on the same location on the same computer. Then, open both files.
The following procedure will have to be done for the first time when opening the files on the same computer
1 – Step one: open both files.
In the ‘’Exposure’’ File go to the ‘’Exp Summary’’ Sheet. Select the table from Cell B-4 to Cell M-8. (fig. 17), and then click right: ‘’Copy’’.

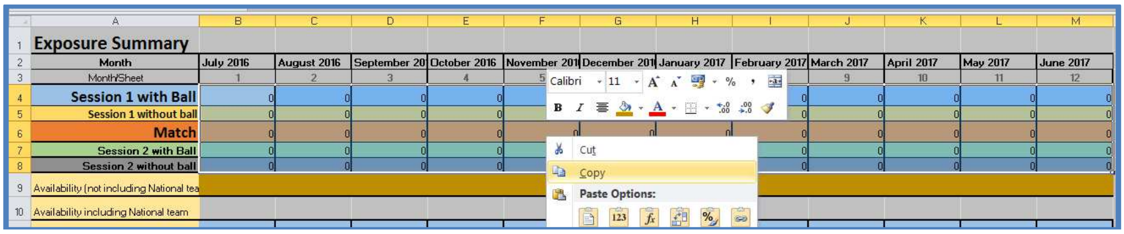

Figure 17: Selection of Exposure summary in ‘’Exposure File / Exp Summary sheet) to export to the ‘’Injuries-Illnesses’’ File.

2 – Export this selection to the ‘’Injuries-Illnesses’’ file as in figure 18.
Paste this selection in the ‘’Summary Inj-Ill’’ Sheet in Cell X (See figure 18).
Go to Cell D-4 as on the figure, click right and select ‘’Paste options’’ (far right: Paste link).

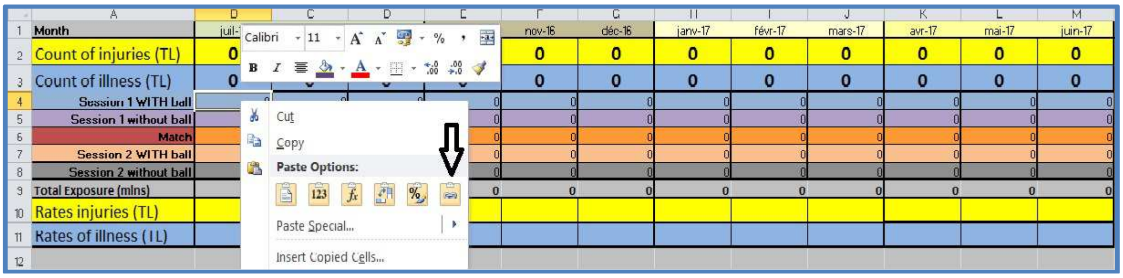

Fig 18: Pasting the selection in cell D-4 with special paste option ‘’Link’’.

Last step, export in the same way, from the same sheet of "Exposure" file to the same sheet of "Injuries-Illness" File the selection of "Players' Availability" in Row B9 to M9 (see figure 19).

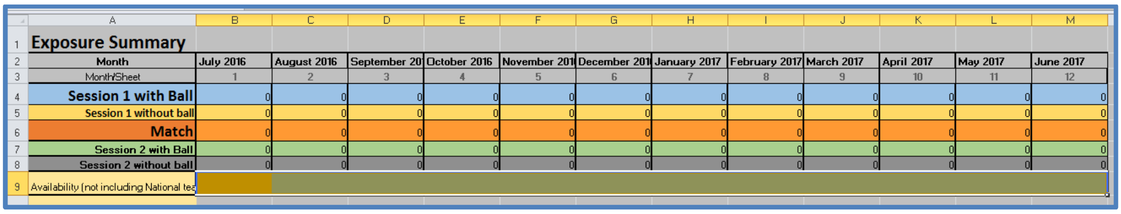

Fig. 19: Selection of Player's availability from Exposure File (Exp Summary Sheet Row B9 to M9.

Export this selection to the "injury-illness" file (sheet "Summary Inj-Ill", Cell B-20) see figure 20.

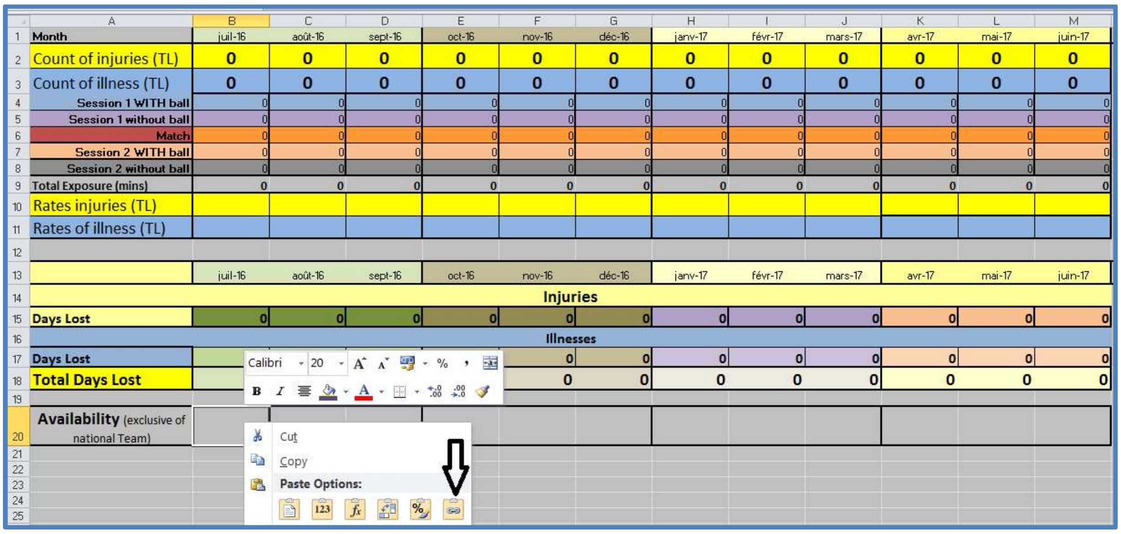

Fig. 20: Pasting the selection in cell D-20 with special paste option "Link".

Once these 2 selections imported in the "injury-illness" File, the functionality will work and you will be able to have the complete reports of the injury-Illness surveillance program.

Option 2:

If you wish you could also send the files to the Study Group once a month and we will do the links between files for you.

7. Sending the forms to the Aspetar Study Group

The e-files will be given to each Club with specific names. These will have to be used for the whole season without changing the file's name (unless when you have to anonymised, see section 3.1).

The hardcopies of each "injury" or 'illness" cards will have to be archived at the Club, as a backup. In the unlikely case that any IT issue occurs, these files will be used to re-enter the eventually lost data.
It is strongly recommended to transfer any injury/illness to the e-file as soon as possible to allow timely calculations of injury related indexes.

Every month, please send the Injuries/illnesses and Exposure XL files (anonymised) to the contact persons at Aspetar (please do not send the cards as all the information from those has already been entered on the XL Files).

7.1 How to send the Files?

Please send the two "XL files" by email to:

cristiano.eirale@aspetar.com

cc to: karim.chamari@aspetar.com and rima.tabanji@aspetar.com

7.2 When to send the forms?

PLEASE SEND THE DATA MONTHLY, most preferably during the first week of the following month.
WE KINDLY REQUEST THE USERS TO RESPECT THE DEADLINES, AS A MATTER OF DATA RELIABILITY.

7.3 Confidentiality

All data on individual players and teams will be strictly confidential.

The study group ensures that the data will stay strictly confidential (See section 6).
Each Club data will be kept at the disposal only of the "Club official contact" person (usually the team physician) for eventual retrieving.
If the Club Contact name changes, this has to be done through an official letter of the club, with approval from AFC. By no means the data will be sent back from the study group to anyone else than the Contact person.

For the sending of anonymized data, please see section 3.1 of the present booklet. In order to avoid any data ID errors, please strictly stick to the instructions given.

7.4 Questions
Do not hesitate to contact the study group at any time of the study for clarifications.
