# Supplementary material for: Lessons Learned from Implementing Injury and Illness Surveillance in Professional Football: Introducing a New Implementation Framework
Source: Sports Med. 2025 Jul 11;55(10):2375–85. doi: 10.1007/s40279-025-02276-5 (PMC12513881; doi:10.1007/s40279-025-02276-5)
Supplement: Supplementary file 7 — Appendix 6: FIFA Surveillance Manual (PDF 1566 KB) [file 40279_2025_2276_MOESM7_ESM.pdf]

Injury and Illness Surveillance

FIFA World Cup Qatar 2022™

Reporting manual – English

Contact person

Dr Andreas Serner

Medical Researcher, FIFA Medical

Tel.: +41 79 625 3899

Email: andreas.serner@fifa.org

Fédération Internationale de Football Association

FIFA-Strasse 20, P.O. Box 8044 Zurich, Switzerland

Contents

Overview 3
Surveillance period 3
Team contact person 3
Surveillance officer 3
Ethical approval and consent form 4
Player list 4
Data protection 4
Data registration and collection 5
Exposure registration 6
Definitions 6
Training exposure 6
Match exposure 6
Injury and illness registration 7
Definitions 7
Time loss 7
Return to football 7
Injury 8
Illness 8
Recurrence 8
Player action 9
Results 10
Demographic data form 11
Exposure registration form 12
Injury Registration Form 13
Illness Registration Form 15

Overview

Injuries and illnesses can affect football players' performance, careers, and future health. Our aim is to collect information on injuries and illnesses during the FIFA World Cup Qatar 2022™. This will help us improve injury and illness prevention and management in football in the future.

Surveillance period

The study will start on the first day of each national team's official team training before the tournament until the day of their last match.

The severity of injuries and illnesses is assessed based on the length of time a player is unable to play. Time loss should be recorded until the player is fully recovered, even if this is after the tournament.

Team contact person

Each team should nominate one contact person, who is on-site with the team, ideally, the team doctor, physiotherapist, or another member of the medical staff, who will be responsible for submitting the information requested, including player consent forms, training and match exposure, and injury and illness reports.

Surveillance officer

Each team will be connected to a surveillance officer dedicated to making the reporting as easy as possible. The surveillance officers speak multiple languages and will be assigned to teams accordingly. The team will be available to assist with any questions throughout the tournament and will also be able to meet at the team hotels or training facilities, if needed.

Ethical approval and consent form

The injury and illness surveillance is approved by the Swiss Association of Research Ethics Committees (BASEC no.: 2022-01790) and the Aspire Zone Foundation Institutional Review Board (AZF IRB no: E202210044). All relevant study documents are available in English, French, Spanish, and Arabic.

The purpose and procedures of the surveillance programme should be explained to the players by their team doctor, and they should be given the written study information, which is provided in non-technical language (see the enclosed file).

Players under 18 years

According to the Swiss Human Research Act (art. 23 par. 1), a research project involving adolescents capable of judgement and entailing only minimal risks does not require the informed consent of the adolescent's legal representatives. Nevertheless, players under 18 years should be advised to discuss the matter with their parents or legal guardians before giving their written informed consent.

Player list

All 26 players on the final list selected to participate in the FIFA World Cup Qatar 2022 (and any replacement players) shall be informed about the study and asked to participate.

The team contact person should provide the signed consent forms to the surveillance officer or study contact person before the first match. Individual demographic information, including age, height, weight, playing position (goalkeeper/defender/midfielder/forward) and dominant leg (preferred kicking leg), should be collected when obtaining consent.

Data protection

All data will be encrypted before analysis, and no identifiable information will be reported.

For registration, you can use either player names or a unique identification number that will be kept separate from the data collected. The information will be stored electronically in encrypted form on a secure database only accessible to authorised personnel who require the data to fulfil their duties within the scope of the research project.

Data registration and collection

Data registration is performed using simple paper forms, which take less than 5 minutes to complete.

There are four separate registration forms:

1. Demographic information
    - Demographic data will be collected initially only after the player has given consent.
2. Training and match exposure
    - Exposure should be recorded daily.
3. Injury registration
    - Injury registration forms should be completed as soon as a final diagnosis has been made.
4. Illness registration
    - Illness registration forms should be completed as soon as a final diagnosis has been made.

Your dedicated surveillance officer will be in contact with you regarding the collection of forms.
Forms can also be handed over to the FIFA Medical Coordinator at the stadium before each match.

Exposure registration

Training and match exposure should be recorded in minutes for each player for the entire duration of the tournament. No GPS measures or other tracking data is required.

For illnesses, the overall exposure is automatically calculated in days for each participant from their first day of training until the team's last match in the tournament.

Definitions

Training exposure

Training exposure is defined as any physical activities performed by the player that are aimed at maintaining or improving their skills, physical condition, and/or performance in football.

In addition to on-pitch football-specific training, individual training exposure could include gym or weight room work, running, or cycling. Pre-match warm-up and post-match cool-down should also be included as training exposure.

Match exposure

Match exposure is defined as organised scheduled match play between opposing teams (not including internal training matches).

Injury and illness registration

Data on injury and illness is gathered following the recommended methodology in the football extension of the International Olympic Committee Consensus Statement on Methods for Recording and Reporting of Epidemiological Data on Injury and Illness in Sport.

Definitions

Time loss

Time loss is defined as the inability of a player to complete the current or future training session or match. Time loss is recorded as a number of days.

Injuries and illnesses that do not result in any time loss should not be registered.

Return to football

The time to return to football is calculated from the date of injury onset (i.e. day 0) until the date when the player returned to full unrestricted team training or the date of their first partial or full match participation, if this occurs prior to the first complete team training.

Examples:

- If a player is injured during a training session and stops before the training is finished, but is able to train fully the day after, the injury should be reported with a time loss of 0 days.
- If a player is injured during a match and gets substituted (day 0), does not train fully in the following days, trains partially after four days, and completes the first unrestricted training after seven days, the injury should be reported as a time loss of seven days.
- If a player is injured during the tournament and does not return to football during the tournament, but is ready to return during the off-season when there is no team training, the return-to-football date should be when the player is considered fully recovered and would be available for full team training. During the initial registration of an injury, an estimation of the time to return to football should be recorded.

Injury

Injury is defined as tissue damage or other derangement of normal physical function resulting from rapid or repetitive transfer of kinetic energy.

Injuries should be classified according to body area, tissue, and pathology type. The diagnosis should be in writing, preferably using internationally recognised terminology and classification systems, such as the Orchard Sports Injury & Illness Classification System (OSIICS).

Additional information, such as mode of onset and injury mechanism, should also be selected on the registration form.

Illness

Illness is defined as a health-related complaint or disorder experienced by an athlete, not considered an injury.

Illnesses should be classified according to organ system/region and aetiology. The diagnosis should be in writing, preferably using internationally recognised terminology and classification systems, such as the Orchard Sports Injury & Illness Classification System (OSIICS).

Recurrence

If the injury/illness is the same type and diagnosis as a previous injury/illness, it is considered a recurrence.

If the recurrent injury was fully healed or the player fully recovered from the illness, and the player has returned to football, the subsequent injury/illness is called a re-injury/repeated illness. However, if that is not the case, it is called an exacerbation.

Player action

To understand football-specific player actions involved in injury occurrence, such actions should be reported for all sudden-onset injuries (see descriptions in the table below). We recognise that there might be some overlap between actions (for example, if a player was running with the ball and was tackled). In such cases, the player action considered most relevant for the injury should be selected.

Player action	Definition
Running	Running at any speed (acceleration/steady speed/deceleration), including linear, curved, or other types of runs with or without the ball.
Change of direction	A specific moment in a run with a sharp deviation (any angle) from the line of running with and without the ball.
Kicking	Any type of kick, including shooting/passing/crossing/set pieces/penalty.
Heading	Heading or attempting to head the ball (in a duel or alone).
Tackle	When the injured player is tackled or tackles an opponent with any body part.
Landing	Landing on one or both feet after a jump.
Falling	When a player falls or dives – for example, loses balance/stability, landing on the ground on any part of the body other than on their feet.
Controlling the ball	When a player attempts to control the ball – for example, while receiving the ball, reaching for the ball, or sliding for the ball (not sliding tackle).
Hit by ball	Any hit by the ball including a block, deflection, or accidental hits.
Collision	Players unintentionally running/jumping into each other, the goal post, or any other object or person on or around the pitch.
Other player action	For example, a throw-in, setting off in a jump, or specific goalkeeper actions not included in the other categories.
Unknown	

Results

After the World Cup, we will send you a summary of the overall results. The results will only be presented as group data, and no team or individual player can be identified. The results will also be presented internally within FIFA, shared with key stakeholders and submitted for publication in an international sports medicine journal. They may be further disseminated externally, including through various media outlets, press conferences and at scientific conferences.

All responsible team medical representatives will be acknowledged as part of the FIFA World Cup Qatar Medical Teams Group.

Thank you for your participation!

Demographic data form

Player ID	Age (years)	Height (cm)	Weight (kg)	Playing position	Dominant leg

Exposure registration form

Player ID	Date: _________ Training/match (mins)	Date: _________ Training/match (mins)	Date: _________ Training/match (mins)	Date: _________ Training/match (mins)	Date: _________ Training/match (mins)	Date: _________ Training/match (mins)

Injury Registration Form

Player ID		Date of injury	

Injured body area
(please complete additional injury forms if multiple body areas are injured)

☐ Head/face	☐ Elbow	☐ Chest	☐ Hip	☐ Lower leg
☐ Neck/cervical spine	☐ Forearm	☐ Thoracic spine	☐ Groin	☐ Ankle
☐ Shoulder/clavicula	☐ Wrist	☐ Lumbosacral	☐ Thigh	☐ Foot
☐ Upper arm	☐ Hand	☐ Abdomen	☐ Knee	☐ Unspecified

Injury side

☐ Right	☐ Left	☐ Bilateral/central

Injured tissue	Injury type	
☐ Muscle/tendon	☐ Muscle injury<br>☐ Muscle contusion<br>☐ Muscle compartment syndrome	☐ Tendinopathy<br>☐ Tendon rupture
☐ Nervous	☐ Brain/spinal cord injury	☐ Peripheral nerve injury
☐ Bone	☐ Fracture<br>☐ Bone stress injury<br>☐ Bone contusion	☐ Avascular necrosis<br>☐ Physis injury
☐ Cartilage/synovium/bursa	☐ Cartilage injury<br>☐ Arthritis	☐ Synovitis/capsulitis<br>☐ Bursitis
☐ Ligament/joint capsule	☐ Joint sprain (ligament tear or acute instability)	☐ Chronic instability
☐ Superficial tissues/skin	☐ Contusion (superficial)<br>☐ Laceration	☐ Abrasion
☐ Vessels (vascular trauma)		
☐ Stump (stump injury)		
☐ Internal organs (organ trauma)		
☐ Non-specific (injury without tissue type specified)		

Specific diagnosis

Specify your diagnosis following all examinations:

Examinations (check all that apply)

☐ Clinical only (no imaging)	☐ Ultrasonography	☐ Surgery
☐ X-ray	☐ Magnetic resonance imaging	☐ Other (specify):

When did the injury occur?

☐ Training	☐ Match (specify min. of injury):	☐ Other:	☐ N/A (gradual onset)

Injury mechanism

Was the onset of symptoms sudden or gradual?		☐ Sudden onset	☐ Gradual onset
What was the mechanism of injury?		☐ Acute mechanism	☐ Repetitive mechanism
Was the injury caused by contact?	☐ No	☐ Yes, direct contact (to injured body part)	☐ Yes, indirect contact (to another body part)

For contact injuries – contact by:	☐ Opponent	☐ Team-mate	☐ Match official
	☐ Pitch invader	☐ Other pitch-side staff	☐ Other
	☐ Ball	☐ Goalpost	☐ Pitch object
	☐ Object from the crowd	☐ Other object	☐ Unknown

Player action at the time of injury (sudden-onset injuries only)

☐ Running (any speed)	☐ Tackle	☐ Hit by the ball
☐ Change of direction	☐ Landing	☐ Collision
☐ Kicking (any type)	☐ Falling	☐ Other:
☐ Heading	☐ Controlling ball	☐ Unknown

Injury mechanism (describe in words):

Other information

Was this a recurrence?	☐ No	☐ Yes (provide date of return from previous injury) :		☐ Unsure
If yes, was this an exacerbation or re-injury?	☐ Exacerbation	☐ Re-injury	☐ Not applicable	
Referee's sanction:<br>(sudden-onset match injuries only)	☐ No foul<br>☐ Opponent foul<br>☐ Own foul	Opponent card:<br>☐ No card<br>☐ Yellow card<br>☐ Red card	Injured player card:<br>☐ No card<br>☐ Yellow card<br>☐ Red card	
Other comments (if any):				

Time loss

Expected time loss from football in days:	Actual time loss from football in days:

Illness Registration Form

**Player ID**		**Date of illness**	

**Organ system/region** (please complete additional illness forms if multiple illnesses are diagnosed)		
☐ Cardiovascular	☐ Gastrointestinal	☐ Otological
☐ Dermatological	☐ Genitourinary	☐ Psychiatric/psychological
☐ Dental	☐ Neurological	☐ Respiratory
☐ Endocrinological	☐ Ophthalmological	☐ Thermoregulatory
☐ Unknown or not specified		

**Aetiology**		
☐ Allergic	☐ Infection	☐ Degenerative or chronic condition
☐ Environmental – exercise-related	☐ Neoplasm	☐ Developmental anomaly
☐ Environmental – non-exercise	☐ Metabolic/nutritional	☐ Drug-related/poisoning
☐ Immunological/inflammatory	☐ Thrombotic/haemorrhagic	☐ Unknown or not specified

**Specific diagnosis**
Specify your diagnosis following all examinations:

**Examinations** (check all that apply)		
☐ Clinical	☐ Ultrasonography	☐ Laboratory (specify):
☐ X-ray	☐ Magnetic resonance imaging	☐ Other (specify):

**Other information**			
Was this a recurrence?	☐ No	☐ Yes (provide date of return from previous illness):	☐ Unsure
Other comments (if any):			

**Time loss**	
Expected time loss from football in days:	Actual time loss from football in days:
